# Synopsis of *Habenaria* s.l. (Orchidaceae) in New Guinea and adjacent islands

**DOI:** 10.7717/peerj.12011

**Published:** 2021-09-10

**Authors:** Marta Kolanowska, Marta Kras, Sławomir Nowak, Dariusz L. Szlachetko

**Affiliations:** 1 Department of Biodiversity Research, Global Change Research Institute AS CR, Brno, Czech Republic; 2 Department of Geobotany and Plant Ecology, Faculty of Biology and Environmental Protection, University of Lodz, Lodz, Poland; 3 Department of Plant Taxonomy and Nature Conservation, University of Gdańsk, Gdańsk, Poland

**Keywords:** Orchids, Biodiversity, New species, Taxonomy

## Abstract

A taxonomic synopsis of the orchid genus *Habenaria* in New Guinea and adjacent islands is presented. We confirmed the occurrence of 27 *Habenaria* species in study area. Sixteen of these are endemic and were not so far found outside New Guinea. Morphological characteristics and illustrations of floral segments of taxa are presented. One new species of *Habenaria* is described. Four neotypes are selected. An updated key to species groups and species occurring in the study area is provided. The importance of diagnostic floral characters in *Habenaria* is discussed.

## Introduction

The area encompassing New Guinea island together with the Admiralty and Bismarck Archipelagos and the Solomon Islands is generally called the Papuasia region. Located on the Sahul shelf between latitudes 0019′–10043′ South and longitudes 130045′–150048′ East, New Guinea is the highest tropical island with the summit of Mt. Jaya reaching 4,880 m and the second largest island in the world. New Guinea is young and tectonically dynamic (Galloway & Loffler, 1972). As a result of shifts of tectonic plates and significant climate changes, an extraordinary variety of ecosystems is observed in the island. Stevens (1989) divided New Guinea into six areas or sub-regions: the southern plains, the central cordillera, the Vogelkop and islands, the northern basin (inter montane troughs) and ranges, the northeastern islands and the southeastern islands. In the context of biodiversity, the most important habitats are wet tropical lowlands, mesic forest, seasonal dry forest, savanna, alpine grassland and “tundra” (alpine mosslands as defined by Wade & McVean (1969), Sherman & Bryan (2011) and Hope (2014)). New Guinea is considered as one of the most species rich areas in the world with high level of endemism of about 68% (Cámara-Leret et al., 2020; Kreft & Jetz, 2007; Mutke & Barthlott, 2005). Heads (2001) indicated that while the biodiversity of the northern part of New Guinea is mainly influenced by the Indonesia-Malayan region, the flora of the southern part harbours many plants of Australian origin.

According to Cámara-Leret et al. (2020) the New Guinea island is home to as many as 13,634 plant species, however, this number seems to be underestimated as numerous new taxa are described from the region frequently (*e.g*. Parsons, 1996; Forster & Smith, 2010; Lyon, 2016). Orchidaceae, with ca. 2,800 species constitutes an important part of regional flora. Noteworthy, about 86.3–95% of these are endemic to New Guinea (Cámara-Leret et al., 2020; Vollering et al., 2016). Without doubt many orchids still await discovery and the specific composition of numerous genera requires revision. The first comprehensive works on Orchidaceae from the region were published by Schlechetr (1914) and Johannes Jacobus Smith (*e.g*. Smith, 1903, 1909). More recent orchid floras were presented by Schuiteman, Vermeulen & De Vogel (2001–2010), Ormerod (2017) and De Vogel, Vermeulen & Schuiteman (in Press).

One of those genera is *Habenaria* Willd. which with almost 900 species (Govaerts et al., in Press) is one of the largest and most widespread within Orchidaceae. The highest diversity of *Habenaria* is observed in Brazil, southern and central Africa and East Asia (Kurzweil & Weber, 1992). These are mostly terrestrial herbs with fleshy tubers and variously arranged leaves. Their flowers are usually resupinate, with the dorsal sepal forming a hood with the petals (Xinqi & Cribb, 2009). In New Guinea, while some of the genus representatives have adapted to a broad range of habitats and are relatively widespread in the island, others are confined to a very restricted distribution area. Schlechter described 16 species of *Habenaria* from New Guinea and Smith added four taxa. Renz (1987) also contributed to the knowledge of the genus in New Guinea and adjacent islands. The present work complements previous studies by providing keys to identification, a revision of nomenclatural types status, and a reference list of herbarium specimens representing the studied species.

Our work is a first synopsis of the genus in New Guinea and adjacent areas. The information about each species includes morphological description, information about habitat, ecology and local distribution. Keys to identification of morphological groups and species are provided.

## Materials & methods

This work was conducted using classical taxonomic methods. Herbarium specimens were used from the following herbaria: A, AMES, B, BM, BO, BR, E, K, L, LAE, NY, P, RENZ, UPNG and US (Thiers, 2020). The flowers of examined plants were rehydrated, with their taxonomically important details drawn, measured and described. Preferably flowers from the central part of inflorescences were used as this is the most common approach for morphometric studies (Bateman & Rudall, 2006). Flowers from other inflorescence parts were used when these in the mid-point were poorly preserved. Each specimen was photographed. After the database had been compiled, the data were compared with the original material—types, diagnoses and/or illustrations, if available. A total of ca 200 specimens of *Habenaria* were examined. Information on the ecology, distribution and phenology of individual species was obtained from herbarium labels or extracted from the literature as much as possible. The list of reference herbarium specimens is provided in File S1. Distribution maps were created using ArcGis 10.6 (Esri, Redlands, CA, USA) based on location data provided on herbarium sheet labels. Georeferencing of some records was not possible to the scarce information about the location of collected specimens. Each record was assigned to one of terrestrial ecoregions as described by Olson et al. (2001). Altitudinal range chart of was prepared using MS Excel. Species richness was calculated using DIVA-GIS 7.5.0.0. (Hijmans, Guarino & Mathur, 2012).

### Nomenclature

The electronic version of this article in portable document format will represent a published work according to the International Code of Nomenclature for algae, fungi, and plants (Turland et al., 2018), and hence the new names contained in the electronic version are effectively published under that Code from the electronic edition alone. In addition, new names contained in this work that have been issued with identifiers by IPNI will eventually be made available to the Global Names Index. The IPNI Life Science Identifiers (LSIDs) can be resolved and the associated information viewed through any standard web browser by appending the LSID contained in this publication to the prefix “http://ipni.org/.” The online version of this work is archived and available from the following digital repositories: PeerJ, PubMed Central and CLOCKSS.

The genus *Habenaria s.l*. is one of the most numerous and due to the high similarity between species it often causes problems in identification and consequently in understanding the concept of species. In this paper, we decided to revise the status of nomenclatural types for the names of the species studied to ensure the stability of their concepts and to facilitate future research. Each specimen designated as a lectotype or neotype has been examined and presents a set of relevant characters for the taxon. Altogether, we designated five lectotypes and four neotypes. For five species, *H. notabilis* Schltr., *H.retroflexa* F.Muell. & Kraenzl., *H. micholitziana* Kraenzl., *H. dracaenifolia* Schltr. and *H. dryadum* Schltr., we have decided not to make any decision, as the concepts of these species are not yet fully clear or examined materials are not sufficient and most likely these names requires neotypification.

## Results

We confirmed the occurrence of 27 *Habenaria* species in New Guinea and adjacent islands. Sixteen taxa, including one new species described here, are endemic for New Guinea. They occur almost always as terrestrial herbs in montane grasslands, savannas and forests at altitudes of 20–3,260 m.

**Figure 1:**
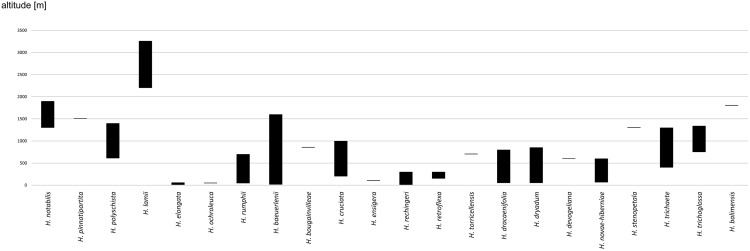
Altitudinal ranges of *Habenaria* in New Guinea and adjacent islands.

**Figure 2:**
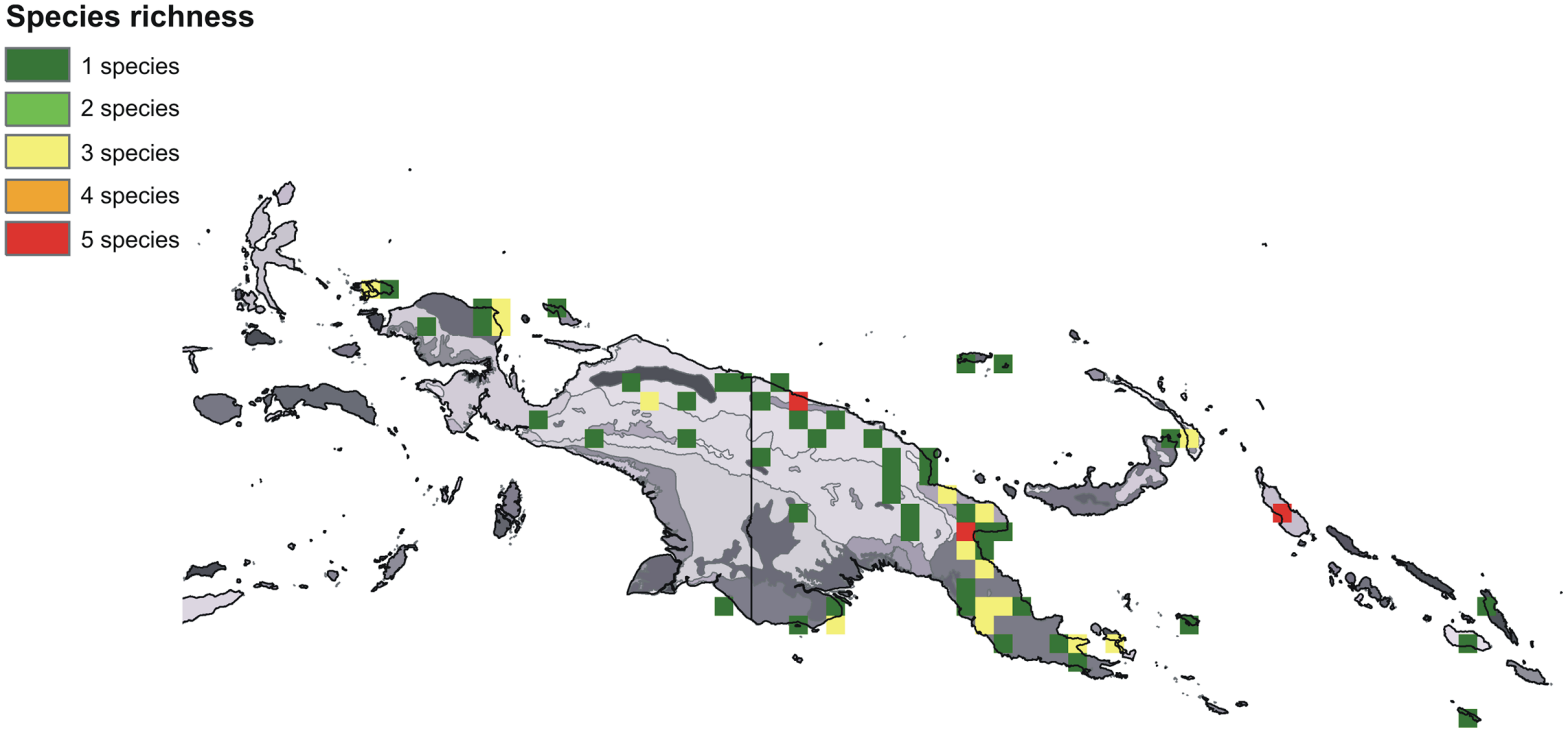
Richness of *Habenaria* species in New Guinea and adjacent islands. Base map of ecoregions developed by Olson et al. (2001) and downloaded from https://www.sciencebase.gov/catalog/item/508fece8e4b0a1b43c29ca22.

The broadest altitudinal range is observed in *H. baeuerleni* (15–1,600 m) and *H. lamii* (2,200–3,260 m). Numerous *Habenaria* representatives are restricted in their elevational distribution, *e.g. H. balimensis, H. bougainvilleae, H. devogeliana, H. ensigera, H. ochroleuca, H. pinnatipartita, H. stenopetala, H. torricellensis* (Fig. 1). Some species are known from single localities, *e.g. H. ensigera, H. balimensis, H. bougainvilleae, H. devogeliana, H. drepanodes, H. khasiana, H. micholitziana, H. pinnatipartita*, and *H. retroflexa*. The most widely distributed are *H. baeuerlenii, H. dryadum, H. rumphii* and *H. tichoglossa*. The highest richness of species is observed in Torricelli Mountains, in Huon Gulf area (Morobe province) as well as in Solomon Islands (Fig. 2). Considering ecoregional variation, the most diverse in *Habenaria* species are Southeastern Papuan rain forests (eight species), Central Range montane rain forests (seven species), and Northern New Guinea montane rain forests (seven species).

### Taxonomic treatment

***Habenaria*** Willd., Sp. Pl. 4: 44. 1805; Generitype: *Habenaria macroceratitis* Willd.

Tubers 1 or few, usually ovoid or ellipsoid. Leaves cauline, decreasing in size upwards, or basal, almost radical, ground-hugging, usually sessile or subsessile. Inflorescence usually many-flowered. Flowers of various size, but most often small to medium, resupinate. Sepals similar or dissimilar. Petals bi-partite or entire, ciliate or glabrous, partially adnate to the Dorsal sepal forming a galea or completely free. Lip usually 3-lobed, lobes similar or dissimilar, usually linear or lanceolate, pendent or diverging. Spur filiform to cylindrical, of various length and shape. Gynostemium short and massive. Anther erect or bent back up to an angle of 90°, rounded at the apex and elongated at the base in short processes (=antherophores). Connective usually truncate, rather broad. Pollinia 2, obovoid to ellipsoid. Caudiculae elastic, usually as long as or longer than pollinia. Auriculae usually small, sometimes bilobed or very conspicuous, large. Stigma bi-lobed, both lobes forming a prominent but relatively short stigmaphores, often pendent, with most of the upper surface fertile. Rostellophores subequal in length to antherophores. Viscidia rather small.

This cosmopolitan genus in the broad sense comprises about 800 species distributed in most parts of the world except Antarctica.

### Key to groups of species

1. Lip lateral lobes more or less pinnate, or with filiform projections … ***H. polyschista*-group****(1)**
  1* Lip lateral lobes entire (shortly and irregularly bilobulate-emarginate in *H. ensigera*), without any projections(2)
2. Petals unlobed, entire3 ***H. elongata*-group (2)**
  2* Petals bilobed5
3. Leaves cauline***H. elongata*-group**, ***H. lamii*-subgroup**
  3* Leaves basal, rosulate4
4. Leaves narrow, linear to narrowly lanceolate***H. elongata*-group**, ***H. elongata*-subgroup**
  4* Leaves oblanceolate to obovate-elliptic***H. elongata*-group**, ***H. torricellensis*-subgroup**
5. Leaves gathered near the middle of the stem, which is covered below by bladeless sheaths***H. dracaenifolia*-group (3)**
  5* Leaves arranged along the stem***H. balimensis*-group (4)**

1. ***Habenaria polyschista*-group**

Plants with stem basally covered with leaf sheaths, leafy above. Leaves lanceolate to narrowly elliptic. Petals entire to pinnate. Lip lateral lobes and petals anterior lobes deeply dissected, more or less pinnate. Gynostemium relatively short and massive, anther ellipsoid, stigmaphores and rostellophores very short, filiform, stigmaphores little longer, ligulate, auricles very large, prominent.

### Key to species of *Habenaria polyschista*-group

1. Floral bracts acuminate, lip lateral lobes apically dilated and dissected into a fascicle of numerous aristate segments1.1. ***H. notabilis***
  1* Floral bracts long-acuminate to caudate, lip lateral lobes filiform, without any widening, with filiform segments not gathered in a fascicle2
2. Lip lateral lobes and anterior petal lobes pinnate1.4. ***H. polyschista***
  2* Lip lateral lobes with few, filiform outgrowths, petals entire or with few filiform or subulate segments3
3. Anterior petal lobe almost entire, lip lateral lobes with few subulate projections and short, irregular teeth, spur without any dorsal rib1.2. ***H. paucipartita***
  3* Anterior petal lobe with a single filiform segment, lip lateral lobes with 2-3 patent, unequal, filiform lobules and sometimes near the apex, spur inflated part dorsally with an obtuse longitudinal rib1.3. ***H. pinnatipartita***

1.1. ***Habenaria notabilis*** Schltr., Repert. Sp. Nov. Regni Veg., Beih. 1: 15. 1911. TYPE: Papua New Guinea [Deutsch Neu-Guinea]. *R. Schlechter 18556* (B†). ≡ *Medusorchis notabilis* (Schltr.) Szlach., Orchidee (Hamburg) 55: 489. 2004.

**Figure 3:**
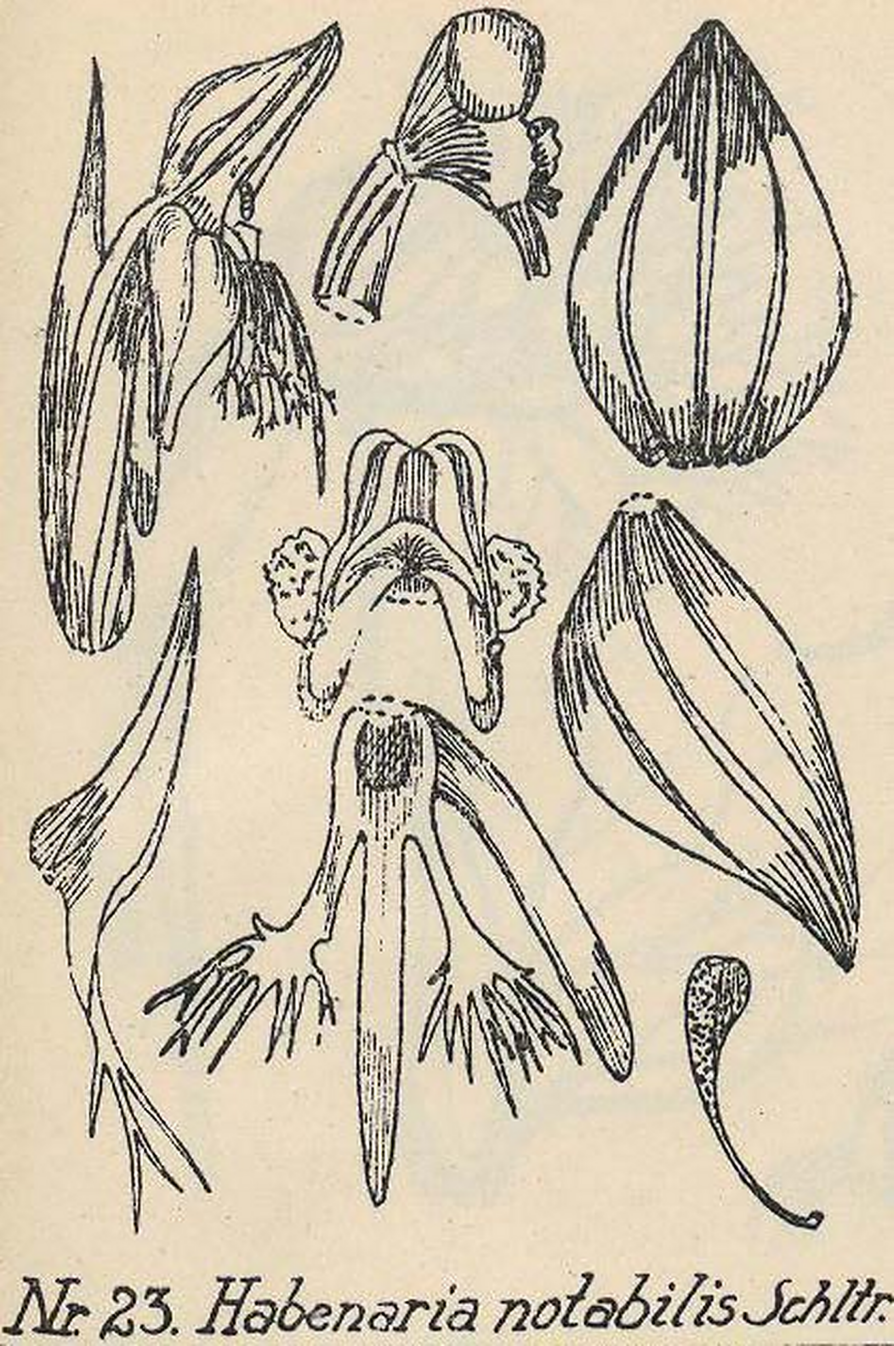
*Habenaria notabilis* Schltr., original Schlechter’s drawing from Figurenatlas zu den Orchidaceen von Deutsch-Neu-Guinea, Feddes Repert. Spec. Nov. Regni Veg. Beih. 21(1), 1923 (Plate VIII, nr 23). URL: https://www.biodiversitylibrary.org/page/57688227.

Plant up to about 130 cm tall, leafy except for the base which is covered with sheaths only. Leaves erect-patent, up to 30 cm long and 4.5 cm wide, lanceolate-ligulate, acuminate, upper ones gradually transforming into bracts. Inflorescence about 20 cm long, densely many-flowered. Floral bracts up to 20 mm long, lanceolate, acuminate. Pedicel with ovary 16 mm long, cylindrical. Flowers green, petals and lip yellowish. Dorsal sepal 9.5 mm long, 6 mm wide, concave, ovate, obtuse, 3-veined. Lateral sepals up to 11 mm long, 6 mm wide, deflexed, obliquely ovate, acute, 3-veined. Petals bilobed almost to the base; anterior lobe ca 9.5 mm long, decurved, linear, irregularly split into 3–4 awn-like processes in front; posterior lobe about 9.5 mm long, falcate-lanceolate, acuminate-subulate. Lip 3-lobed just above the base, 13 mm long in total; middle lobe about 12 mm long, linear, narrowly obtuse, entire; lateral lobes about 10 mm long, linear in basal part, dilated towards the apex, and here irregularly split into 7–10 aristate partitions. Spur 13 mm long, pendulous, cylindrical, obtuse. Gynostemium 2.5–3 mm long, short, rather massive; stigmaphores almost twice surpassing the anther channels. Fig. 3.

*Habitat:* Terrestrial in leaf-litter in lower montane forest. Alt. 1,300 m.

*Distribution*: New Guinea.

**Figure 4:**
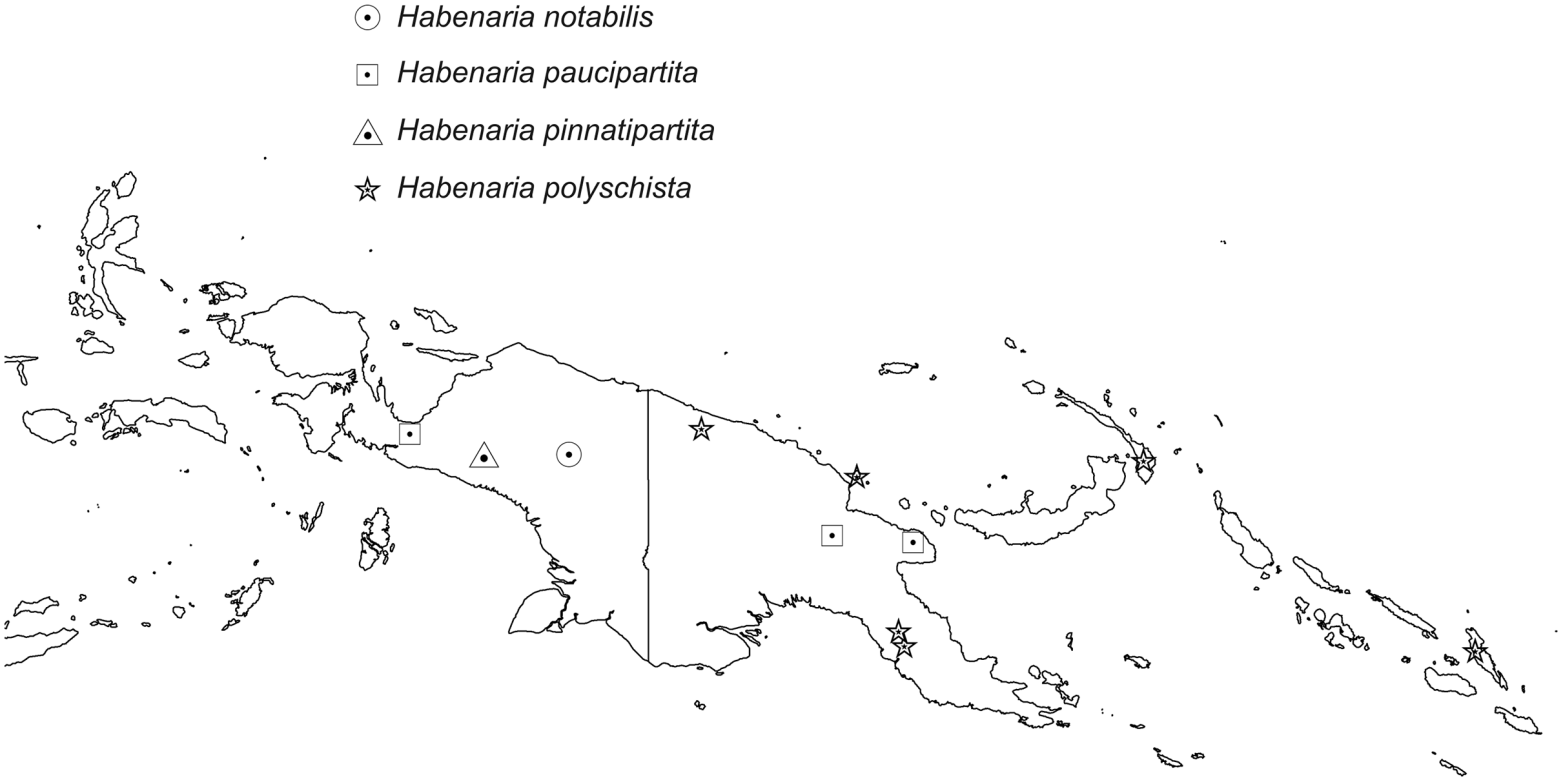
Distribution of *Habenaria notabilis, H. paucipartita, H. pinnatipartita* and *H. polyschista*. Base map downloaded from www.naturalearthdata.com.

*Notes:* Type material of *Habenaria notabilis* is known exclusively from the original Schlechter’s drawing and description. It can be easily separated from all other representatives of New Guinean *Habenaria* by the peculiar lip form, with the lateral lobes apically dilated and ornamented by a fascicle of aristate projections. In other species of the *H. polyschista*-group outgrowths can be found along both margins of the lateral lobes of the lip.

*Representative specimens*: Papua New Guinea. Kaiser-Wilhelms Land. In humus der Wälder des Bismarckgebirges. Alt. 1,300 m. Oct 1908. *R. Schlechter 18556* (B†). Indonesia. Prov. Papua. Snow Mountains region. E of the Baliem Valley, Kab. Jayawijaya, Kec. Kurima. Alt. 1,900 m Oct 1992. *W. Milliken 1481 p.p*. (K!). Fig. 4.

*Milliken 1481* (K) consists of two sheets, however sheet II with vegetative parts does not match the genus *Habenaria* and was possibly mixed up.

1.2. ***Habenaria paucipartita*** J.J. Sm., Meded. Herb. Leid 23: 1. 1915. TYPE: Papua New Guinea (Deutsch Neu-Guinea). *R.F. Janowsky 339* (Lectotype, *designated here*: BO!).

**Figure 5:**
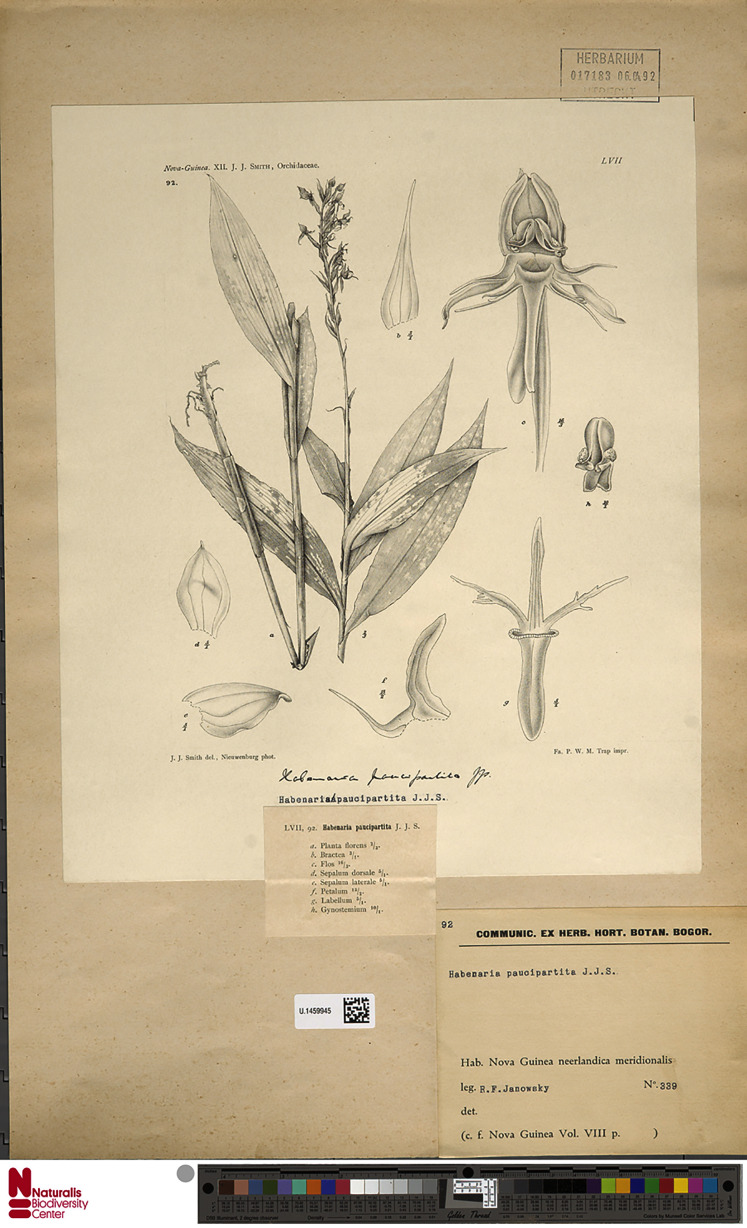
*Habenaria paucipartita* J. J. Sm., herbarium sheet with original Smith’s drawing at Naturalis Biodiversity Center, U.1459945. (CC0 1.0; https://data.biodiversitydata.nl/naturalis/specimen/U.1459945).

**Figure 6:**
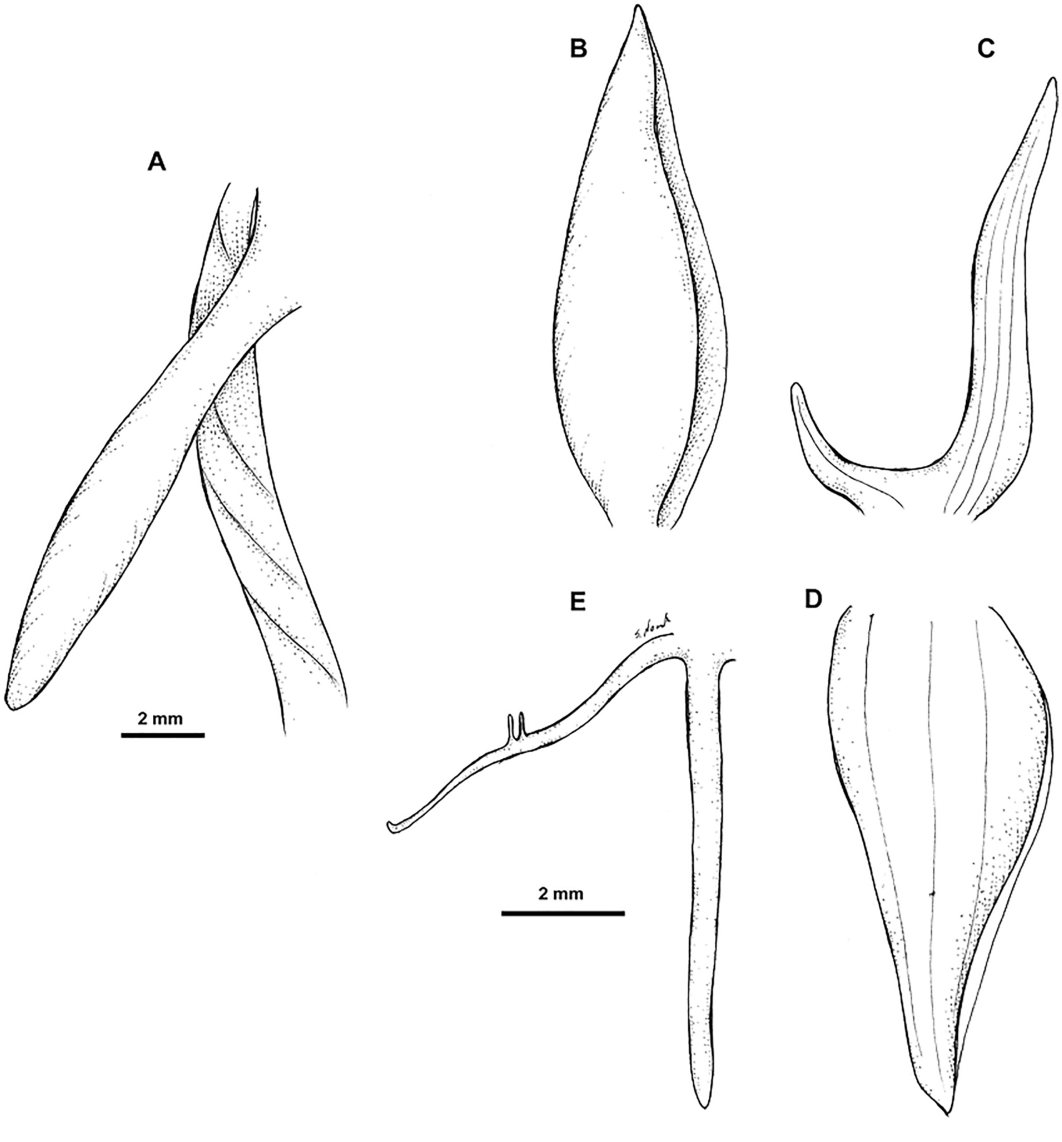
*Habenaria paucipartita* J. J. Sm.: (A) ovary and spur; (B) dorsal sepal; (C) petal; (D) lateral sepal; (E) lip (drawn from *Katik LAE77817*; LAE).

Plant about 70.0 cm tall, in basal half or 2/3 covered with tubular sheaths only, upper part leafy. Leaves about 8, patent, 8.0–17.0 cm long, 2.0–3.5 cm wide, lanceolate, acuminate, long mucronate, narrowed at the base; sheaths tubular. Inflorescence 21.0 cm long, laxly ca. 20-flowered, peduncle with about 4 leaf-like scales. Floral bracts up to 20.0 mm long, ovate, long acuminate. Pedicel with ovary 16.0 mm long. Flowers green with cream lip. Dorsal sepal up to 8.3 mm long, 5.0 mm wide, concave, ovate-elliptic, very shortly acuminate, subulate-apiculate, 3-veined. Lateral sepals 9.0 mm long, 4.3 mm wide, obliquely ovate, at the apex channelled-contracted, apiculate, 3-veined. Petals deeply bilobed just above the base, basally 3.0 mm wide; anterior lobe 8.0–8.7 mm long, linear-subulate, gently upcurved, 1-veined; posterior lobe up to 7.0 mm long, linear, angular-falcate, narrowly obtuse, subretuse, basally obliquely dilated, 2-veined. Lip 3-lobed just above the base; middle lobe 8.5–9.5 mm long, convex, linear, subobtuse, 3-veined; lateral lobes 6.5–8.0 mm long, divergent, narrowly linear, in the middle with a few subulate projections and short, irregular teeth. Spur about 10.0 mm long, subcylindrical, slightly clavate, obtuse. Gynostemium about 3.5 mm long, stigmaphores oblong, surpassing the anther channels. Figs. 5–6.

*Habitat:* Terrestrial in montane forest (Schuiteman, Vermeulen & De Vogel, 2001–2010) at the altitude of 1,800–2,100 m.

*Distribution*: New Guinea.

*Representative specimens*: Indonesia. Prov. Papua. Geelvink-Bai, Jabi-Gebirge bei Wappe. May 1913. *R.F. Janowsky 339* (BO!). Papua New Guinea. Eastern Highlands Province. 7 Sep 1975. *M.J. Sands 1742* (K!); Morobe. Jakupet village, Finschhafen Subprovince. 6°15′S 147°20′E. Aug 1984. *P. Katik LAE77817* (LAE!, NSW). Fig. 4.

*Notes: Habenaria paucipartita* resembles *H. pinnatipartita*. In fact, both species share similar size and habitat, similar form of the flower segments, except lip and petals form. Both can be distinguished by the number of filiform outgrowths of lip lateral lobes and anterior petal lobes. In *H. paucipartita* the lip lateral lobes are ornamented in the middle with a few subulate projections and short, irregular teeth, whereas in *H. pinnatipartita* both lateral lobes are provided with three patent, unequal, filiform lobules and sometimes the front margin also with a very short lobule. The anterior petal lobes of the former species are entire, usually without any projection. In the latter species the front margin of the lip lateral lobes is provided with a similar lobule which is more or less shorter and sometimes with an additional tooth-like lobule. Additionally, the spur of *H. paucipartita* is shorter (up to 10 mm long *vs* 16 mm long), slightly clavate and obtuse (*vs* prominently inflated in the apical part, attenuate towards the apex). The unique characters of *H. pinnatipartita* are the spur with an obtuse longitudinal rib on the dorsal surface of the inflated part and the shortly clawed lip. Neither of these characters can be found in *H. paucipartita*.

In *Katik 77817* specimen the petal posterior lobe is much shorter than in other specimens (less than 3 mm long), however, based on the lack of any additional outgrows on the petals and presence of few, very short filiform projections on the lip lateral lobes it was identified as *H. paucipartita*.

We found only one specimen cited in the protologue (*Janowsky 339*, BO), but the author did not indicate whether the new taxon was described solely on this material. In that case the designation is not sufficient to consider this collection as a holotype under Article 9.1. Ex. 1 of the ICN (Turland et al., 2018). In addition, after a detailed review of the literature, no other author has identified this material as a type, holotype or lectotype unintentionally in an effective publication before 1. January 2001 (Art. 9.10) or intentionally after this date (Art. 9.12 and 9.23). For that reason we decided to designate the specimen of *Janowsky 339* (BO) as lectotype to avoid future ambiguities.

1.3. ***Habenaria pinnatipartita*** J. J. Sm., Nova Guinea, Bot. 18: 9. 1935. TYPE: Indonesia. *W. M. Docters van Leeuwen 10935* (Lectotype, *designated here*: L!, Isolectotype: RENZ!).

**Figure 7:**
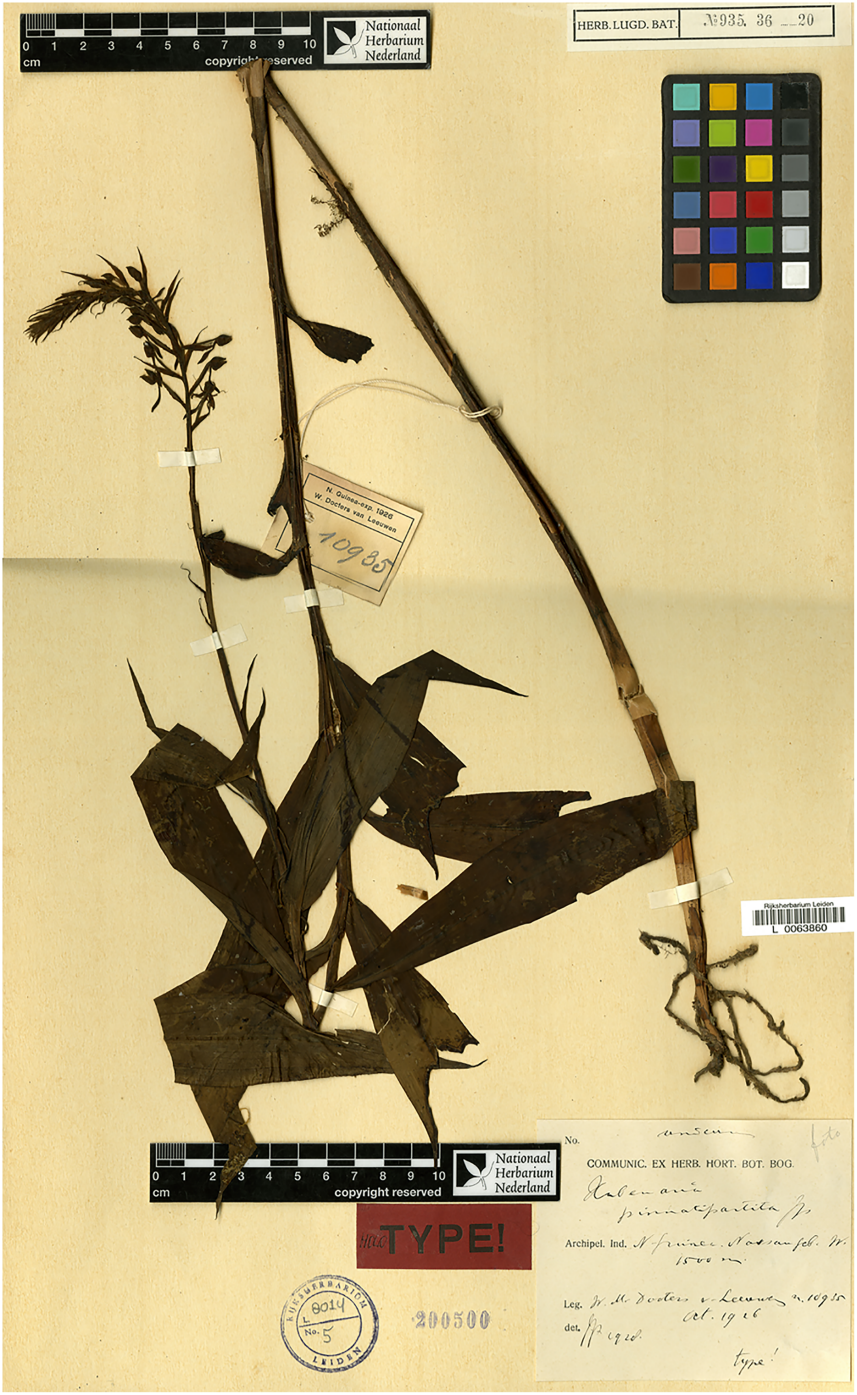
*Habenaria pinnatipartita* J. J. Sm., type specimen at Naturalis Biodiversity Center, L 0063860. (CC0 1.0; https://data.biodiversitydata.nl/naturalis/specimen/L%20%200063860).

**Figure 8:**
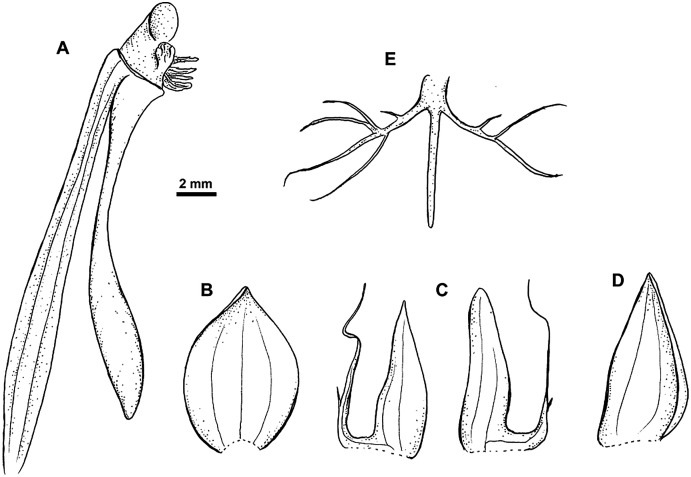
*Habenaria pinnatipartita* J. J. Sm.: (A) ovary with spur and gynostemium; (B) dorsal sepal; (C) petals; (D) lateral sepal; (E) lip (*van Leeuwen 10935*; L).

Plant up to about 80.0 cm tall, with tubular sheaths in the basal half, upper part leafy. Leaves about 10, distant, sessile, up to 19.5 cm long, 3.5 cm wide, lanceolate, acuminate, towards the base gradually narrowed, sheaths tubular. Inflorescence more than 25.0 cm long, many-flowered, peduncle with a few foliaceous scales in upper part. Floral bracts up to 27.0 mm long, ovate-oblong, gradually long caudate-acuminate. Pedicel with ovary 20.0 mm long, subrostrate-narrowed at the apex, 6-ribbed. Flowers green. Dorsal sepal up to 8.0 mm long, 5.0 mm wide, concave, ovate to elliptic-ovate, apex shortly obtusely contracted, 3-veined. Lateral sepals up to 10.0 mm long, 5.0 mm wide, reflexed, concave, obliquely ovate-triangular, narrowed towards the apex, acute, 3-veined. Petals deeply bilobed just above the base, and here about 3.0 mm wide, 2-veined; anterior lobe 10.0 mm long, obliquely broadly linear, long filiform-produced, along the front margin provided with a similar lobule which is more or less shorter and sometimes with an additional tooth-like lobule, 1-veined; posterior lobe shorter than anterior one, obliquely oblong-triangular, falcate, acute or subobtuse, 2-veined. Lip 3-lobed above the base, claw 2.0 mm long, quadrangular, flat with two inconspicuous longitudinal grooves; middle lobe up to 9.0 mm long, linear, obtuse, convex; lateral lobes 8.5–10.0 mm long, deflexed, obliquely linear, filiform-produced, outer margin frequently provided with 3 patent, unequal, filiform lobules and sometimes the front margin also with a very short lobule. Spur 16.0 mm long, somewhat incurved, clavate, subterete, obtuse, inflated part dorsally with an obtuse longitudinal rib. Gynostemium 3.5 mm long; anther channels somewhat falcate-incurved; stigmaphores porrect, surpassing the rostellum arms. Figs. 7–8.

*Habitat:* Terrestrial in lower montane forest (Schuiteman, Vermeulen & De Vogel, 2001–2010) at 1,500 m elevation.

*Distribution*: New Guinea.

*Representative specimen*: Indonesia. Prov. Papua. Nassau Mts. 1,500 m. Oct 1926. *W.M. Docters van Leeuwen 10935* (L!, RENZ!). Fig. 4.

*Notes: Habenaria pinnatipartita* can be distinguished from *H. paucipartita* based on the petal and lip shape. In the latter the anterior petal lobe is almost entire (*vs* with a single filiform segment), lip lateral lobes have a few subulate projections and short, irregular teeth (*vs* lateral lobes with 2–3 patent, unequal, filiform lobules and sometimes near the apex). The unique character of this species is the presence of a longitudinal rib on the dorsal side of the spur, not found elsewhere, In the field both species can be recognized also by the flower color—in *H. pinnatipartita* it is uniformly green, whereas in *H. paucipartita* – the lip is white, and the other flower segments are green.

The specimen of *Docters van Leeuwen 10935* deposited at RENZ is most likely a fragment of type material from L, although it is not labeled as such directly, and constitutes a type collection in accordance with Article 8.3 Ex. 8 of the ICN (Turland et al., 2018). We decided to designate a specimen in L that was undoubtedly examined by the author of *H. pinnatipartita* as a lectotype to avoid any future misunderstandings.

1.4. ***Habenaria polyschista*** Schltr., Fl. Schutzgeb. Südsee: 80. 1905. TYPE: New Guinea. *Schlechter 14475* (B†); Papua New Guinea (Deutsch Neu-Guinea). *T.M. Reeve 528* (Neotype, *designated here*: LAE!, Isoneotype: RENZ!).

= *Habenaria ramosa* R.S. Rogers & C.T. White, Trans. Roy. Soc. S. Austral 44: 117. 1920. TYPE: Papua New Guinea. *C.T. White 680* (AD, BRI).

**Figure 9:**
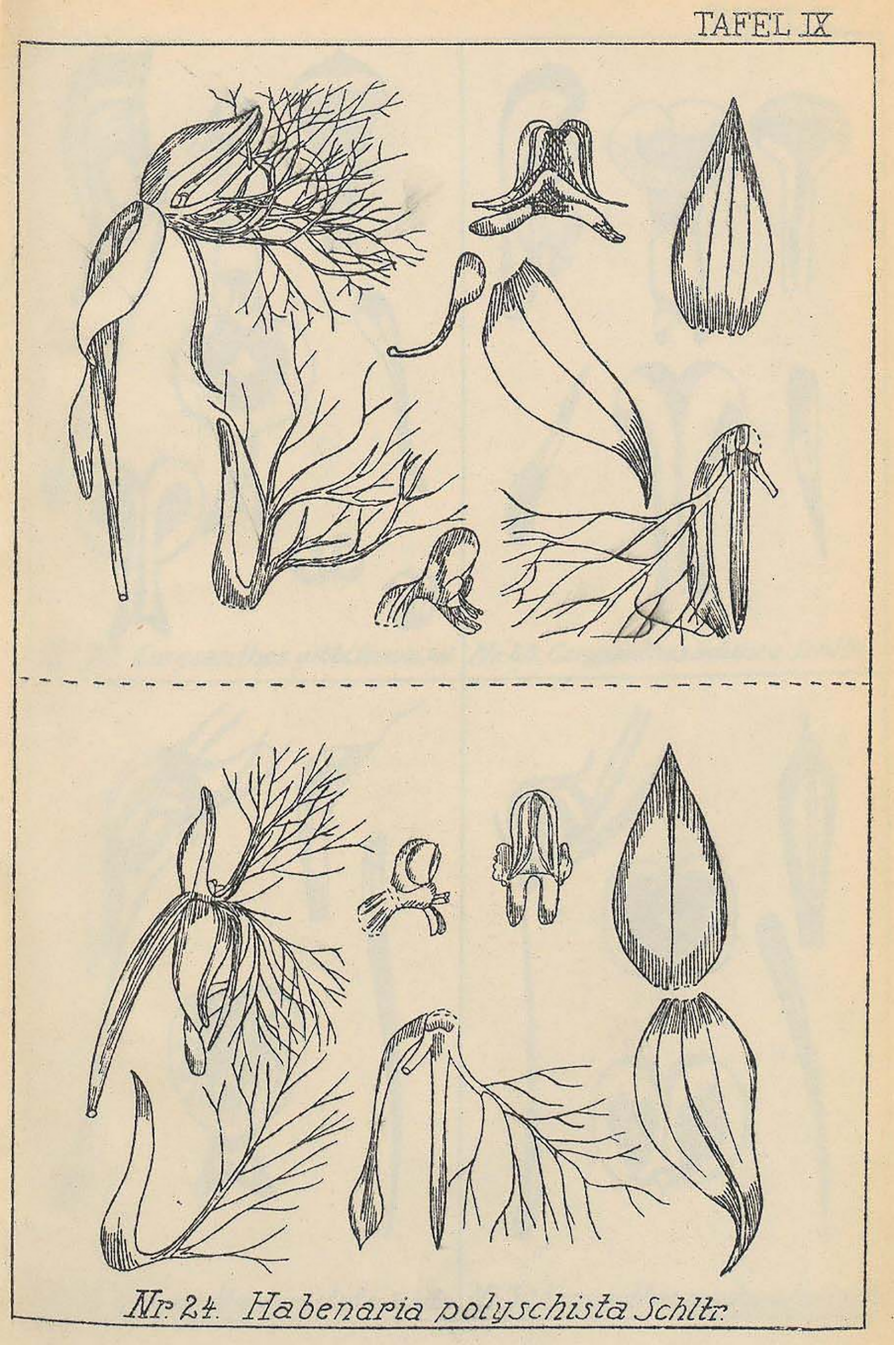
*Habenaria polyschista* Schltr., original Schlechter’s drawing from Figurenatlas zu den Orchidaceen von Deutsch-Neu-Guinea, Feddes Repert. Spec. Nov. Regni Veg. Beih. 21(1), 1923 (Plate IX, nr 24). URL: https://www.biodiversitylibrary.org/page/57688225.

Plant up to 70.0 cm tall, leafy from the base. Leaves erect-patent, up to 20.0 cm long, 3.0 cm wide, decreasing in size in the upper part, oblanceolate, acuminate. Inflorescences up to 20.0 cm long, densely many-flowered. Floral bracts ca 25.0 mm long, lanceolate, long-acuminate. Pedicel with ovary up to 25.0 mm long, clavate. Flowers green. Dorsal sepal up to 8.0 mm long, 4.0 mm wide, concave, ovate, acute, 3-veined. Lateral sepals 8.0 mm long, 3.5–4.0 mm wide, obliquely ovate-lanceolate, long-acuminate, 3-veined. Petals bilobed just above the base; anterior lobe longer than the posterior one, from a linear base divided into numerous, more or less divergent filiform segments, 1-veined; posterior lobe about 7.0–7.5 mm long, linear-subfalcate, long-acuminate, 3-veined. Lip 3-lobed just above the base; middle lobe 8.0 mm long, narrowly linear, subacute; lateral lobes from a linear base divided into numerous, filiform segments similar to those of the anterior lobe of the petals. Spur slightly shorter than the ovary, pendulous, narrowly cylindrical, slightly swollen in apical third, attenuate towards obtuse apex. Gynostemium 4.5 mm long, anther channels as long as the stigmaphores. Fig. 9.

*Habitat:* Terrestrial in primary rain forest and lower montane forest at the altitude of 750–1,400 m.

*Distribution*: New Guinea, Solomon Islands.

*Representative specimen*: Papua New Guinea. Torricelli Mts. Alt. 1,000 m. Apr 1902. *R. Schlechter 14475* (B†); Dilava. 14 Aug 1918. *C.T. White 680* (BRI); Mt. Bupuwoto, Tapini, Goilala Distr. Alt. 1,400 m. Dec 1980. *T.M. Reeve 528* (LAE!, RENZ!); Madang Distr., Kar Kar Island. Alt. 750 m. 16 Jan. 1968. *C. Ridsdale 33983* (LAE!, RENZ!); New Ireland. 10 Oct 1975. *M.J. Sands 1941* (K!); Solomon Isl. Malaita Is. Mt. Alasaa. Alt. 2000 ft. 21 Nov 1965. *E.J.H. Corner 3036* (AMES!, K!, RENZ!). Fig. 4.

*Notes: Habenaria polyschista* can be easily distinguished from other *Habenaria* species reported from New Guinea by having numerous filiform outgrowths along both margins of the lateral lobes of the lip as well as on the anterior petal lobe giving them a feather-like appearance.

Based on our best knowledge, a type collection for *H. polyschista* does not exist today. Therefore, for the sake of nomenclatural stability we decided to designate *Reeve 528* as a neotype and selected a specimen in the Papua New Guinea National Herbarium that is well preserved and corresponds to the species concept.

2. ***Habenaria elongata*-group**

Leaves either basal or cauline, petals undivided, unlobed, lip lateral lobes narrow, entire.

### Key to subgroups of *Habenaria elongata*-group

1. Leaves cauline 2.1. ***H. lamii*-subgroup**
1* Leaves basal, rosulate 2
2. Leaves narrow, linear to narrowly lanceolate, widest near the middle 2.2. ***H. elongata*-subgroup**
2* Leaves oblanceolate to obovate-elliptic, widest above towards apex 2.3. ***H. torricellensis*-subgroup**

2.1. ***Habenaria lamii*-subgroup**

Leaves above the stem base. Petals entire. Lip 3-lobed, lobes entire. Anther channels elongate, stigmaphores ligulate.

2.1.1. ***Habenaria lamii*** J. J. Sm., Nova Guinea, Bot. 14: 338. 1929. TYPE: Indonesia. *H.J. Lam 1743* (Lectotype, as holotype in Van Royen, 1979: BO!, Isolectotype: L!).

**Figure 10:**
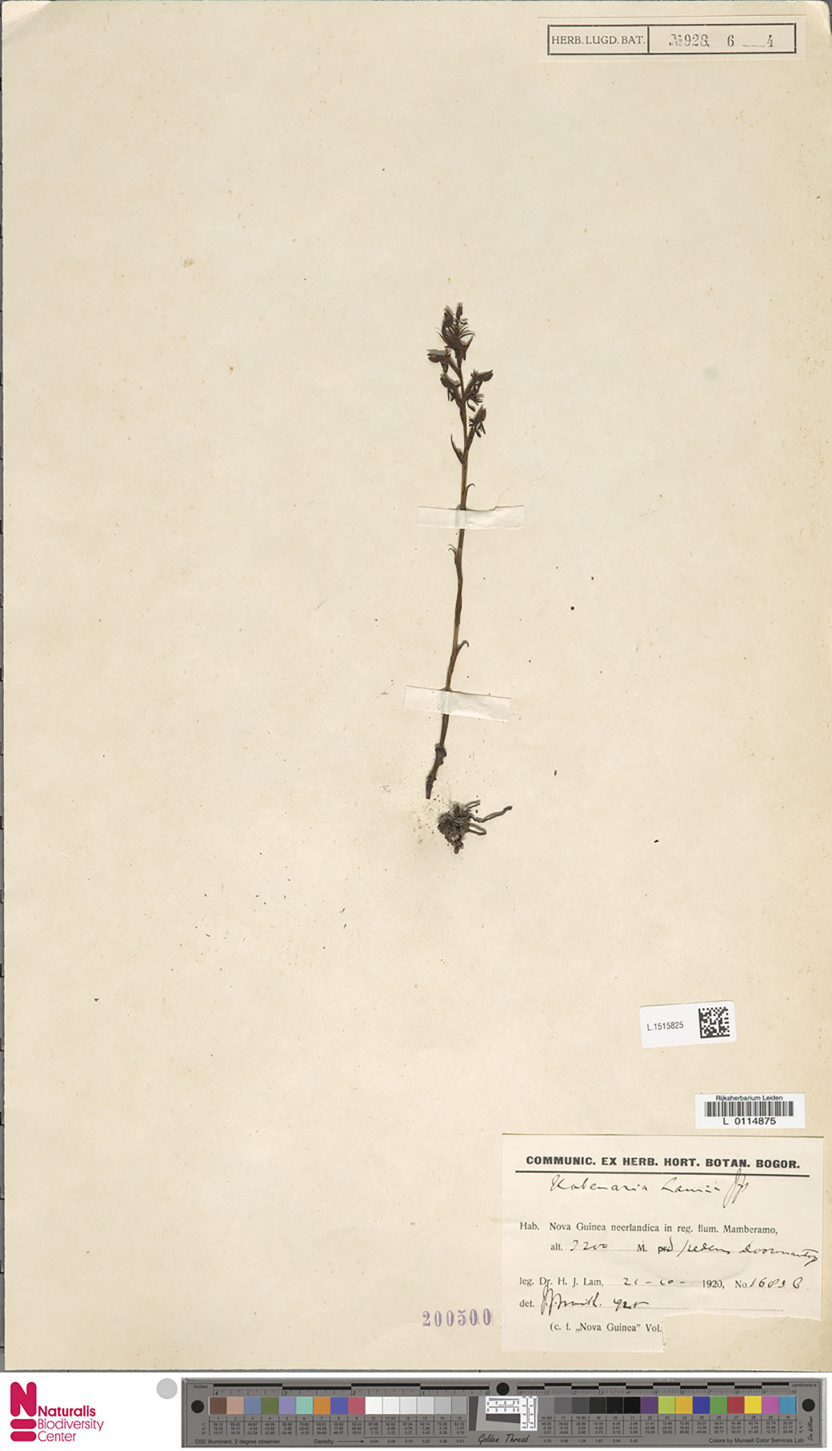
*Habenaria lamii* J. J. Sm., herbarium specimen at Naturalis Biodiversity Center, L.1515825. (CC0 1.0; https://data.biodiversitydata.nl/naturalis/specimen/L.1515825).

**Figure 11:**
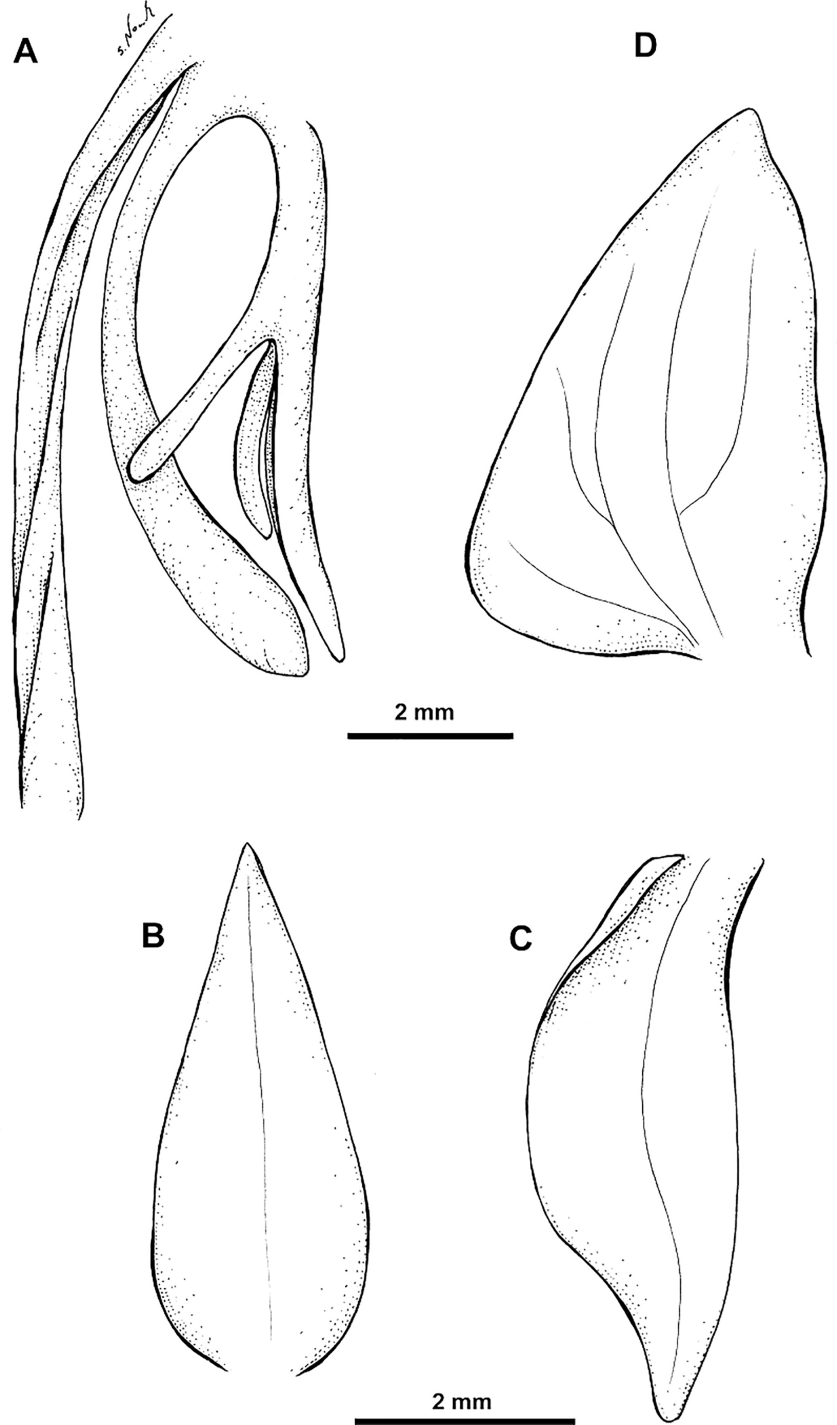
*Habenaria lamii* J. J. Sm., (A) ovary with lip and spur; (B) dorsal sepal; (C) petal; (D) lateral sepal (drawn from *Lam 1683b*; L).

Plant about 20.0–25.0 cm tall. Leaves about 3, above the base, spaced, small, 1.2–1.25 cm long, 0.6 cm wide, ovate, acuminate, sheaths tubular. Inflorescence erect, peduncle 12.0–14.0 cm long, scales 4, tubular, acuminate; rachis 6.0 cm long, laxly 8–10-flowered. Floral bracts 14.0 mm long, rhombic, acuminate. Pedicel with ovary 10.0–11.0 mm long, deeply 6-grooved. Flowers light green, outside suffused with dark brown, spur yellowish green. Dorsal sepal 4.5–4.8 mm long, 1.8–2.8 mm wide, concave, broadly ovate, obtuse, 1-veined. Lateral sepals 5.1–5.8 mm long, 2.2–3.8 mm wide, reflexed, obliquely ovate, somewhat falcate, obtuse, 1- or 3-veined. Petals 4.4–5 mm long, 3.1–3.8 mm wide, obliquely ovate-triangular, obtuse, primarily 3-veined. Lip 3-lobed, shortly clawed, with a transverse convex thickening near the base; middle lobe 4.0–5.0 mm long, ligulate, obtuse; lateral lobes about 3.0–3.5 mm long, linear-ligulate, obtuse, pendent. Spur 8.0–9.0 mm long, narrowly cylindrical, somewhat swollen at the blunt apex. Gynostemium about 2 mm long; anther channels and stigmaphores very short. Figs. 10–11.

*Habitat:* Terrestrial in montane grassland. Alt. 2,200–3,260 m.

*Distribution*: New Guinea.

**Figure 12:**
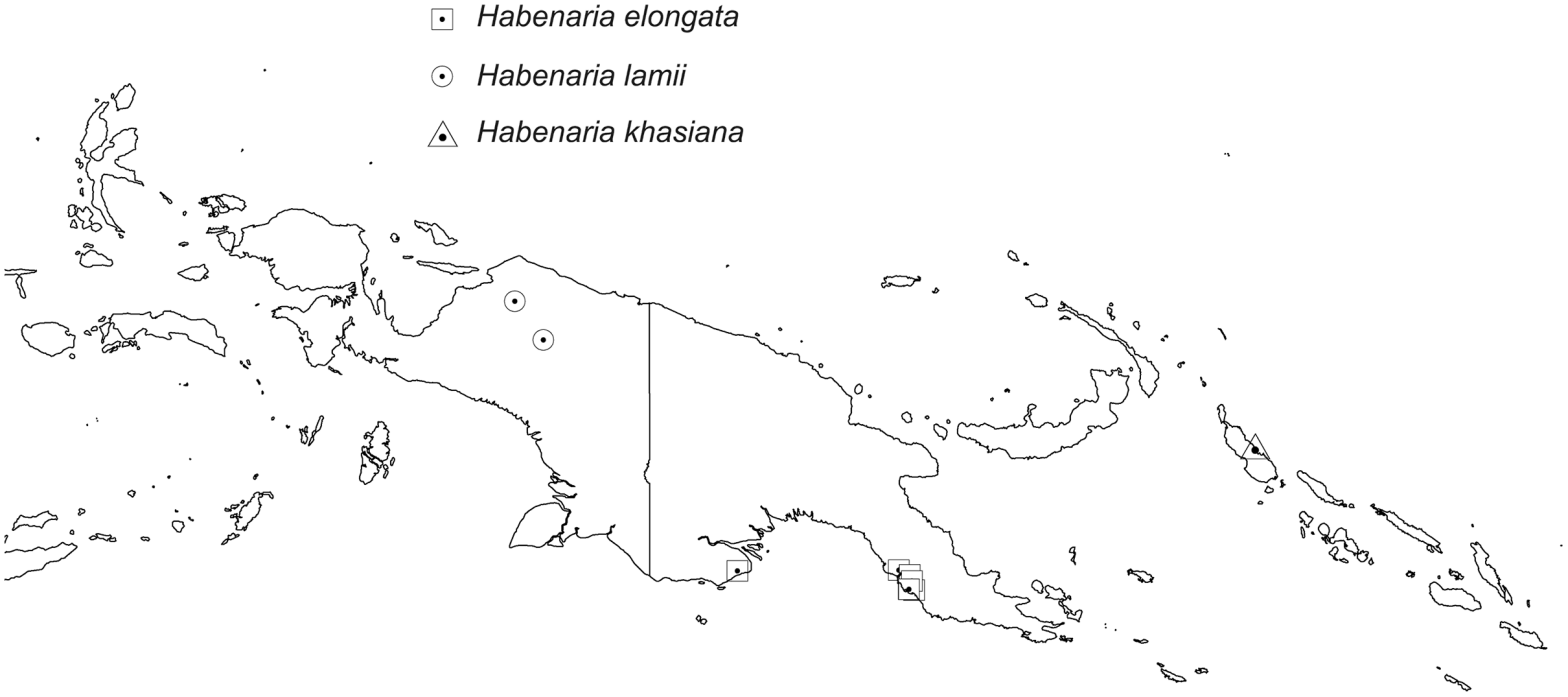
Distribution of *Habenaria elongata, H. lamii* and *H. khasiana*.. Base map downloaded from www.naturalearthdata.com.

*Notes:* This species can be easily separated from other species occurring in New Guinea by the combination of the following characters: cauline leaves, entire petals and 3-lobed lip with entire lobes.

*Representative specimen*: Indonesia. Prov. Papua. Doorman Top. Alt. 3260 m. 24 Oct 1920. *H.J. Lam 1743* (BO!, L!); Doormantop. Alt. 2200 m. *H.J. Lam 1683b* (L!). Fig. 12.

Van Royen (1979) identified *Lam 1743* (BO) collection as a holotype of *Habenaria lamii*, which should be considered as a designation of the lectotype according to the Art. 9.10 of the ICN (Turland et al., 2018).

2.2. ***Habenaria elongata*-subgroup**

Leaves basal, narrow, linear to narrowly lanceolate. Petals undivided. Lip 3-lobed, lobes entire. Anther almost orbicular, antherophores shorter than stigmaphores, auricles stalked, prominent

### Key to species of *Habenaria elongata-*subgroup

1. Spur ca twice longer than pedicellate ovary 2
1* Spur subequal to or shorter than pedicellate ovary 3
2. Spur free from the ovary 2.2.1. ***H. elongata***
2* Spur connate basally with ovary apex 2.2.2. ***H. elongata* var. *leptophylla***
3. Lip middle lobe very narrowly linear, lateral lobes upcurved, filiform 2.2.3. ***H. khasiana***
3* Lip middle lobe ligulate-linear, lateral lobes erect, linear to lanceolate 4
4. Leaves 1–1.2 cm wide, floral bracts up to 20 mm long, longer than pedicellate ovary, lip lateral lobes shorter and much narrower than the middle one, spur up to 6 mm long 2.2.5. ***H. rumphii***
4* Leaves 0.4–0.8 cm wide, floral bracts up to 8 mm long, shorter than pedicellate ovary, lip lateral lobes slightly longer than the middle one, spur 11–13 mm long 2.2.4. ***H. ochroleuca***

2.2.1. ***Habenaria elongata*** R. Br., Prodr. Fl. Nov. Holland.: 313. 1810. TYPE: Australia. *R. Brown 5540* (Lectotype, designated by Clements, 1989: BM!—photo seen, Isolectotypes: E!—photo seen, K!); *R. Brown s.n*. (Syntypes: AMES!, BM!, BR!, K-L!); *R. Brown s.n*. (Syntypes: E!, K!); *R. Brown s.n*. (Syntype: BM!). ≡ *Pecteilis elongata* (R.Br.) M. A. Clem. & D. L. Jones, Austral. Orchid Rev. 83(6): 51. 2018, *syn. nov*.

*= Habenaria triplonema* Schltr., Repert. Spec. Nov. Regni Veg. 9: 435. 1911. TYPE: Australia. *M. Holtze 979* (B†; MEL), *syn. nov*. ≡ *Pecteilis triplonema* (Schltr.) M.A.Clem. & D.L.Jones, Austral. Orchid Rev. 83(6): 51. 2018, *syn. nov*.

**Figure 13:**
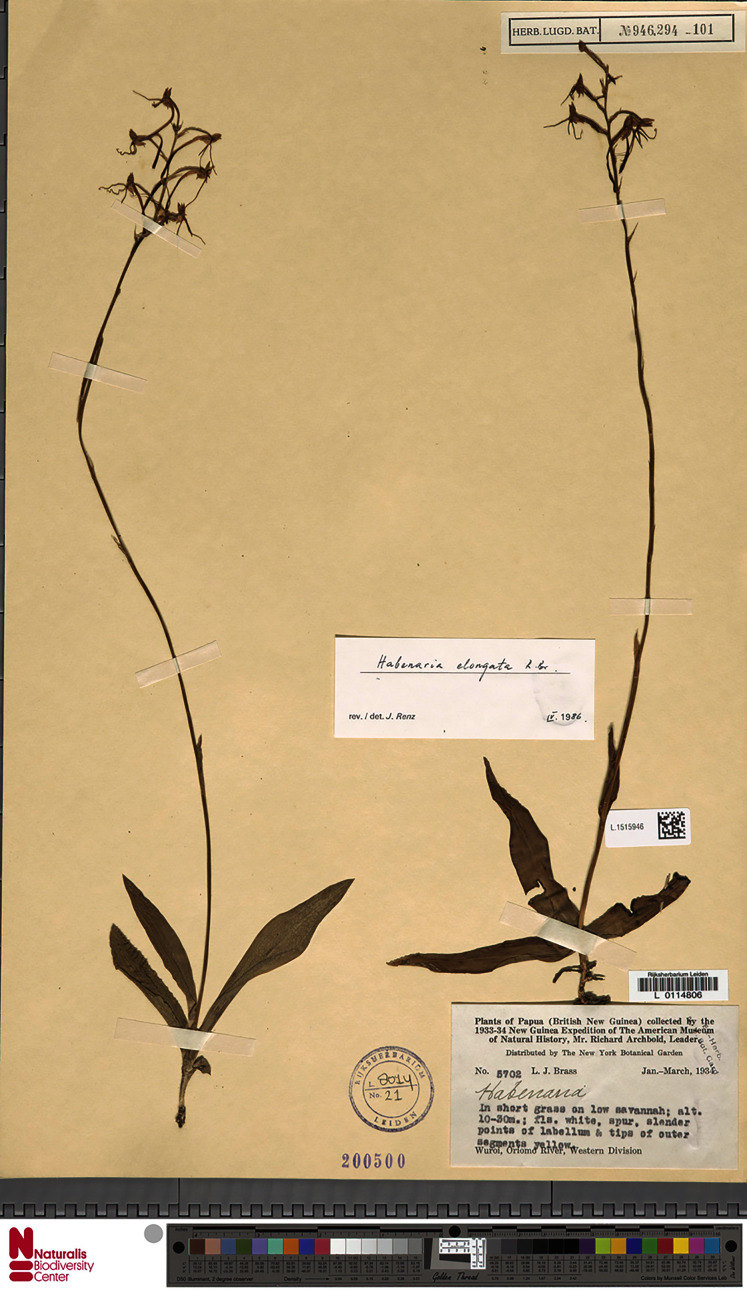
*Habenaria elongata* R. Br., herbarium specimen at Naturalis Biodiversity Center, L.1515946. (CC0 1.0; https://data.biodiversitydata.nl/naturalis/specimen/L.1515946).

**Figure 14:**
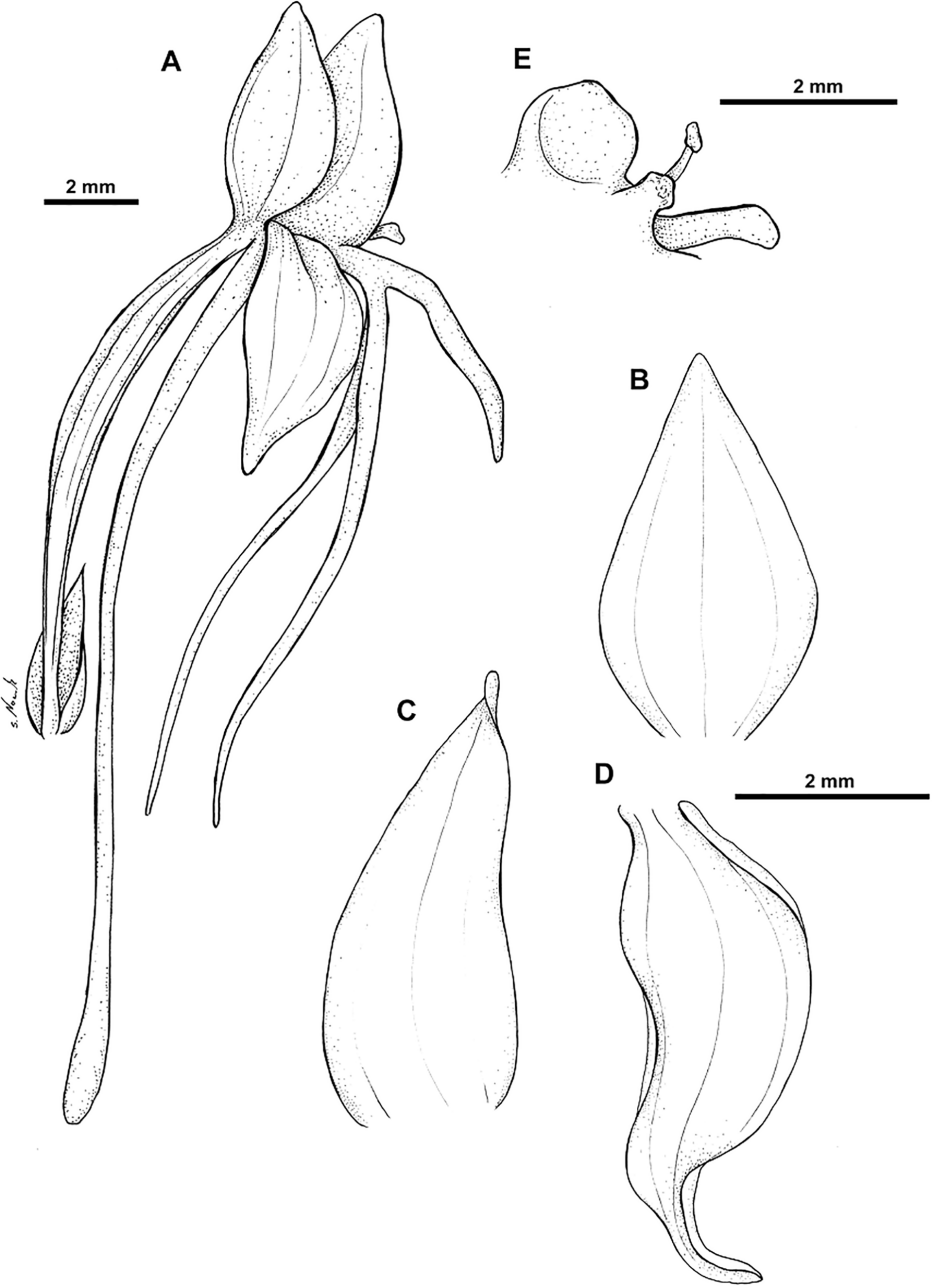
*Habenaria elongata* R. Br.: (A) flower; (B) dorsal sepal; (C) petal; (D) lateral sepal; (E) gynostemium (drawn from *Brass 5702;* (A) & (E)—AMES, (B)–(D)—BO).

Plant 30.0–60.0 cm tall, leafy at the base. Leaves 2–5, erect-patent, 5.0–12.0 cm long, 0.6–2.0 cm wide, oblong to elliptic, narrowly ovate to obovate, acute or apiculate. Inflorescence with 6–10 distant, bract-like scales; rachis 4.5–16.0 cm long, rather laxly 8–20-flowered. Floral bracts 5.0–7.0 mm long, ovate-lanceolate, acuminate. Pedicel with ovary 13.0–14.0 mm long, narrowly cylindrical. Dorsal sepal 4.0–6.0 mm long, 3.0–4.0 mm wide, concave, broadly ovate to elliptic-ovate, apex obtuse to subacute, 3-veined. Lateral sepals 5.0–7.0 mm long, 2.5–4.0 mm wide, deflexed, obliquely lanceolate-ovate to triangular-ovate, subacute, 3-veined. Petals entire, 5.0–7.0 mm long, 2.5–3.0 mm wide, obliquely ovate-lanceolate to narrowly triangular-ovate, often with a small tooth in front near the base, apex obtuse, 2-veined. Lip 3-lobed above claw 1.0 mm long; middle lobe 5.0–8.0 mm long, 0.5 mm wide, narrowly linear, subobtuse; lateral lobes (7)15.0–25.0 mm long, 0.4 mm wide, narrowly linear in the lower half, filiform above. Spur 23.0–35.0 mm long, narrowly cylindrical, basally pendulous becoming strongly incurved inwards, slightly swollen in apical fourth. Gynostemium 1.7–2.0 mm long, stigmaphores 3.0 mm long, subulate, anther channels ca. 1.0 mm long. Figs. 13–14.

*Habitat:* Terrestrial in short grass on low savanna. Alt. 10–30 m.

*Distribution*: Northern Australia, New Guinea.

*Representative specimen*: Papua New Guinea. Western Prov. Oriomo River, Wuroi. Alt. 10–30 m. *L.J. Brass 5702* (AMES!, BO!, BRL, K!, L!, NY!, RENZ!, US!). Fig. 12.

*Notes: Habenaria elongata* is very similar to *H. elongata* var. *leptophylla*, but its spur is completely free from the ovary.

Dockrill (1969) considered *H. triplonema* and *H. elongata* as separated species based on the length of the lip lateral lobes—*H. triplonema* was in his opinion distinguished by equally long lobes while in *H. elongata* lobes are unequal. However, this character is not consistent in type specimens of *H. elongata* and also Brown (1810) in his very short description of *H. elongata* did not mention inequality of the lateral lip segments. A substantial inequality of the lateral lobes was not observed so far in any *Habenaria* representative therefore we think it is only artefact.

Unfortunately, the type specimen of *H. triplonema* (*Holtze 979*) is apparently missing and the type material of *H. elongata* (*Brown 5540*, K) does not enable us to estimate infraspecific variation of this taxon. The original diagnosis of *H. elongata* is very short and without mention of the unequal length of the lateral lip lobes (Brown, 1810). The only known material of *H. elongata* other than the type is *Brass 5702*, in which we did not observe this character. We had the opportunity to examine *H. triplonema* only based on the illustration and description provided by Dockrill (1969).

2.2.2. ***Habenaria elongata* var. *leptophylla*** Renz, Pl. Syst. Evol. 155(14): 330. 1987. TYPE: Papua New Guinea. *J. Renz 11897* (Holotype: RENZ!). ≡ *Habenaria leptophylla* (Renz) D. L. Jones, Orchadian 13(11): 520. 2002. ≡ *Pecteilis leptophylla* (Renz) M. A. Clem. & D. L. Jones, Austral. Orchid Rev. 83(6): 52. 2018, *syn. nov*.

**Figure 15:**
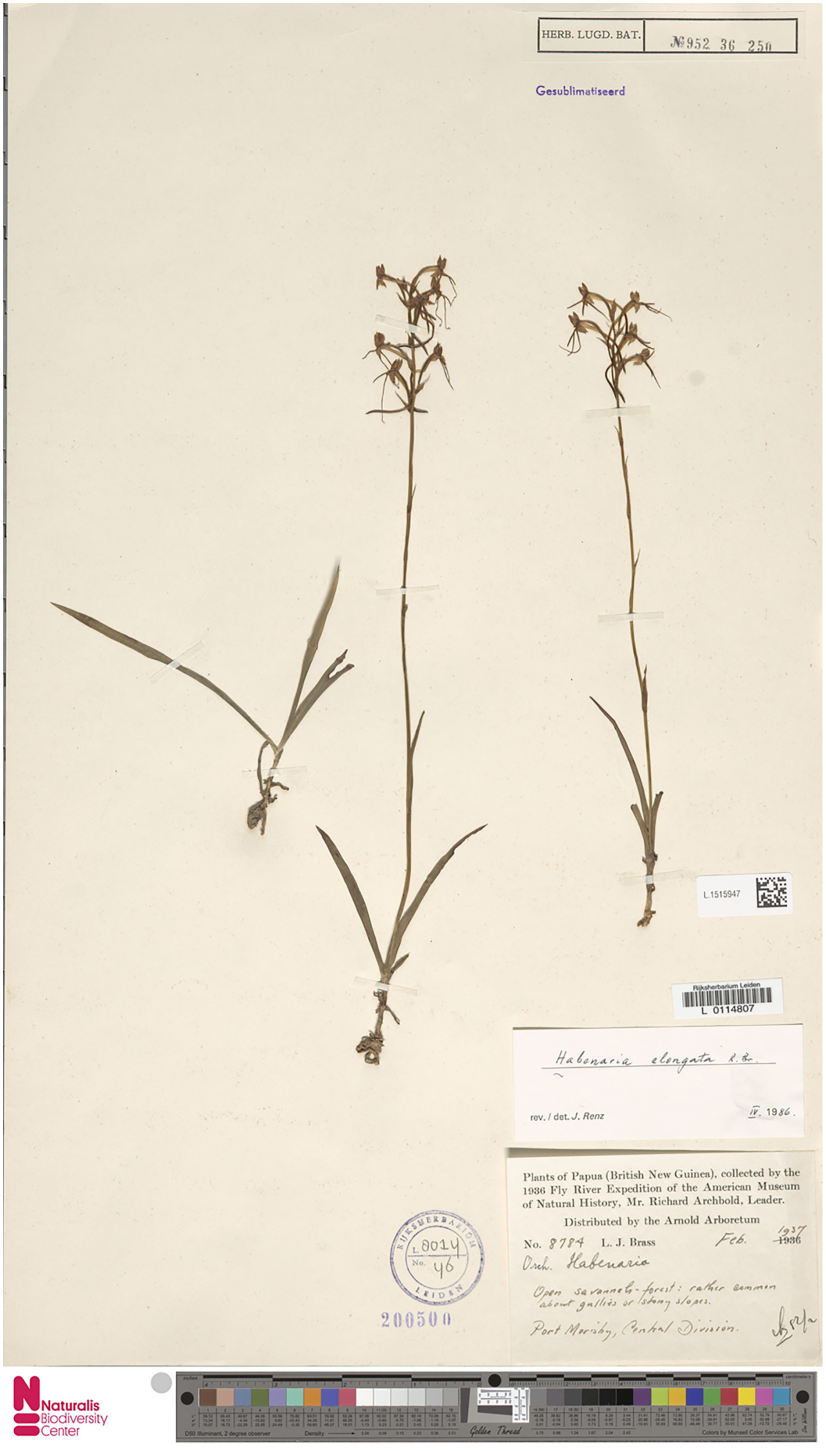
*Habenaria elongata* var. *leptophylla* Renz, herbarium specimen at Naturalis Biodiversity Center, L.1515947. (CC0 1.0; https://data.biodiversitydata.nl/naturalis/specimen/L.1515947).

**Figure 16:**
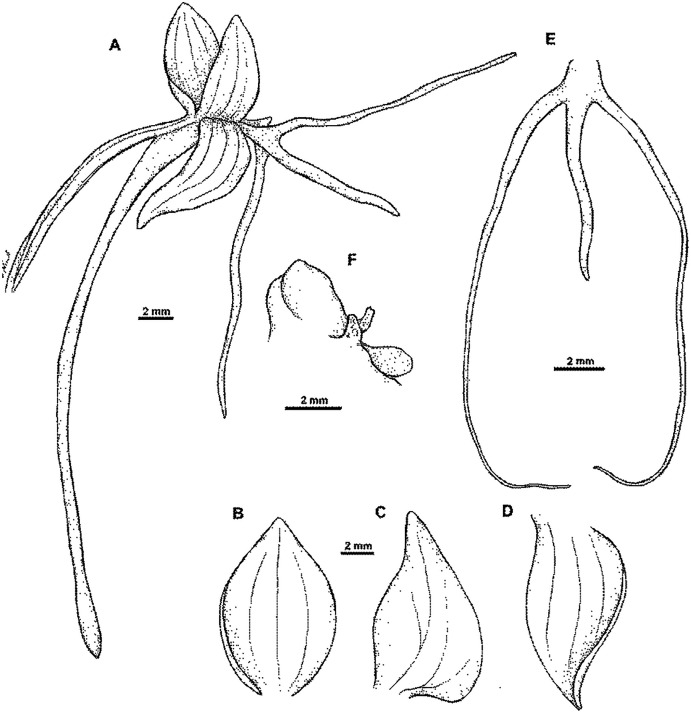
*Habenaria elongata* var. *leptophylla* Renz: (A) flower; (B) dorsal sepal; (C) petal; (D) lateral sepal; (E) lip; (F) gynostemium (drawn from *Brass 8784;* (A) & (F)—AMES, (B)–(D)—L).

Plant up to 45.0 cm tall, leafy at the base, with several bract-like leaves above. Leaves 2–4, up to 13.0 cm long, 0.3–0.8 cm wide, linear, grass-like, acute. Inflorescence 8.0 cm long, laxly several-flowered. Floral bracts 7.0 mm long, ovate, acute. Pedicel with ovary 15.0–16.0 mm long, fusiform. Flowers small, sepals pale yellow with white base, petals white, tipped pale yellow, lip base white, lobes bright citron, spur yellow-green with white base. Dorsal sepal 4.0–4.8 mm long, 2.7–3.2 mm wide, concave, ovate-elliptic, subobtuse, 3-veined. Lateral sepals 4.5–5.3 mm long, 2.5 mm wide, obliquely lanceolate-ovate, subobtuse to subacute, 3-veined. Petals 3.8–5.0 mm long, 2.5–3.0 mm wide, obliquely elliptic-ovate, obtuse, primarily 2-veined, veins dichotomous, or 3-veined. Lip 3-lobed above short claw; middle lobe 6.0–7.0 mm long, 0.4 mm wide, linear, obtuse; lateral lobes 14.0–19.0 mm long, linear-filiform, subobtuse. Spur 29.0–33.0 mm long, filiform, very slightly swollen at the subobtuse apex, basally connate with the ovary for ca 1 mm. Gynostemium 2.5–3.0 mm long; stigmaphores more than twice longer than anther channels. Figs. 15–16.

*Habitat:* In open savanna-forest, damp places in open savanna-land. Alt. 20–60 m.

*Distribution*: New Guinea.

*Representative specimens*: Papua New Guinea. Central Prov. Waigani, in pratis udis. Alt. ca. 50 m. 2 Mar 1978. *J. Renz 11877* (RENZ!); Port Moresby, open savannah-forest. Feb 1937. *L.J. Brass 8784* (AMES!, L!); Kanosia, damp places in open savannah-land. Alt. ca. 20 m. 6 Feb 1935. *C.E. Carr 10005* (AMES!, BM!, CANB, K!, LAE!, L!, NY!, RENZ!); Central Distr., Vanapa subdistr. Rubologo, logging area. Alt. 100 ft. 7 Jan 1972. *M. Kumul NGF 36305* (K!, LAE!); Central District. One mile east of Brown river station along Hohoro Road. Alt. 60 m. 30 Dec 1975. *B. Verdcourt & al. 5809* (K!, UPNG!); Central District. Waigani near Insectory. 18 Feb 1969. *M. Pulsford 24* (UPNG!). Fig. 12.

*Notes:* This variety is very similar to *Habenaria elongata var. elongata*, from which it can be distinguished by the prominent fusion between the basal part of the spur and the apical part of the ovary, characters not found in any other *Habenaria* species reported from New Guinea. Otherwise, both taxa are alike.

2.2.3. ***Habenaria khasiana*** Hook.f., Fl. Brit. Ind. 6: 151.1890. ≡ *Habenaria graminea* Lindl., Gen. Sp. Orchid. Pl.: 318. 1835, *nom. illeg*. TYPE: India. *Wallich Catalogue no. 7041* (Lectotype, *designated here*: K-L!, Isolectotype: K!).

**Figure 17:**
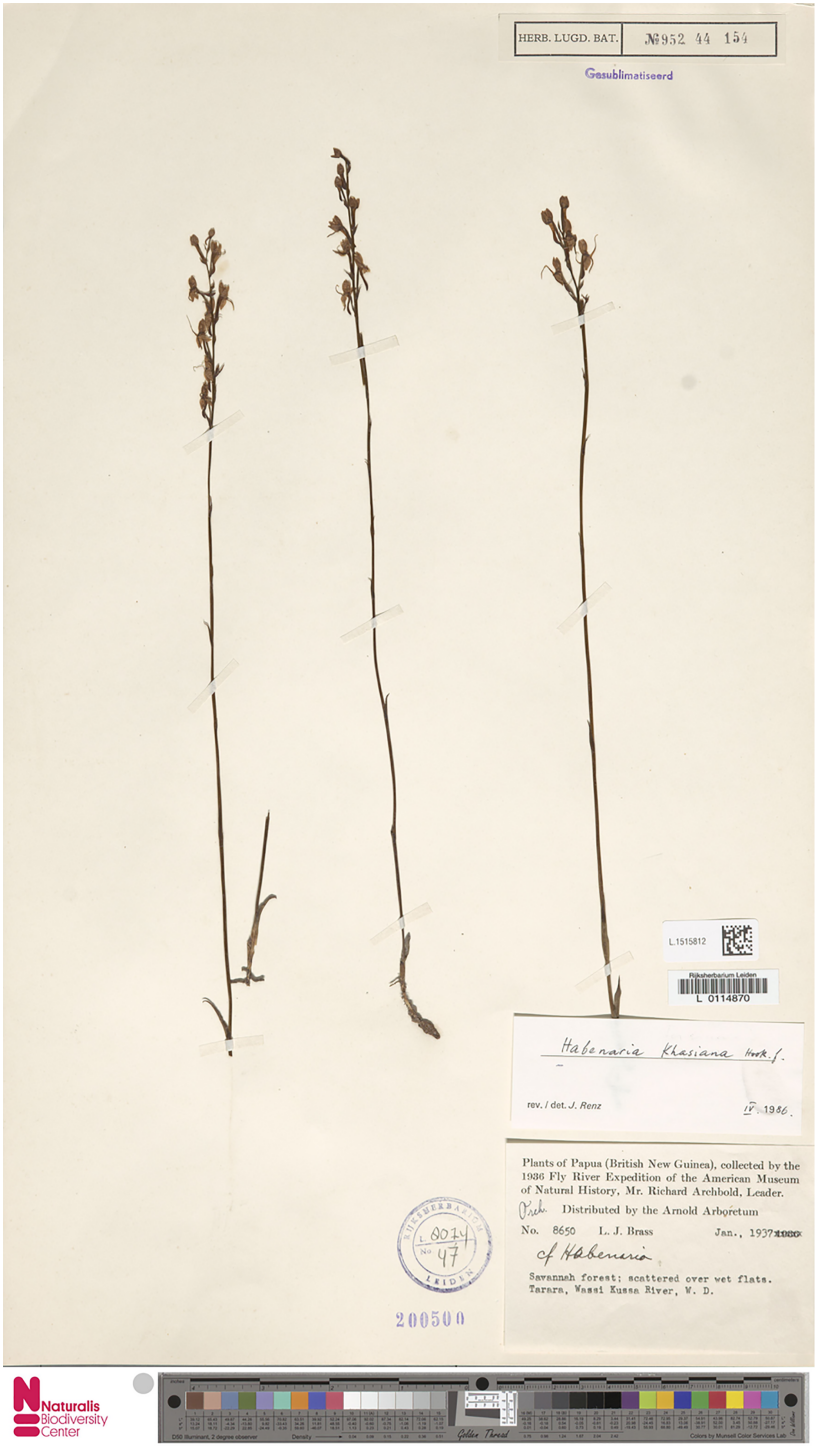
*Habenaria khasiana* Hook. f., herbarium specimen at Naturalis Biodiversity Center, L.1515812. (CC0 1.0; https://data.biodiversitydata.nl/naturalis/specimen/L.1515812).

**Figure 18:**
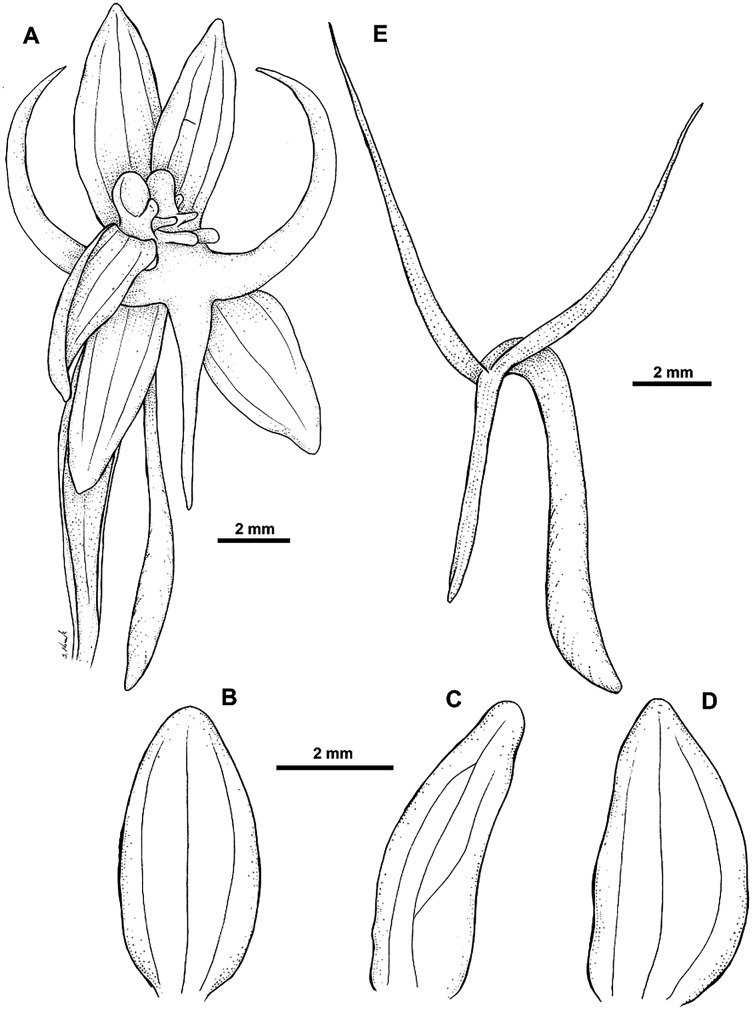
*Habenaria khasiana* Hook. f.: (A) flower; (B) dorsal sepal; (C) petal; (D) lateral sepal; (E) lip (drawn from *Brass 8650*; (A) AMES, (B)–(E)—L).

Plant up to about 35.0 cm tall, leafy at the base, with several scale-like leaves above. Leaves 1–3, up to 5.0 cm long, usually less than 0.5 cm wide, linear to linear-lanceolate, acute to acuminate. Inflorescence with few scales on the peduncle, rachis up to about 10.0 cm long, laxly few-flowered. Floral bracts up to 5.0 mm long, ovate-lanceolate, acute. Pedicel with ovary 12.0–17.0 mm long, narrowly cylindrical. Flowers greenish. Dorsal sepal 5.0–12.0 mm long, 2.0–3.0 mm wide, concave, ovate to ligulate-ovate, obtuse, 3-veined. Lateral sepals 5.0–12.0 mm long, 2.1–3.0 mm wide, obliquely ovate to oblong-ovate, obtuse, 3-veined. Petals 5.0–12.0 mm long, 1.5–2.2 mm wide, oblong-ovate to ligulate, obtuse, somewhat oblique, 1- or 2-veined. Lip 3-lobed above short claw; middle lobe 5.5–6.0 mm long, 0.5 mm wide, very narrowly linear, acute; lateral lobes 7.5–8.0 mm long, 0.7 mm wide, linear-filiform, acuminate, upcurved. Spur 8.0–17.0 mm long, slightly incurved, narrowly cylindrical, swollen in the apical third, blunt. Gynostemium 2 mm long; stigmaphores longer than anther channels. Figs. 17–18.

*Habitat:* Terrestrial in savanna; scattered over wet flats.

*Distribution*: India (Khasia), Laos, Cambodia, Thailand, New Guinea.

*Representative specimen*: Papua New Guinea. Western Prov. Tarara, Wassi Kussa River. *L.J. Brass 8650* (AMES!, BO!, BRI, L!, RENZ!). Fig. 12.

*Notes: Habenaria khasiana* appears to be similar to *H. rumphii* as both share similar habit and general flower morphology. In both species we can observe a similar lip shape, especially upcurved lateral lobes. Leaves of *H. khasiana* are short (up to 5 cm *vs* up to 17 cm), the inflorescence is laxly few-flowered (*vs* densely many-flowered), flowers can be larger, the lip middle lobe shorter than the lateral lobes (*vs* middle lobe longer than laterals) and a distinctly longer spur (8–17 mm long *vs* 1–6 mm). The flowers of *H. khasiana* are uniformly green, whereas the color range of *H. rumphii* flowers is much wider—from white, through dull green to purple.

*H. khasiana* can easily be distinguished from *H. lamii* by the upcurved lip lateral lobes (*vs* pendent), much shorter floral bract reaching up to 5 mm, shorter than pedicellate ovary (*vs* 14 mm long, longer than pedicellate ovary), and larger flowers.

2.2.4. ***Habenaria ochroleuca*** R. Br., Prodr. Fl. Nov. Holl.: 313. 1810. TYPE: Australia. *R. Brown 5539* (Lectotype, designated by Clements, 1989: BM!, Isolectotype: K!); *R. Brown s.n*. (Syntypes: BM!, K!); *R. Brown s.n*. (Syntype: K-L!). ≡ *Pecteilis ochroleuca* (R.Br.) M. A. Clem. & D. L. Jones, Austral. Orchid Rev. 83(6): 51. 2018, *syn. nov*.

**Figure 19:**
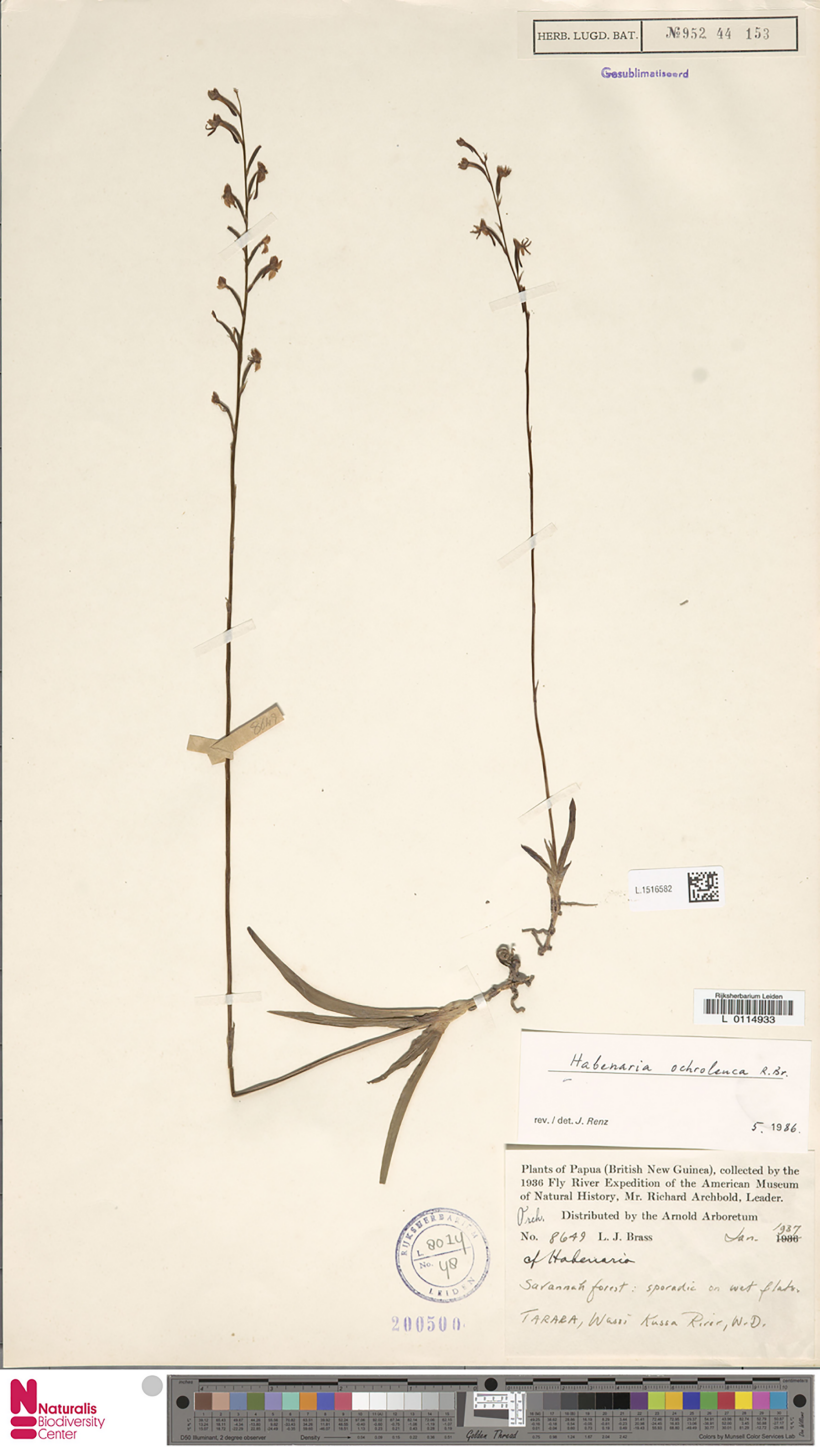
*Habenaria ochroleuca* R. Br., herbarium specimen at Naturalis Biodiversity Center, L.1516582. (CC0 1.0; https://data.biodiversitydata.nl/naturalis/specimen/L.1516582).

**Figure 20:**
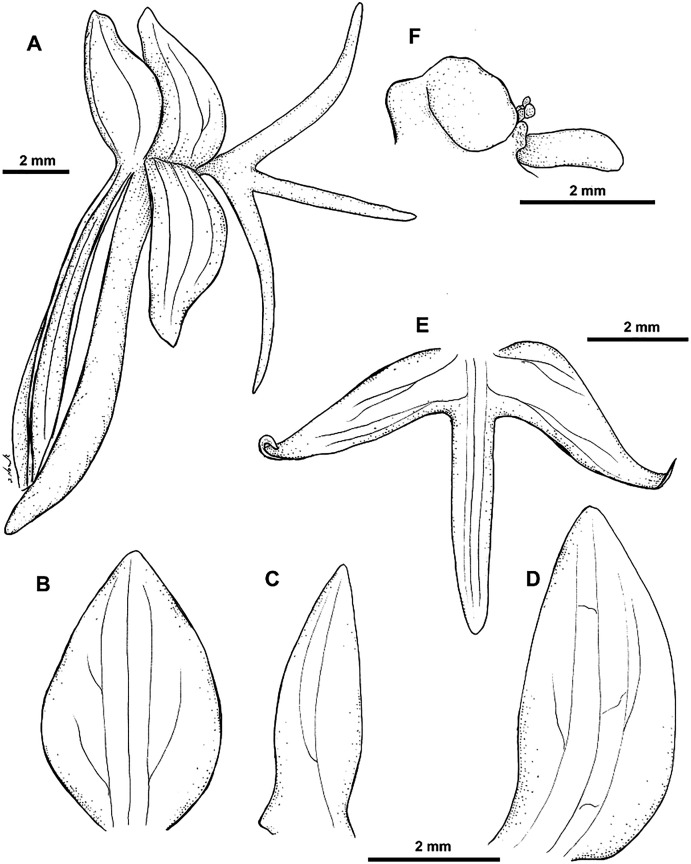
*Habenaria ochroleuca* R. Br.: (A) flower; (B) dorsal sepal; (C) petal; (D) lateral sepal; (E) lip; (F) gynostemium (drawn from *Brass 8663*; (A) & (F)—AMES; *Brown 5539*;(B)–(E)—BM).

Plant up to 50.0 cm tall, basally 2-4-leaved. Leaves 5.0–10.0 cm long, 0.4–0.8 cm wide, narrowly oblong, acute to acuminate. Inflorescence up to 16.0 cm long, laxly to densely few- to many-flowered, scales on peduncle subulate. Floral bracts up to 8.0 mm long, ovate-lanceolate, acute. Pedicel with ovary 10.0–14.0 mm long, narrowly cylindrical, attenuate towards apex, basally slightly swollen. Flowers white. Dorsal sepal 3.5–4.0 mm long, 2.5–2.8 mm wide, concave, broadly ovate, obtuse, 3-veined. Lateral sepals 5.0–6.0 mm long, 1.5–2.6 mm wide, obliquely oblong ovate, subobtuse, 3-veined. Petals 4.3–5.5 mm long, 2.0–2.6 mm wide, obliquely ovate-triangular, often at the base on the posterior margin with a very short lobule, obliquely truncate at the apex, 2-veined. Lip 3-lobed above ca 1.0 mm long claw; middle lobe 5.0–5.5 mm long, 0.6 mm wide, narrowly ligulate to linear, acute to subobtuse; lateral lobes 6.0–7.0 mm long, 0.7 mm wide, widely spread, linear-lanceolate, acuminate. Spur 11.0–13.0 mm long, slightly incurved, cylindrical, narrowed in the middle, attenuate towards subobtuse apex. Gynostemium 3.0 mm long, connective apically enlarged, hence hunchbacked; stigmaphores about 0.2 cm long, anther channels about half as long. Figs. 19–20.

*Habitat:* Terrestrial in grassland in savanna woodland in seasonally wet places.

*Distribution*: Norther Australia, New Guinea.

*Representative specimen*: Papua New Guinea. Western Prov. Tarara, Wassi Kussa River, savanna-forest, sporadic on wet flats. Jan 1937. *L.J. Brass 8649* (AMES!, BM!, BO!, BRI, L!, RENZ!); Tarara, Wassi Kussa River. Dec 1836. *L.J. Brass 8397* (AMES!); Tarara, Wassi Kussa River. Jan 1937. *L.J. Brass 8663* (AMES!); Western Division, Oriomo River, Dagwa. Alt. 45 m. Feb-Mar 1934. *L.J. Brass 6035* (NY!). Fig. 20.

*Other specimen examined:* Australia. *R. Brown 5539* (BM!, K!).

*Notes: Habenaria ochroleuca* is similar in habit and position of the lateral lobes of the lip to both *H. rumphii* and *H. khasiana*. In *H. khasiana* and *H. ochroleuca* lip middle lobe is shorter than the lateral lobes, but *H. khasiana* has shorter leaves (up to 5 cm *vs* 5–10 cm). Leaves of *H. ochroleuca* are narrower than in *H. rumphii* (0.4–0.8 cm *vs* 1–1.2 cm), the floral bracts shorter (up to 8 mm *vs* up to 20 mm), and the spur longer (11–13 mm *vs* up to 6 mm).

2.2.5. ***Habenaria rumphii*** (Brongn.) Lindl., Gen. Sp. Orchid. Pl.: 320. 1835. ≡ *Platanthera rumphii* Brongn., Voy. Monde: 194. I. 38 a. 1834. TYPE: Indonesia. *J.S.C. Dumont d’Urville s.n*. (P!). ≡ *Pecteilis rumphii* (Brongn.) M.A.Clem. & D.L.Jones, Austral. Orchid Rev. 83(6): 51. 2018, *nom. inval*. ≡ *Pecteilis rumphii* (Brongn.) M.A.Clem. & D.L.Jones, Austral. Orchid Rev. 84(1): 19. 2019, *syn. nov*.

= *Habenaria stauroglossa* Kraenzl., Fl. Kaiser Wilh. Land: 35. 1889. TYPE: Papua New Guinea. *M.U. Hollrung 319* (B†).

= *Habenaria dahliana* Kraenzl., Fl. Neu Pommern: 106. 1898. TYPE: Papua New Guinea. *F.O. Dahl s.n*. (B†). ≡ *Satyrium dahlianum* (Kraenzl.) Kuntze, Deutsche Bot. Monatsschr. 21: 173. 1903.

= *Habenaria rumphii* var. *meraukensis* J.J. Sm., Bull. Dép. Agric. Indes Néerl. 22: 6. 1909. TYPE: Indonesia. *B. Branderhorst 301* (Syntypes: BO).

**Figure 21:**
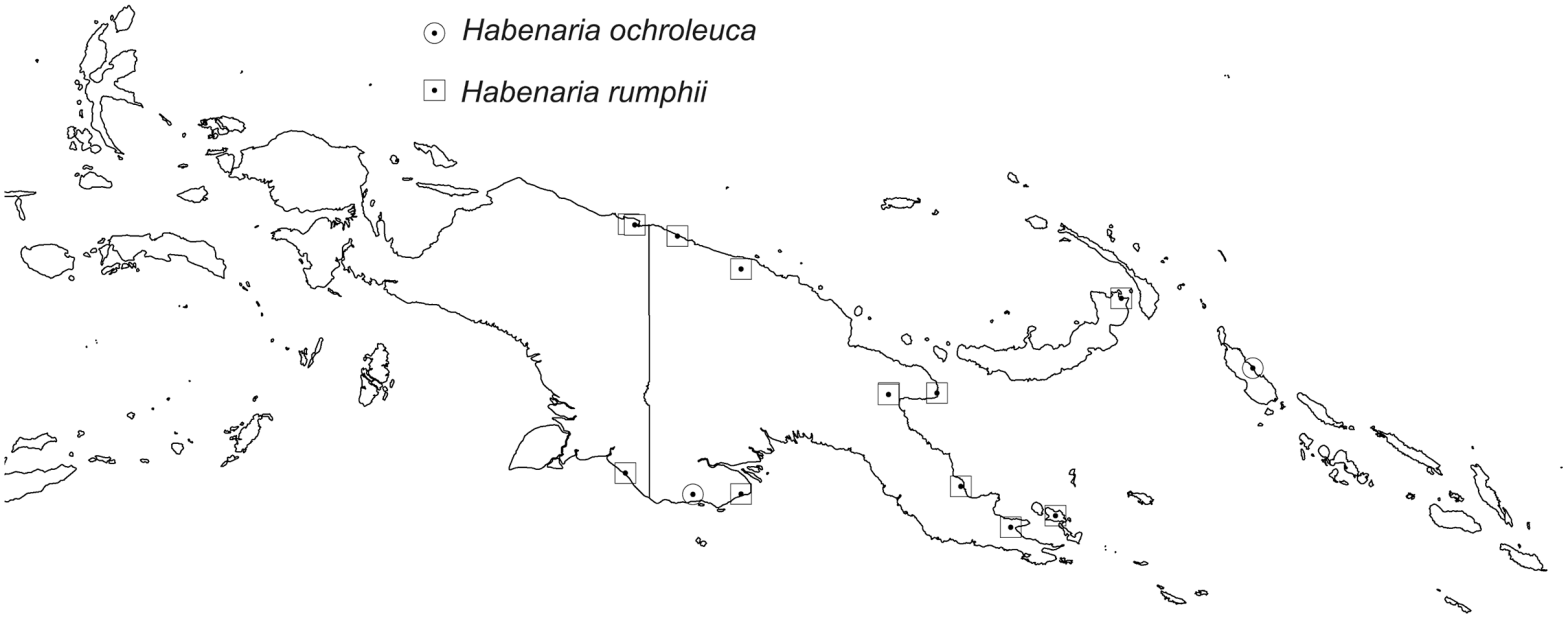
Distribution of *Habenaria ochroleuca* and *H. rumphii*. Base map downloaded from www.naturalearthdata.com.

**Figure 22:**
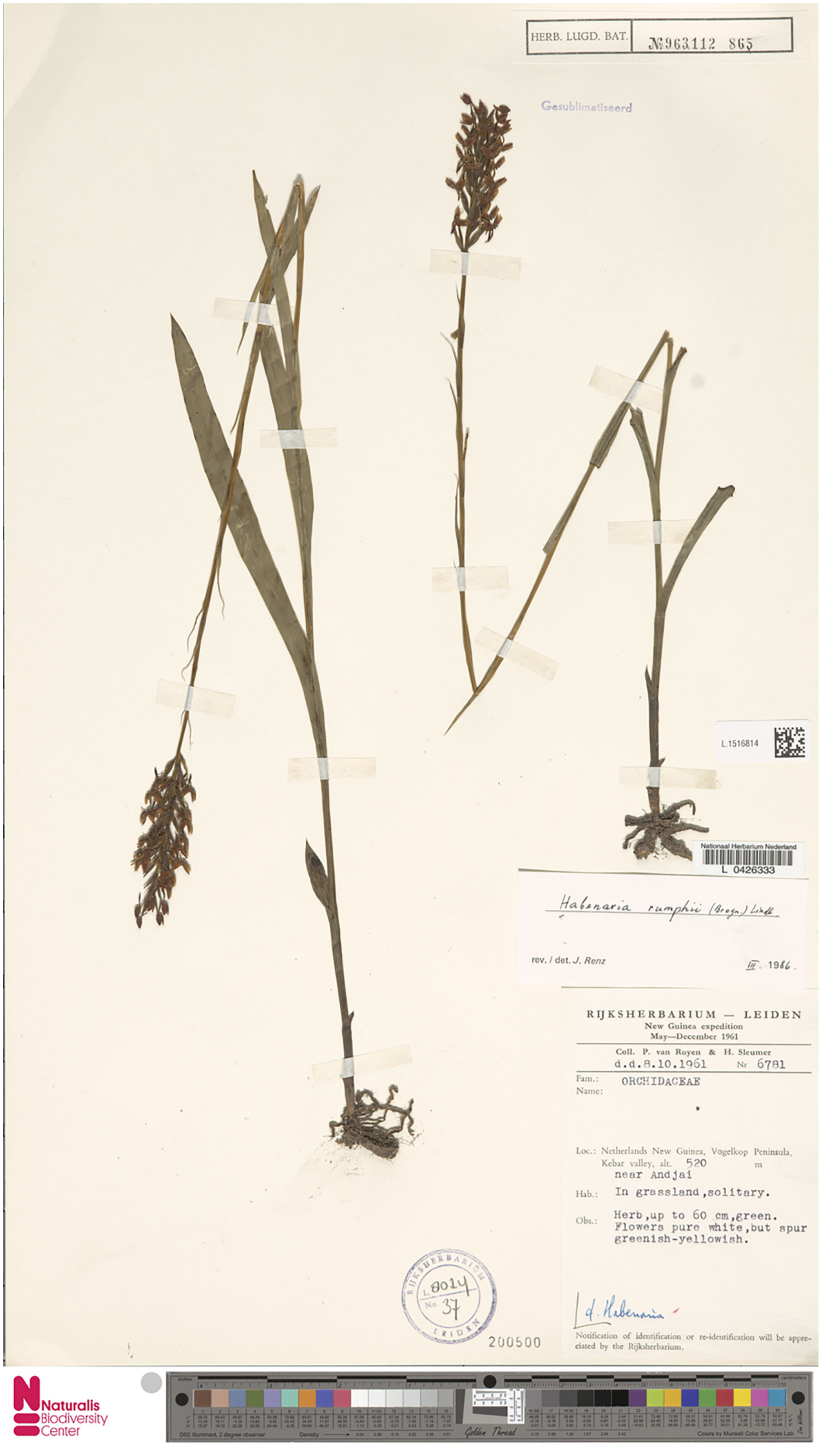
*Habenaria rumphii* (Brongn.) Lindl., herbarium specimen at Naturalis Biodiversity Center, L.1516814. (CC0 1.0; https://data.biodiversitydata.nl/naturalis/specimen/L.1516814).

**Figure 23:**
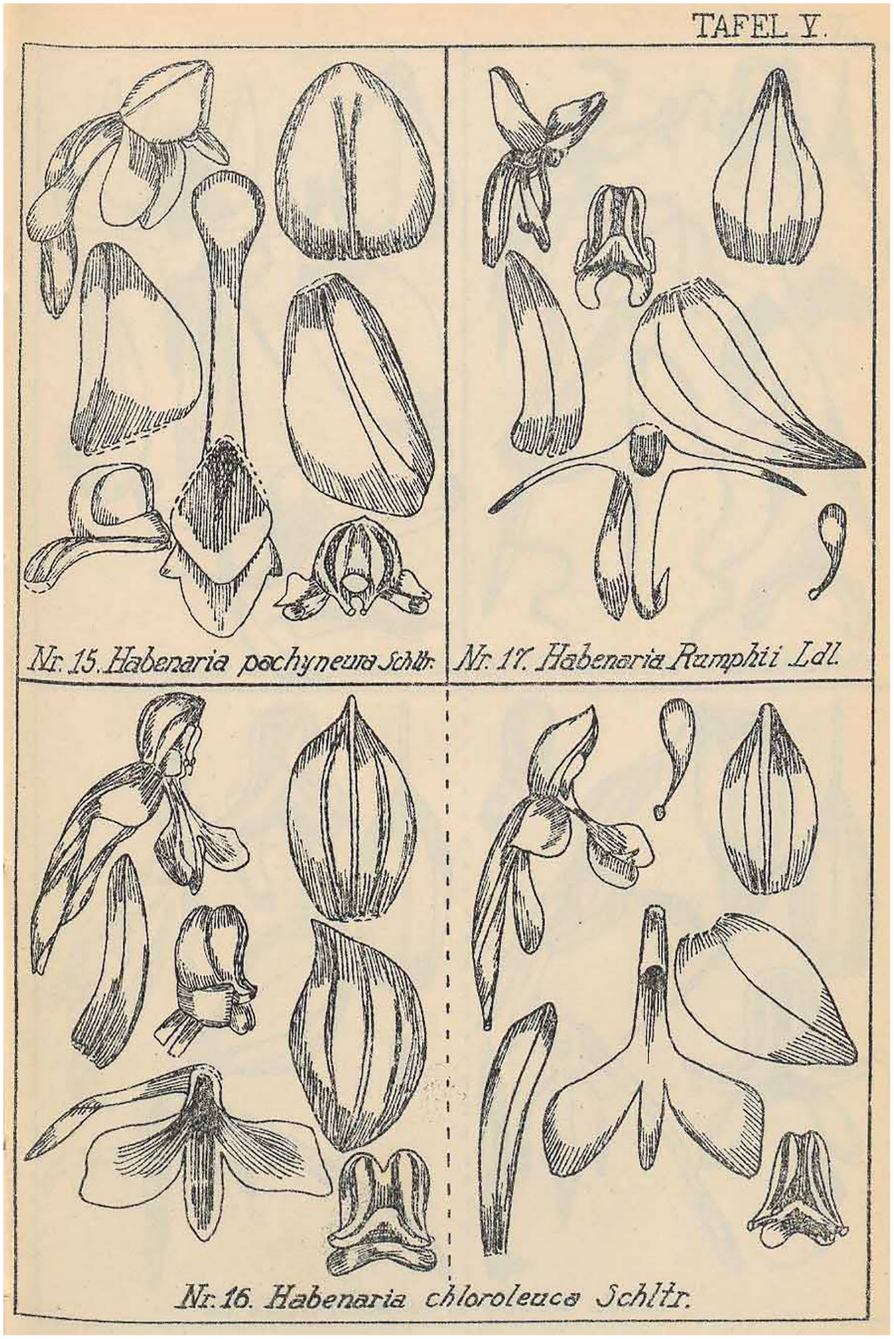
*Habenaria rumphii* (Brongn.) Lindl. and *H*. *baeuerlenii* F. Muell. & Kraenzl. *ex* Kraenzl. (as *H. chloroleuca* Schltr.). Drawings from Figurenatlas zu den Orchidaceen von Deutsch-Neu-Guinea, Feddes Repert. Spec. Nov. Regni Veg. Beih. 21(1), 1923 (Plate V, nr. 16 & 17). https://www.biodiversitylibrary.org/page/57688233.

**Figure 24:**
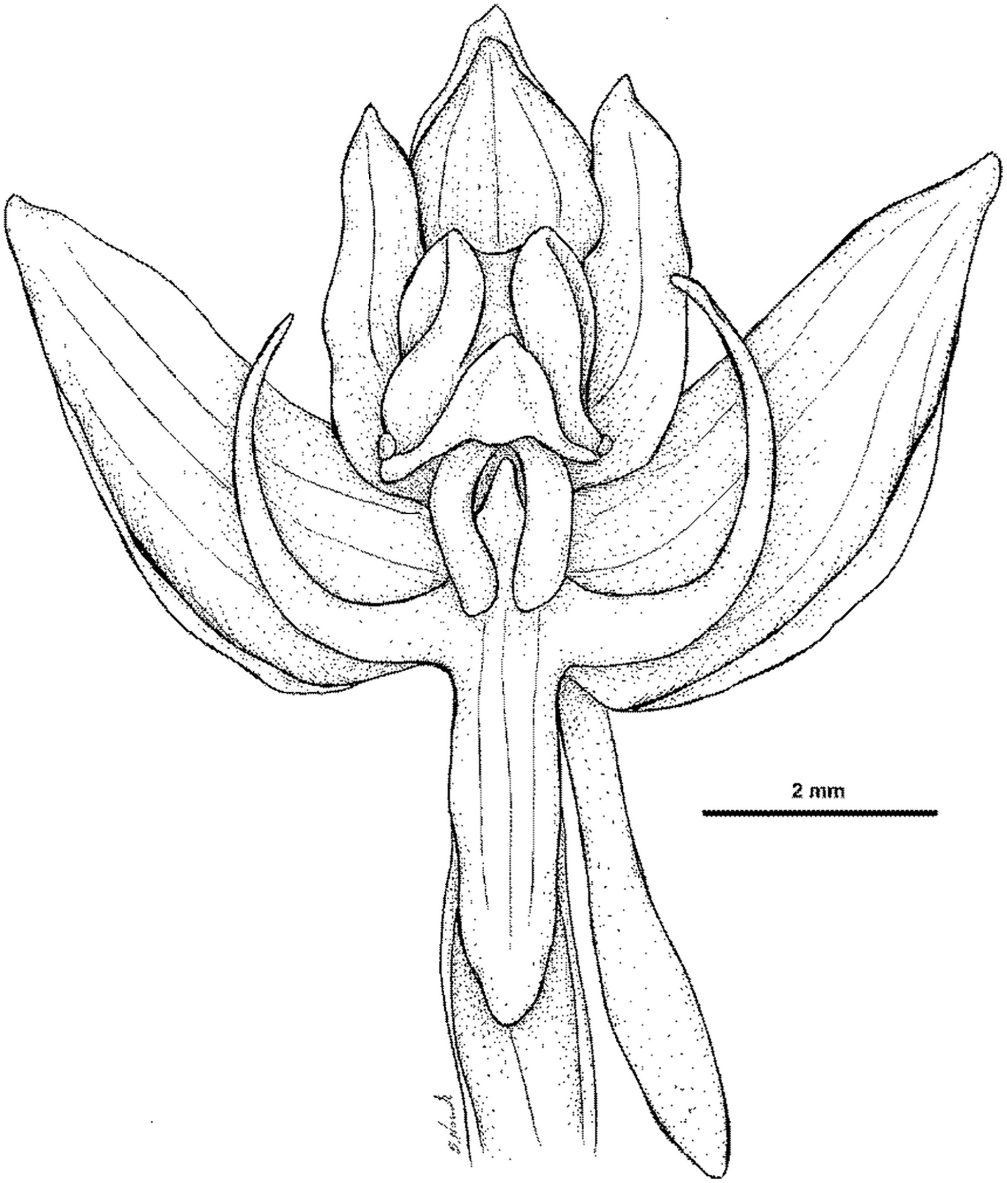
*Habenaria rumphii* (Brongn.) Lindl.: flower (drawn from *Brass 27334*; AMES).

Plant up to 50.0 cm tall, with 2–3 tubular sheaths at the base. Leaves 2–5, erect-patent, in the lower part of the stem, up to 17.0 cm long, 1.0–1.2 cm wide, narrowly lanceolate-linear, acute, gradually decreasing in size up the stem and transforming into bracts. Inflorescence up to 40.0 cm long, peduncle with 3–6 scales, rachis densely many-flowered. Floral bracts up to 20.0 mm long, subulate, apex long setaceous-acuminate. Pedicel with ovary 13.0–18.0 mm long, narrowly cylindrical, glabrous. Flowers usually white or dull green. Dorsal sepal 3.5–5.0 mm long, 2.0–3.0 mm wide, concave, broadly ovate to ovate, obtuse, apically somewhat cucullate, 3-veined. Lateral sepals 5.0–6.0 mm long, ca 2.0–3.0 mm wide, obliquely oblong ovate to elliptic-ovate, obtuse, 3-veined. Petals entire, 3.5–4.5 mm long, 1.0–1.8 mm wide, falcately oblong-lanceolate to lanceolate-ovate, subobtuse, 1- or 2-veined. Lip 3-lobed above short claw; middle lobe 5.0–9.0 mm long, 1.3–2.0 mm wide, ligulate-linear, obtuse; lateral lobes (1.8)3.0–4.0 mm long, 0.6–0.8 mm wide, linear-subulate, upcurved, long-acuminate to subobtuse, upcurved. Spur (1)4.0–6.0 mm long, narrowly cylindrical, apically somewhat swollen, obtuse. Gynostemium 2.0 mm long, stigmaphores slightly longer than the anther channels. Figs. 21–24.

*Habitat:* Terrestrial in savanna and grassy slopes. Alt. 50–100 m.

*Distribution*: Peninsular Malaysia, Sumatra, Java, Borneo, Celebes, the Moluccas, New Guinea, the Philippines, Thailand, Laos, Cambodia, Vietnam, Australia.

*Representative specimen*: Indonesia. Papua Prov. Merauke. Feb 1908. *B. Branderhorst 301* (BO!); Irian Jaya. Sentani. 21 Mar 1973. *J. Raynal 16740* (BR!, P!); Netherlands New Guinea. Vogelkop Peninsula. Kebar valley, near Andjar. Alt. 520 m. 8 Oct 1961. *P. van Royen & H. Sleumer 6781* (L!); Netherlands New Guinea. Vogelkop Peninsula. Kebar valley, near Andjar. Alt. 550 m. 13 Dec 1961. *P. van Royen & H. Sleumer 8250* (L!); Papua. Morapasupuwara. Alt. 500 ft. 1951. *N.E.G. Cruttwell 318* (AMES!, K!); Morobe District. Radarada. Alt. 1500 ft. 11 Apr 1961. *N.E.G. Cruttwell 1160* (K!); Morobe Prov., above Markham Point, near Lae. Alt. 100 m. 16 Aug 1979. *J. Renz 12416* (RENZ!); Sepik Distr., Leitre village. Alt. 60 m. 17 Mar 1964. *C.D. Sayers 12250* (RENZ!); Morobe District, Markham Point near Lae. Alt. 200 ft. 28 Sep 1966. *H. Streiman & A. Kairo NGF 27913* (AMES!, K!, L!, LAE!); Morobe District, Malalo-Miso. Alt. 900 ft. 30 Oct 1936. *M.S. Clemens 4326* (AMES!); Dobodura-Buna area. 10 Jan 1944. *J.R. Reeder 861* (AMES!); Western Division. Dagwa, Orimo River. Alt. 45 m. Feb-Mar 1984. *L.J. Brass 5913* (AMES!, NY!); Morobe District. Markham Point – bluff along the Markham River, about 7 miles W of Lae. 3 Oct 1963. Alt. 150 ft. *T.G. Hartley TGH 12246* (AMES!, K!, L!, LAE!); Maprik Subdistr., Hayfield, Kungingini Road. Alt. 400 ft. 10 Dec 1954. *J.S. Womersley & N.W. Simmonds 6870* (LAE!); Sepik Distr. Laitre village. Alt. 200 ft. 14 Mar 1964. *C.D. Sayers NGF 18909* (L!, LAE!); Finschhafen. *M.U. Hollrung 319* (B†); Land of Wilhelm. Ralum. Grassland. [New Britain, Mother Volcano]. Alt. 700 m. Mar 1897. *F.O. Dahl s.n*. (B†); Fergusson Isl., Deidel, Gomwa Bay. Edge of a path in Melaleuca savanna. *L.J. Brass 27334* (AMES!, L!, LAE!). Fig. 20.

*Notes: Habenaria rumphii* is similar to *H. khasiana* and the differences between these taxa 2are discussed above. It differs from *H. ochroleuca* by having broader leaves (1–1.2 cm *vs* 0.4–0.8 cm), much shorter floral bracts (8 mm *vs* 20 mm), and a longer spur (11–13 mm *vs* 1–6 mm).

2.3. ***Habenaria torricellensis*-subgroup**

Leaves basal, oblanceolate to obovate-elliptic. Petals undivided. Lip 3-lobed, lobes entire. Anther narrowly obovoid, antherophores longer than stigmaphores, auricles sessile, prominent.

### Key to species of *Habenaria torricellensis-*subgroup

1. Lip lobes dissimilar, lateral lobes cuneate-rhomboid, apically obliquely truncate-rounded 2.3.1. ***H. baeuerlenii***
1* Lip lobes similar, narrow, linear to linear-lanceolate 2
2. Lip middle lobe linear-lanceolate, acute, with incurved margins forming a kind of channel 2.3.3. ***H. cruciata***
2* Lip middle lobe flat or convex, but never channeled 3
3. Lip lateral lobes obliquely truncate at the apex, shortly and irregularly bilobulate-emarginate 2.3.5. ***H. ensigera***
3* Lip lateral lobes obtuse to subacute at the apex 4
4. Lip middle lobe with two elevated plates at the base 2.3.4. ***H. drepanodes***
4* Lip middle lobe without any plate 5
5. Ovary minutely ciliate along keels 2.3.6. ***H. rechingeri***
5* Ovary glabrous 6
6. Upper margin of sepals minutely ciliolate 2.3.2. ***H. bougainvilleae***
6* Sepals glabrous 7
7. Spur cylindrical-filiform, equally wide towards the apex, sepals up to 8 mm long 3.7. ***H. retroflexa***
7* Spur cylindrical, swollen in the apical third, sepals 9.5-12.5 mm long 2.3.8. ***H. torricellensis***

2.3.1. ***Habenaria baeuerlenii*** F. Muell. & Kraenzl. *ex* Kraenzl., Bot. Jahrb. Syst. 67: 488. 1893. TYPE: New Guinea. *W. Bäuerlen s.n*. (B†); *H. Streimann & A. Kairo 47954* (Neotype, *designated here*: LAE!, Isoneotypes: A!, BO!, K!, L!). ≡ *Pecteilis bauerlenii* (F.Muell. & Kraenzl.) M. A. Clem. & D. L. Jones, Austral. Orchid Rev. 83(6): 51. 2018, *syn. nov*.

= *Habenaria viridi-alba* P. F. Hunt. *in* Kew Bull. 24: 75. 1970. ≡ *Habenaria chloroleuca* Schltr. *in* K. Schum. & Lauterb., Fl. Schutzgeb. Südsee: 77. 1905, *nom. illeg*. TYPE: Papua New Guinea (Deutsch Neu-Guinea). *R. Schlechter 13936* (B†). ≡ *Plantaginorchis chloroleuca* (Schltr.) Szlach., Richardiana 4: 64. 2004, *nom. illeg*.

= *Habenaria turneri* R.S.Rogers, Trans. & Proc. Roy. Soc. South Australia 49: 254. 1925. ≡ *Peristylus turneri* (R.S.Rogers) W.Kittr., Bot. Mus. Leafl., Harvard Univ. 30: 95. 1985. TYPE: Papua New Guinea. *L. Turner s.n*. (AD, BRI)

**Figure 25:**
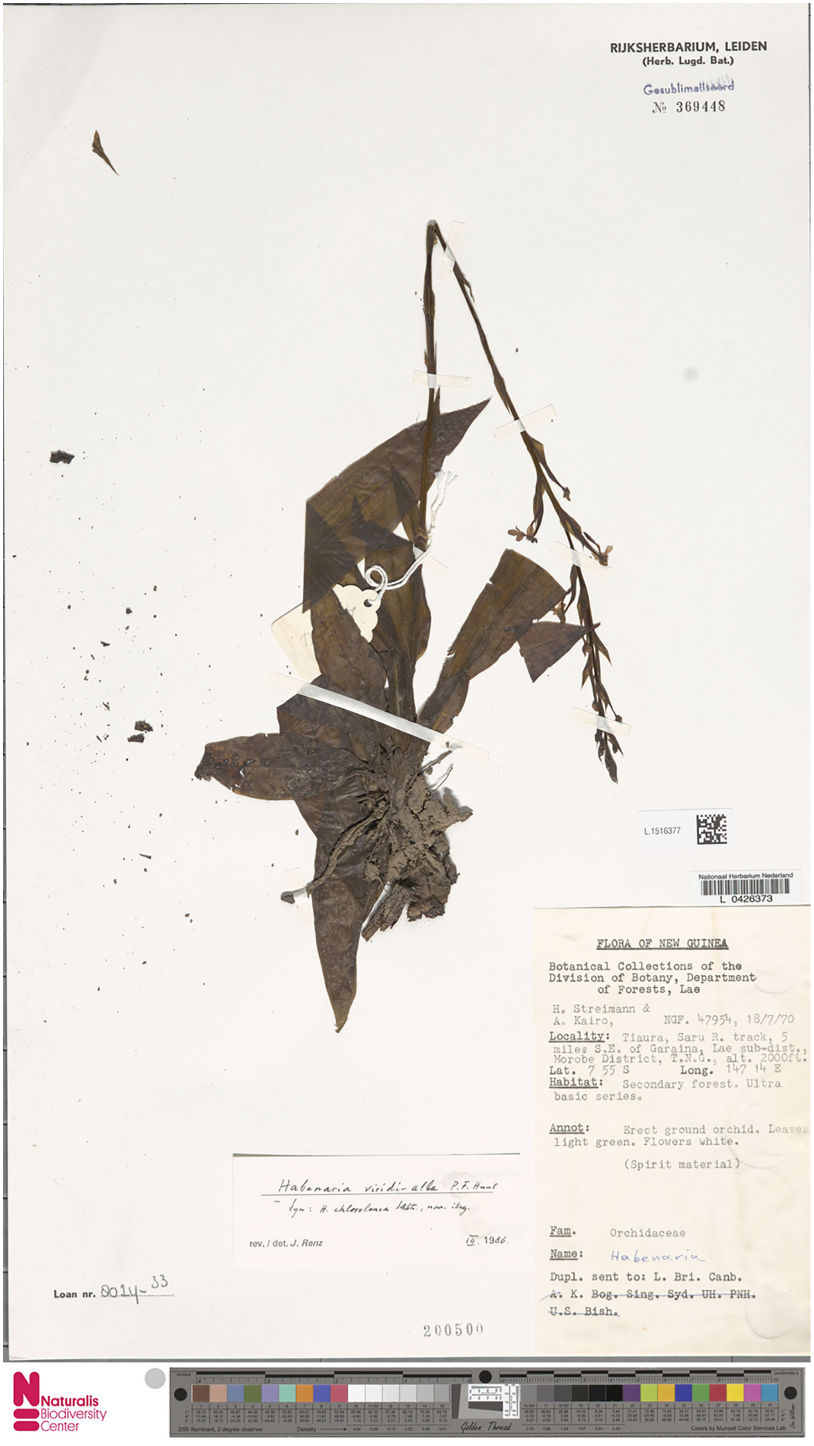
*Habenaria baeuerlenii* F. Muell. & Kraenzl. *ex* Kraenzl., herbarium specimen at Naturalis Biodiversity Center, L.1516377. (CC0 1.0; https://data.biodiversitydata.nl/naturalis/specimen/L.1516377).

**Figure 26:**
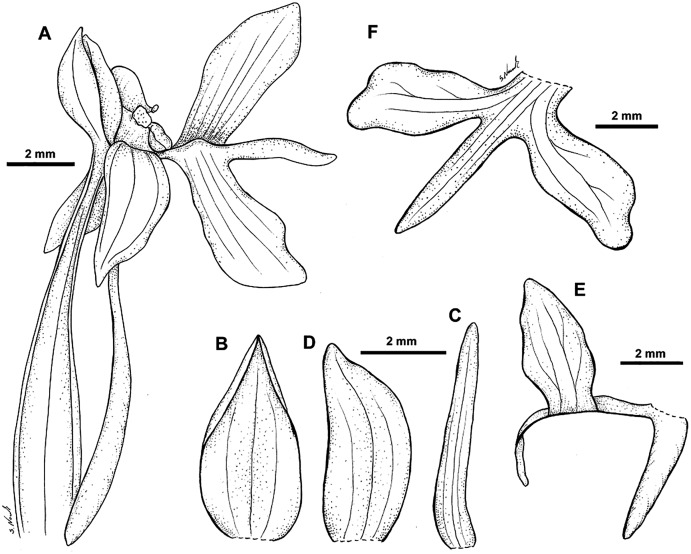
*Habenaria baeuerlenii* F. Muell. & Kraenzl. *ex* Kraenzl.: (A) flower; (B) dorsal sepal; (C) petal; (D) lateral sepal; (E) lip with short-form spur; (F) lip. Drawn from *Brass 27295*; (A)—AMES; *Sands 1158*;(B)–(E)—K; *Streiman & Kairo 47954*; F—B.

Plant 35.0–60.0 cm tall, basally leafy. Leaves several, erect-patent, 15.0–22.0 cm long, 2.2–3.3 cm wide, oblanceolate, acute; sterile bracts 3, lanceolate to ovate, acute. Inflorescences 10.0–15.0 cm long, laxly many-flowered. Floral bracts 13.0–16.0 mm long, ovate-lanceolate, acuminate. Pedicel with ovary 15.0–24.0 mm long, basally cylindrical, attenuate towards the apex. Flowers white or with greenish sepals and pure white petals and lip. Dorsal sepal 4.1–5.0 mm long, 2.4–3.0 mm wide, concave, elliptic-ovate, apex obtuse, 3-veined. Lateral sepals 4.0–4.5 mm long, 2.2–2.6 mm wide, obliquely ovate-lanceolate, apex obtuse to subacute, 3-veined. Petals entire, 4.0–5.0 mm long, 0.8–0.9 mm wide, ligulate, slightly falcate, apex obtuse, 1- or 3-veined. Lip 3-lobed above 1.0 mm long claw; middle lobe 3.0–4.5 mm long, 0.9 mm wide, linear, obtuse, thick along midvein; lateral lobes 5.0–5.5 mm long, 2.7–3.0 mm wide, cuneate-rhomboid, apically obliquely truncate-rounded. Spur (4.9)12.0–13.0 mm long, cylindrical, sometimes slightly clavate towards subobtuse apex. Gynostemium 2.5–3.5 mm long; stigmaphores slightly longer than anther channels. Figs. 23, 25–26.

*Habitat:* Terrestrial in rainforest and in forest and cliffs in the rivers valleys, found also in pine forest. Alt. 10–1,600 m.

*Distribution*: New Guinea.

**Figure 27:**
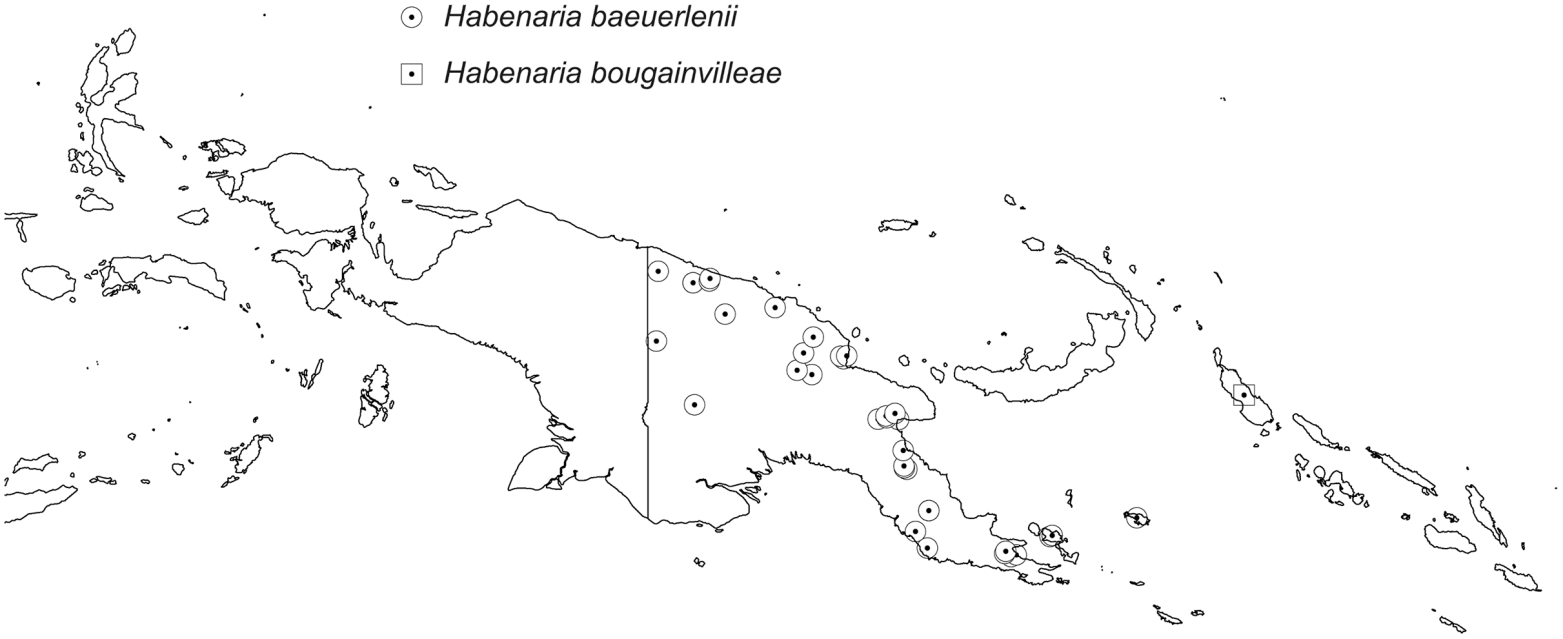
Distribution of *Habenaria baeuerlenii* and *H. bougainvillae*. Base map downloaded from www.naturalearthdata.com.

*Notes: Habenaria baeuerlenii* is a very easily distinguishable species due to cuneate-rhomboid lateral lobes of lip, not present in any other New Guinean species.

*Representative specimens*: Papua New Guinea. Rigo District. *L. Turner s.n*. (AD, BRI); Strickland River. *W. Bäuerlen s.n*. (B†); Walder, Mts. Bismarck. Alt. 700 m. 12 Jan. 1902. *R. Schlechter 13936* (B†); Musom River. 6°45′S 147°00′E. Alt. 300 ft. 16 Sep 1971. *P. Katik NGF46821* (K!, L!, LAE!); Tiaura, Saru R. track, 5 miles SE of Garaina. 7°55′S 147°14′E. Alt. 2000 ft. 18 Jul 1970. *H. Streimann & A. Kairo 47954* (A!, BO!, K!, L!, LAE!); SE of Opanabu village. 10°01′S 149°42′E. Alt. 700 m. 11 Jul 1969. *A. Kanis 1252* (A!, K!, L!, LAE!, RENZ!); Wusu Creek, Parakob *via* Sibilanga. Alt. 500 m. Jul 1981. *T.M. Reeve 564* (K!, L!, LAE!); Kaiser-Wilhelmsland. Alt. 1,600 m. 18 Jul 1908. *R. Schlechter 18042* (AMES!, BM!, E!, K!, L!, RENZ!); The same locality. Alt. 500 m. Feb 1908. *R. Schlechter 17311* (E!, K!, L!); The same locality. Alt. 1,000 m. Jun 1909. *R. Schlechter 19754* (AMES!, E!, K!); The same locality. Jan. 1909. *R. Schlechter 19347* (BO!, E!); Somadjidji. Alt. 400 m. May 1909. *R. Schlechter 17347* (K!); Morobe Distr. Buso River. 7°30′S 147°15′E. Alt. 100 ft. *A.N. Gillison & al. NGF 25685* (K!, L!, LAE!); Morobe Distr., Markham Valley, Kajabit. *M.S. Clemens 10566bis* (AMES!); Tagan river valley. Alt. 300 ft. Jun 1955. *J. S. Womersley & A. Millar 7718* (AMES!, K!, LAE!); Raba Raba, Mayu camp I, junction of Mayu & Ugat Rivers, Mt. Sucklin. 9°37′S 149°10′E. Alt. 305 m. 20 Jun 1972. *G.J. Leach & P. Katik LAE56057* (K!); Bewani Mts., Kilifas on the river Yenabu. Alt. 280 m. 27 Mar 1970. *M.J. Sands 1158* (K!); West Sepik Prov., Anguganak, Lumi District. Alt. 300 m. Jul 1981. *T.M. Reeve 611* (LAE!, RENZ!); Lumi District. Omgan Creek. Alt. 750 m. Jul 1981. *T.M. Reeve 612* (LAE!); Wusu Creek, Parakob *via* Sibilanga (Torricelli Mts), Lumi District. Alt. 500 m. Jul 1981. *T.M. Reeve 564* (RENZ!); Northern Distr., Dive. Alt. 450 m. 27 Jul 1964. *A. Millar 23532* (LAE!, RENZ!); Fergusson Isl., Mts. between Agamoia and Ailuluai. Alt. 200 m. 18 Jun 1956. *L.J. Brass 27219* (L!); Fergusson Isl. Agamoia. Alt. 200 m. 23 Jun 1956. *L.J. Brass 27295* (AMES!, L!); Morobe Prov. Wau subprovince. Garaina (in Klinkii forest near Lutheran Mission), Alt. 650 m. 14 Sep 1979. *N.H.S. Howcroft 64075* (LAE!); Sepik District, Ambunti subdistrict. Along Yapa (Hunstein River). Alt. ca. 500 ft. 30 Jul 1966. *R.D. Hoogland & L.A. Craven 10769* (CANB, L!); Madang District. Near Mawan village, Gogol Valley. Alt. 325 m. 20 Jun 1955. *R.D. Hoogland 4911* (LAE!), Madang Province, Ohu Forest. Alt. 20 m. 13 Jul 2000. *T. Motley et al. 2340* (NY!), East Sepik District. Angoram subdistrict. Amboin. Alt. 250 ft. 28 Jul 1967. *A. Millar NGF 35139* (LAE!); Morobe District. Busu River. Alt. 50 ft. 23 Jun 1965. *A. Millar NGF 22593* (L!, LAE!); Western Highlands. Jimi Valley. Alt. 2200 ft. Jun 1955. *J.S. Womersley & al. NGF 7726* (LAE!); Madang Distr., Tiganuntz River, near Aiome, tributary of main stream. Alt. 250 ft. 30 Jun 1966. *J.S. Womersley NGF 24758* (LAE!); Madang Prov. Josephstaal FMA area, near Kumamdeber, along trail to Morasapa, W of expedition Camp 1. Alt. 160 m. 30 Jul 1999. *W.N. Takeuchi & al. 13525* (LAE!, UPNG!); Morobe Distr. Wau subdistr., Garaina, Waria river Gorge. Alt. 2000 ft. 12 May 1971. *B.C.M. Stone 10215* (LAE!); Milne Bay District. Binigui Camp, Gwari River. Alt. 200 m. 2 Aug. *L.J. Brass 23779* (AMES!, L!, LAE!); Kulumadau, Woodlark Isl. 12 Nov 1956. *L.J. Brass 28721* (AMES!, L!, LAE!, US!); Kokoda. Alt. 1200 ft. 4 Apr 1936. *C.E. Carr 10652* (B!, BM!, K!, L!, LAE!, NY!); Kokoda. 29 May 1936. *C.E. Carr 17255* (BM!); Madang Distr., Madang subdistr., Near Forestry Station Gogol. 10 Jul 1971. *P. Stevens LAE54709* (L!, LAE!); Morbe Distr. Bupu village, Wampit. Alt. 4300 ft. 13 Jul 1967. *A. Millar NGF 22950* (A!, L!, LAE!); Lae subprovince, near Oomsis river, Oomsis Logging road. Alt. 300 m. 4 Nov 1985. *P. Katik LAE77972* (L!, LAE!, NSW); Gwadagwada. Alt. 700 ft. 8 May 1954. *N.E.G. Cruttwell 408* (E!, K!); Waitarua. Alt. 1600 ft. 15 Jun 1951. *N.E.G. Cruttwell 287* (K!); Kanasura. Alt. 1750 ft. 15 Jun 1951. *N.E.G. Cruttwell 280* (K!); Kokoda. Alt. 1250 ft. 27 May 1933. *L.E. Cheesman 38* (K!); Kokoda. Alt. 1200 ft. 17 Apr 1933. *L.E. Cheesman 77* (K!); Central Province. Sogeri Plateau, road to Sirinumu Dam. Ca. 20 km SE past dam. Alt. 720 m. 2 Jul 1977. *M. Fallen 252* (LAE!); Morobe Distr., Atyera Range. *Sine coll. NGF3212* (AMES!, K!). Fig. 27.

We decided to designate the neotype because no original material could be traced. We decided to select a collection of *Streimann & Kairo 47954*, that is very well preserved and found in many herbaria. Moreover, *Habenaria baeuerlenii* shows a high variability, so we chose a fairly typical representative of this taxon, corresponding well to the original description.

2.3.2. ***Habenaria bougainvilleae*** Renz, Pl. Syst. Evol. 155: 326. 1987. TYPE: Solomon Islands. *P. Lavarack & C. Ridsdale NGF 31153* (Holotype: LAE!, Isotypes: K!, RENZ!)

**Figure 28:**
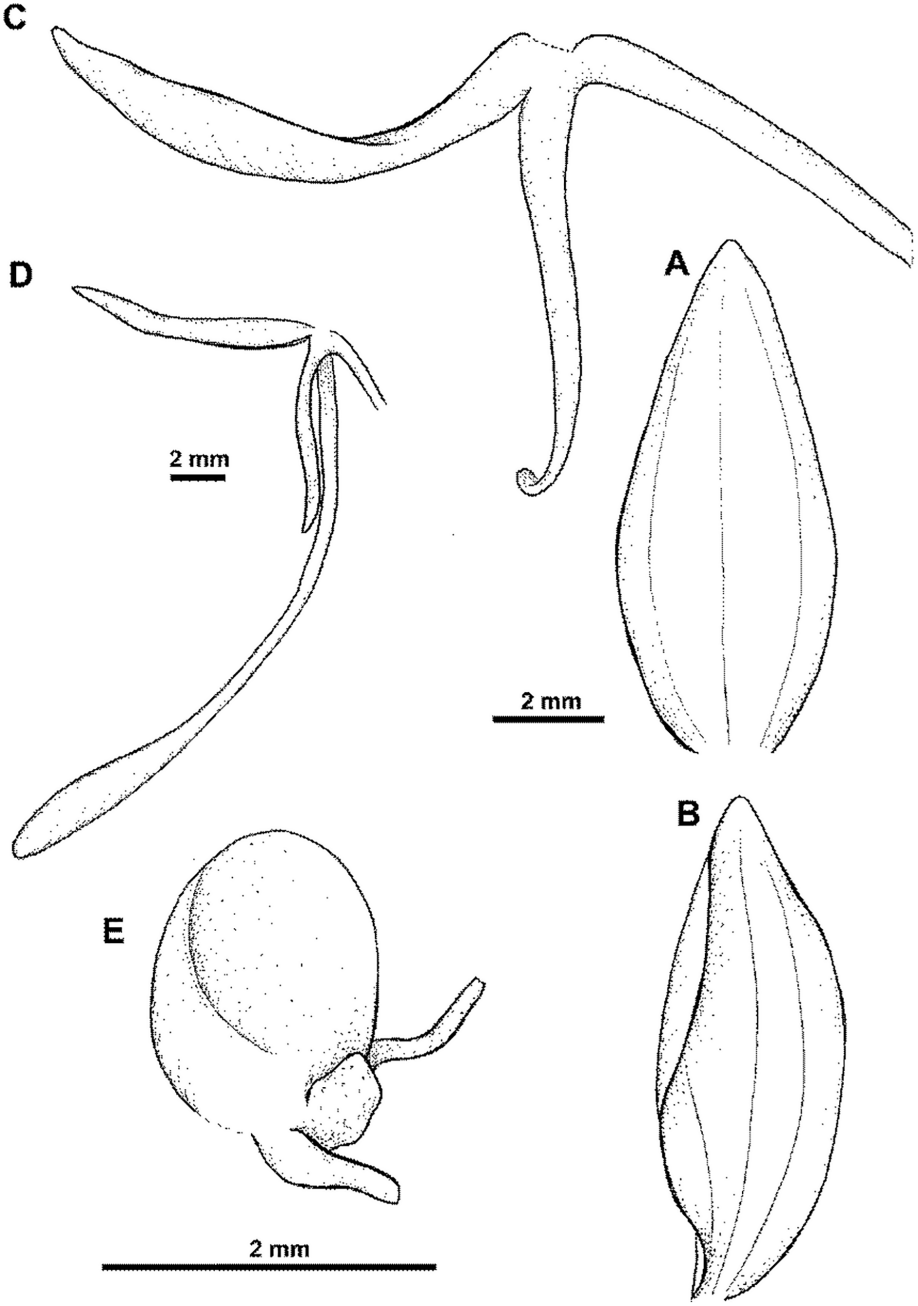
*Habenaria bougainvillae* Renz: (A) dorsal sepal; (B) lateral sepal; (C) lip; (D) lip with spur; (E) gynostemium (drawn from *Lavarack & Ridsdale NGF31153*; K).

Plant up to 70.0 cm tall, base with closely clasping sheaths, third part leafy. Leaves 4–6, erect-patent, 15.0–25.0 cm long, 3.0–3.5 cm wide, oblanceolate, acute, decreasing in size towards the stem. Inflorescence 15.0–20.0 cm long, cylindrical, subdensely 10–15-flowered. Floral bracts about 20.0–22.0 mm long, ovate-lanceolate, acute. Pedicel with ovary 22.0–25.0 mm long, narrowly cylindrical. Flowers white. Dorsal sepal 6.0–9.0 mm long, up to 4.0 mm wide, cucullate-concave, broadly ovate, acute, 3- or 5-veined, upper margin minutely ciliolate. Lateral sepals 6.0–9.0 mm long, 3.0–4.0 mm wide, deflexed, obliquely obovate-triangular, acute, 3-veined, upper margin minutely ciliolate. Petals 6.0–9.0 mm long, 2.0–2.5 mm wide, ligulate-lanceolate, obtuse, 2-veined. Lip 3-lobed just above the base; middle lobe 8.0–9.0 mm long, linear-lanceolate, obtuse to acute; lateral lobes 9.0–11.0 mm long, linear lanceolate to linear, obtuse to acute, divergent. Spur 17.0–25.0 mm long, narrowly cylindrical, swollen in the apical part, blunt. Gynostemium 2.0 mm long, anther channels 2.0 mm long; stigmaphores oblong, 1.0 mm long. Fig. 28.

*Habitat:* Terrestrial on a hillside on the rainforest floor. Alt. 850 m.

*Distribution*: Solomon Isl.

*Representative specimen*: Solomon Islands. Bougainville Distr., ridge behind Pavairi, rain-forest floor, hillside. Alt. 850 m. 24 Jan 1967. *P. Lavarack & C. Ridsdale NGF 31153* (K!, LAE!, RENZ!). Fig. 27.

*Notes:* The taxonomic separateness of *Habenaria bougainvilleae* requires further study. This species and *H. torricellensis* share similar form of lateral sepals, but in *H. bougainvilleae* the upper margin of lateral sepals is minutely ciliolate (*vs* glabrous). Moreover, flowers of *H. torricellensis* are somewhat larger. *H. bougainvilleae* can be distinguished from *H. rechingeri* by the glabrous ovary (*vs* minutely ciliate in the latter).

Among the distinguishing characters in relation to *H. trichoglossa*, *H. rechingeri*, *H. torricillensis, H. retroflexa* and *H. cruciata*, the most conspicuous are the narrower leaves, which are inserted on the lower half of the stem, but not basal, and the hardly spreading flowers giving the impression of a narrowly cylindrical inflorescence. Also the white colour of the flower among the forest Habenarias of New Guinea and the adjacent is uncommon, all other are green or dark blue-green (Renz, 1987). According to Lewis & Cribb (1991) petals of *H. bougainvilleae* are ovate-lanceolate whereas in other similar species they are linear.

2.3.3. ***Habenaria cruciata*** J. J. Sm., Bull. Dep. Agric. Indes Neerl. 19: 25. 1908. TYPE: Irian Jaya (Dutch New Guinea). *G.M. Versteeg 1535* (Lectotype, *designated here*: L! Isolectotypes: BO?, K!; RENZ!—photo).

**Figure 29:**
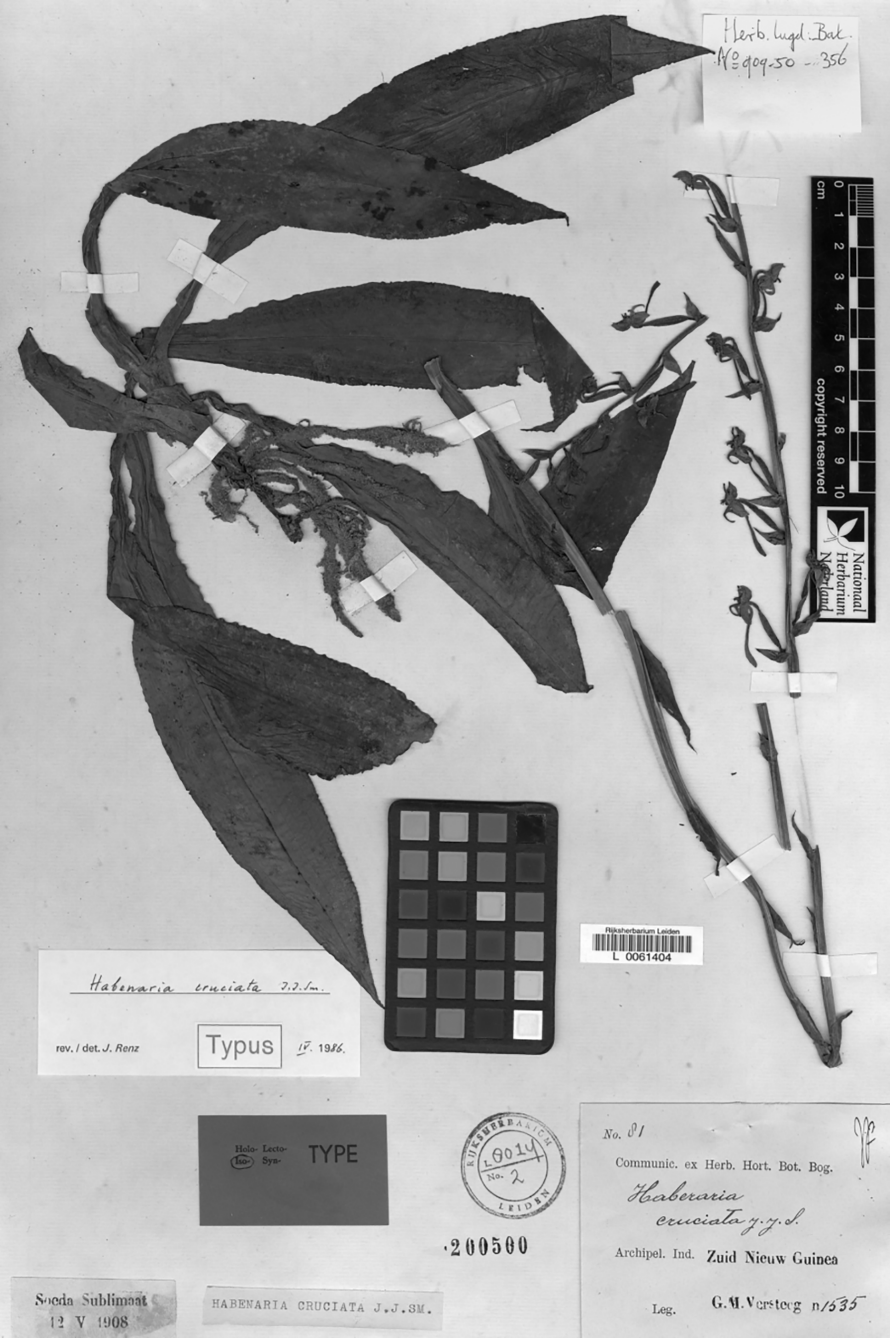
*Habenaria cruciata* J.J.Sm., type specimen at Naturalis Biodiversity Center, L 0061404. (CC0 1.0; https://data.biodiversitydata.nl/naturalis/specimen/L%20%200061404).

**Figure 30:**
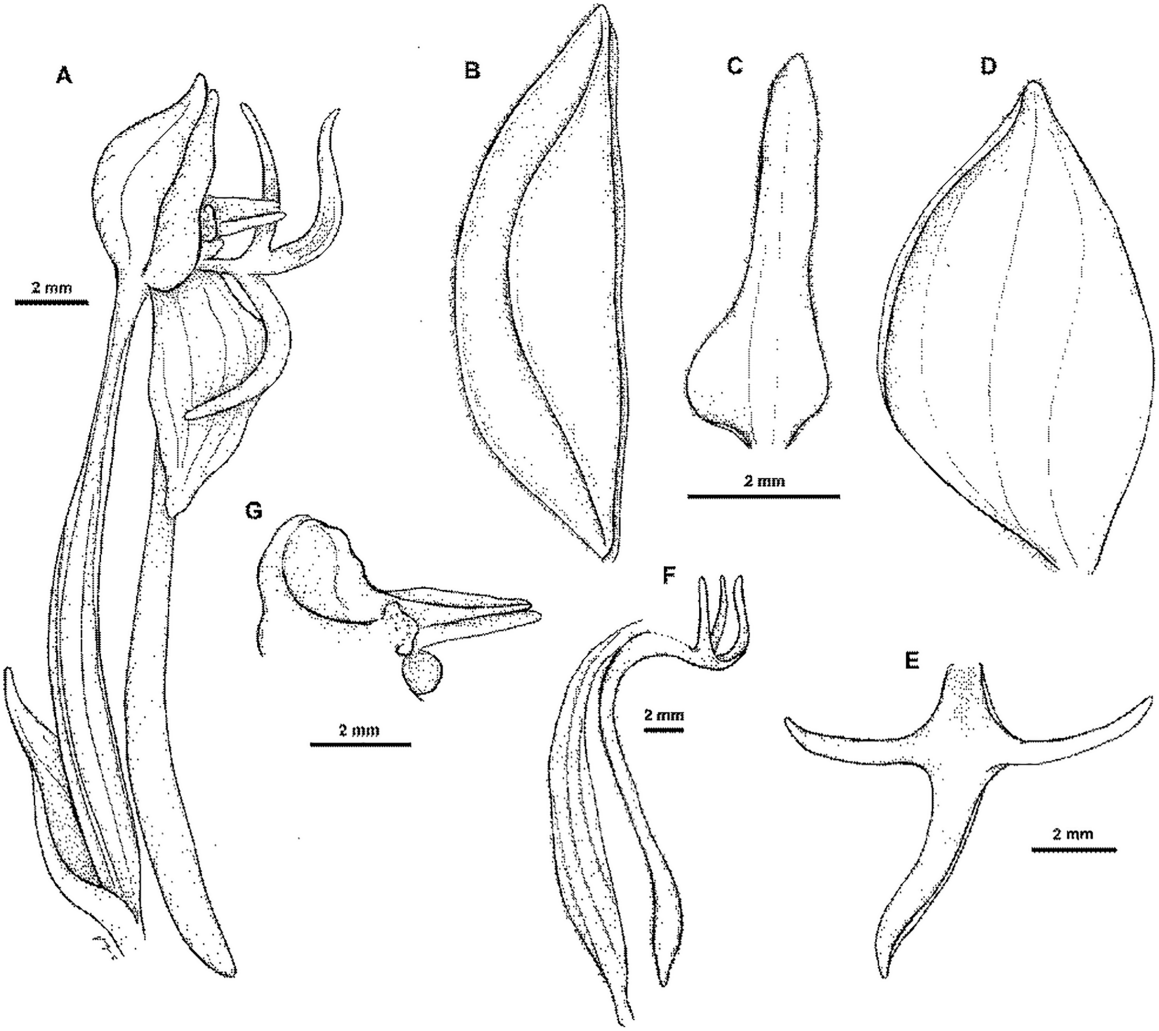
*Habenaria cruciata* J. J. Sm.: (A) flower; (B) dorsal sepal; (C) petal; (D) lateral sepal; (E) lip; (F) lip with spur and ovary; (G) gynostemium. Drawn from *Moeljono & Jitmau 7656*; (A) & (G)—AMES; *Versteeg 1535*; (B)–(F)—K.

Plant up to about 80.0 cm tall, basally leafy. Leaves 7–10, 12.0–27.0 cm long, 2.7–4.3 cm wide, oblanceolate to elliptic-oblanceolate, acutely acuminate, minutely undulate, sheaths 2.0–3.0 cm long, tubular. Inflorescence erect, peduncle hirsute-pubescent especially in the upper part, sheaths foliaceous, ovate-lanceolate, minutely ciliolate; rachis 22.0–35.0 cm long, hirsute-pubescent, laxly many-flowered. Floral bracts (9)15.0–22.0 mm long, suborbicular-ovate, acutely acuminate, sometimes the larger veins dorsally keeled and ciliate. Pedicel with ovary 30.0 mm long, incurved, 6-ribbed, ribs hirsute-pubescent, attenuate towards apex, basally somewhat swollen. Flowers greenish white. Dorsal sepal 6.0–8.5 mm long, 5.0–6.0 mm wide, cucullate-concave, broadly ovate, obtuse, slightly denticulate, 3-veined, veins dorsally strongly winged-keeled and pubescent-ciliate. Lateral sepals 6.0–8.0 mm long, 3.8–5.0 mm wide, obliquely elliptic-ovate, narrowly obtuse, strongly reflexed, 3-veined, veins dorsally strongly winged-keeled and pubescent-ciliate. Petals 5.5–9.0 mm long, 1.2–3.5 mm wide at the base, convex, obliquely ovate to ligulate-lanceolate, obtuse, shortly ciliolate, 3-veined. Lip 3-lobed above 2.0 mm long claw, cruciform; middle lobe 5.5–7.5 mm long, 1.0–1.2 mm wide, linear-lanceolate, acute, with upcurved margins forming a kind of channel; lateral lobes 4.5–5.5 mm long, 0.8 mm wide, linear, subacute, slightly upcurved. Spur 20.0–23.0 mm long, decurved above the base, cylindrical, slightly swollen above the middle and then attenuate towards subacute apex, above the base laterally compressed and ciliate. Gynostemium 4.5 mm long, anther channels elongated; stigmaphores thickened at the apex, much shorter than the anther channels. Figs. 29–30.

*Habitat:* Terrestrial in lowland rainforest and premontane forest, mixed forest and damp forest. Alt. 200-1000 m.

*Distribution*: New Guinea.

**Figure 31:**
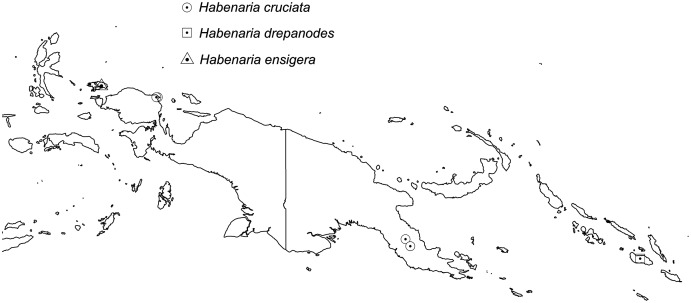
Distribution of *Habenaria cruciata, H. drepanodes* and *H. ensigera*. Base map downloaded from www.naturalearthdata.com.

*Notes: Habenaria cruciata* belongs to a group of species with a cruciate lip, and where the lateral lobes are shorter than or equal to middle lobe. However, it can be distinguished by the strongly winged-keeled and pubescent-ciliate sepals, and the lip middle lobe forming a kind of channel. This species can be confused with *H. retroflexa*, in which however, the basal part of the lip is not channelled. The status of both species requires further study.

*Representative specimen*: Indonesia. Prov. Papua. (Dutch New Guinea). On the Noord River, south of Geluks Hill in primary forest. Jul 1907. *G.M. Versteeg 1535* (BO?, K!, L!, RENZ!); Reserve area on uplifted coral to north of Manokwari Mixed lowland rain forest dominated by *Pometia, Intsia, Chisocheton, Terminalia* etc. Alt. 200 m. 17 Mar 1994. *S. Moeljono & M. Jitmau 7656* (AMES!, E!, K!); Netherlands New Guinea. Kebar valley, c. 10 km W of Manokwari. Alt. c. 580 m. 25 Oct 1954. *P. van Royen 3815* (L!). Papua New Guinea. Morobe Distr. Along Waria River, below Garaima. Alt. 1,000 m. 20 Jun 1962. *T.G. Hartley TGH 10365* (AMES!, K!, L!, LAE!, RENZ!); *A. Millar NGF 22659* (K!, L!, LAE!); Central Distr. Kokoda, damp forest by stream. Alt. 1200 ft. 4 Apr 1936. *C.E. Carr 10635* (AMES!, B!, BM!, K!, L!, LAE!, NY!, P!); Milne Bay Distr. Knanasuru. Alt. 2100 ft. 24 Jun 1959. *N.E.G. Cruttwell 1000* (K!, L!); Kokoda. Alt. 1200 ft. 6 May 1933. *L.E. Cheesman 44* (K!); Kokoda. Alt. 2000 ft. 11 Oct 1933. *L.E. Cheesman 125* (K!). Fig. 31.

Schuiteman, Vermeulen & De Vogel (2001–2010) and De Vogel, Vermeulen & Schuiteman (in Press) suggest that *Versteeg 1535* (as holotype) may be at BO, but we have not been able to confirm this either. However, at least two specimens have been found (L and K), and we decided to go with the specimen in L as lectotype, which unquestionably corresponds to the protologue.

2.3.4. ***Habenaria drepanodes*** Renz *ex* Kolan., S. Nowak & Szlach., Ann. Bot. Fenn. 52(5–6): 329. 2015. TYPE: Solomon Islands. *A. Nakisi BSIP8024* (Holotype: LAE!; Isotypes: L!, K!, RENZ!—photo and drawing).

**Figure 32:**
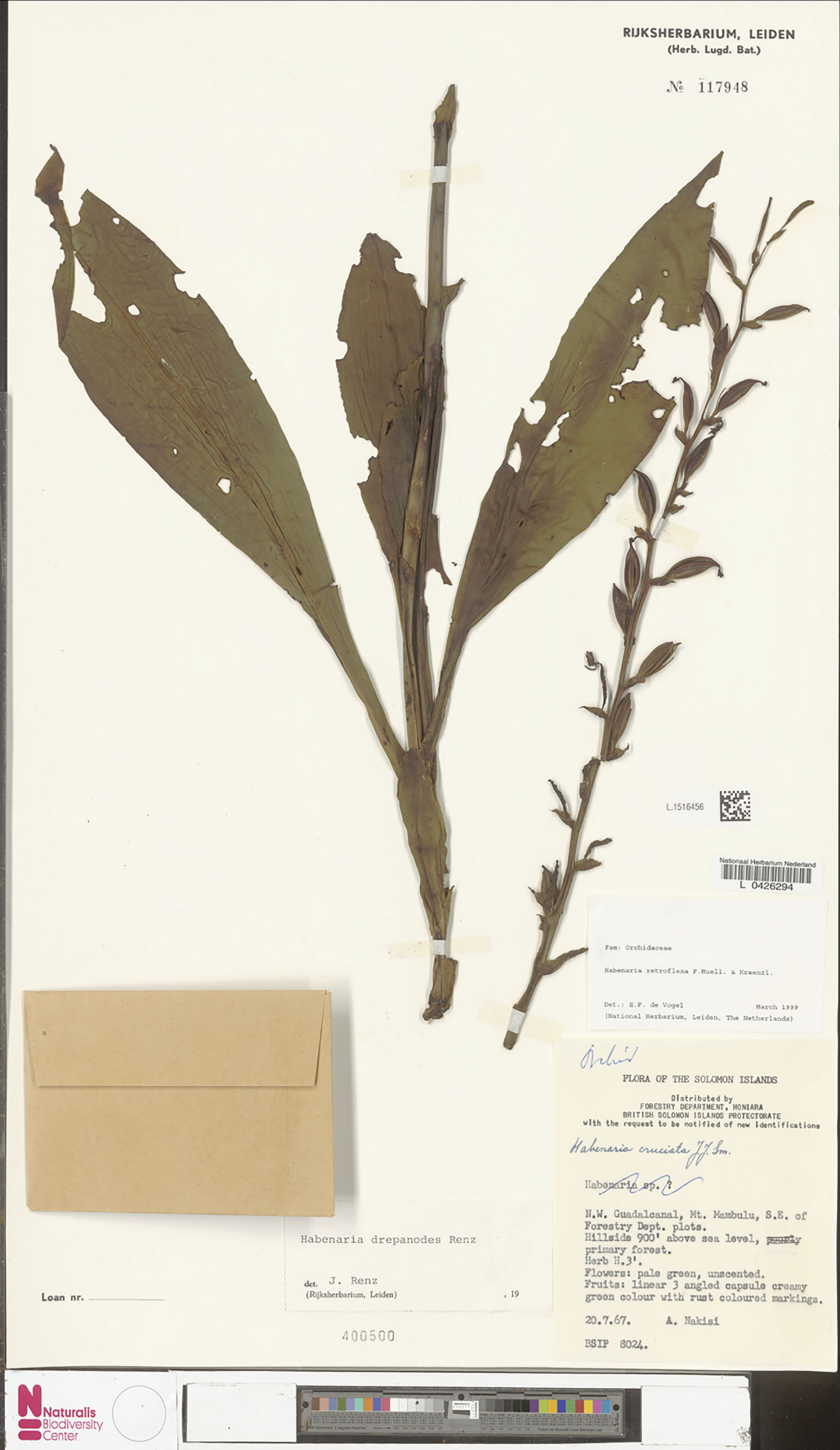
*Habenaria drepanodes* Renz *ex* Kolan., S.Nowak & Szlach., type specimen at Naturalis Biodiversity Center, L.1516456. (CC0 1.0; https://data.biodiversitydata.nl/naturalis/specimen/L.1516456).

**Figure 33:**
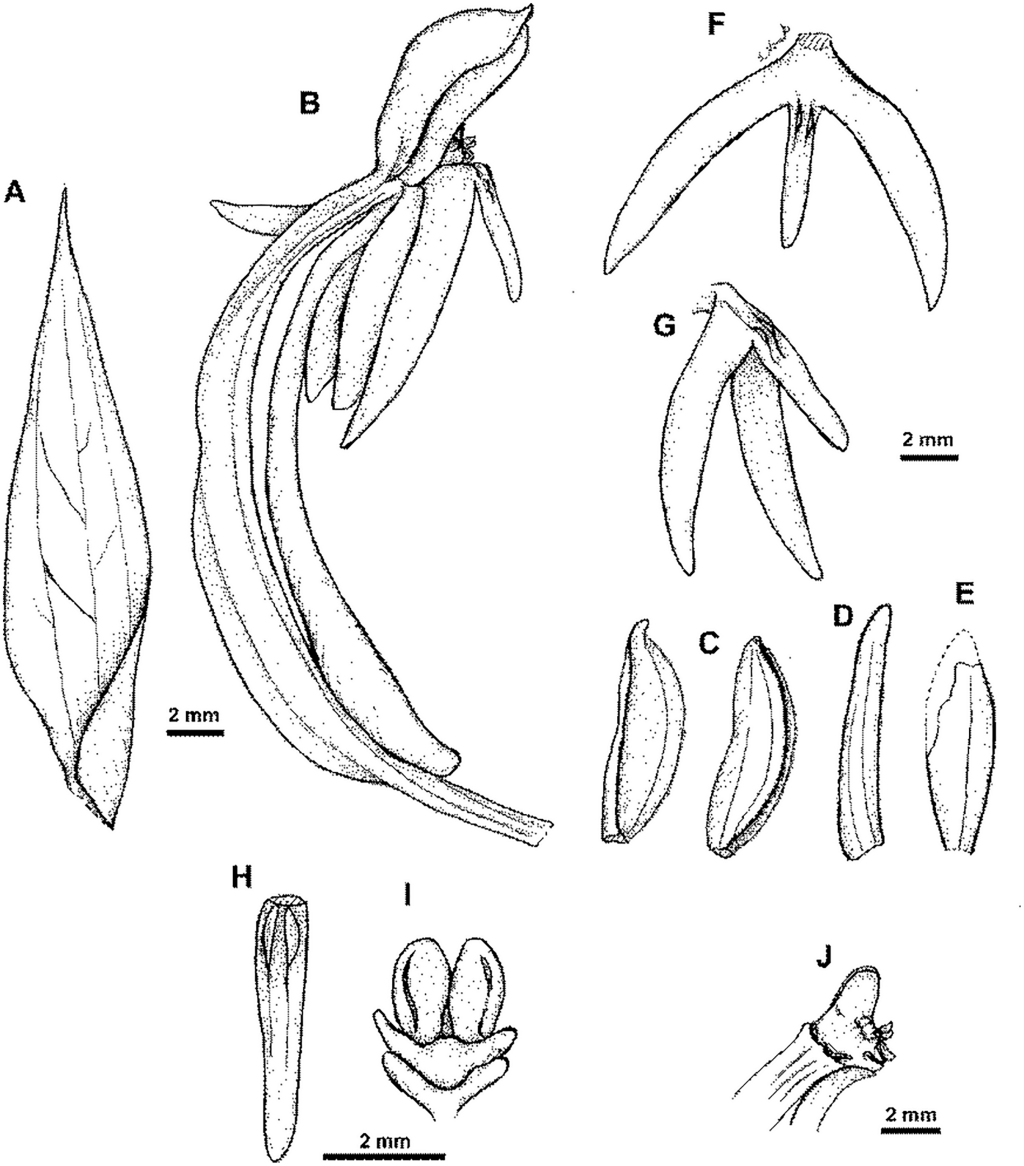
*Habenaria drepanodes*: (A) bract; (B) flower; (C) dorsal sepal; (D) petal; (E) lateral sepal; (F) lip, front view; (G) lip, side view; (H) lip middle lobe; (I) & (J)—gynostemium (*Nakisi BSIP8024*; LAE).

Plant about 90.0 cm tall. Leaves 3, basal, blade up to 20.0 cm long, 5.0 cm wide, narrowly elliptic to ovate-elliptic, subacute, basally narrowing into canaliculated petiole clasping stem; petiole up to 9.0 cm long. Raceme about 50.0 cm long, laxly many-flowered. Flowers pale green. Floral bract 14.0–22.0 mm long, 3.0–8.0 mm wide, usually subequalling ovary and pedicel in length, broadly lanceolate, ciliate along margins. Ovary with pedicel 11.0–28.0 mm long, 3-ribbed, ciliate along ribs. Dorsal sepal 8.0 mm long, 4.5 mm wide, concave-cucullate, ovate-elliptic, obtuse, 3-veined, ciliate along margins and externally along 3 thickened ribs. Lateral sepals 8.0 mm long, 3.0 mm wide, obliquely elliptic, obtuse, 1-veined, ciliate along margins. Petal 8.0 mm long, 1.8 mm wide, oblong-lanceolate, rounded at apex, 2-veined, ciliate along margins. Lip fleshy, 3-lobed above basal 1.9 mm; middle lobe 4.5 mm long, 1.0 mm wide, oblong-ligulate, obtuse, with two elevated plates at base; lateral lobes 9.0 mm long, 2.0 mm wide, falcate, oblong, subacute. Spur 23.0 mm long, filiform in basal third, swollen above. Gynostemium 3.0 mm long, short and massive. Anther erect, 2-chambered, chambers parallel, elongated at base into short antherophores. Auriculae small. Stigma bilobed, both lobes forming stigmaphores 1.2 mm long. Rostellum 3-lobed, middle lobe concave, obscure, adnate to ventral surface of connective, lateral lobes short, fused with antherophores. Figs. 32–33.

*Habitat:* Primary forest. Alt. 900 m.

*Distribution*: Known exclusively from the Solomon Islands.

*Representative specimen*: Solomon Islands. NW Guadalcanal, Mt. Mam-bulu, S.E. of Forestry Dept. plots. 20 Jul 1967 *A. Nakisi BSIP8024* (K!, L!, LAE!; RENZ!—photo and drawing). Fig. 31.

*Notes: Habenaria drepanodes* resembles *H. retroflexa*, but the two species may be easily distinguished based on the lip shape. In *H. drepanodes*, the middle lobe is half as long as the lateral ones and it is ornamented with two elevated plates. In *H. retroflexa*, the lip lobes are nearly equal in length and there is no ornamentation on the middle lobe. An additional difference between the species is the ratio between ovary and floral bract length. In *H. drepanodes*, the floral bracts are broadly lanceolate and nearly equal in length to the ovary and pedicel. In *H. retroflexa*, the floral bracts are broadly ovate, usually half the length of the ovary with pedicel, or shorter; rarely the bracts in the basal part of the raceme are longer.

2.3.5. ***Habenaria ensigera*** Renz, Pl. Syst. Evol. 155(14): 319. 1987. TYPE: Indonesia. *P. van Royen 5306* (Holotype: K!; Isotypes: A!, L!, LAE!, RENZ!).

**Figure 34:**
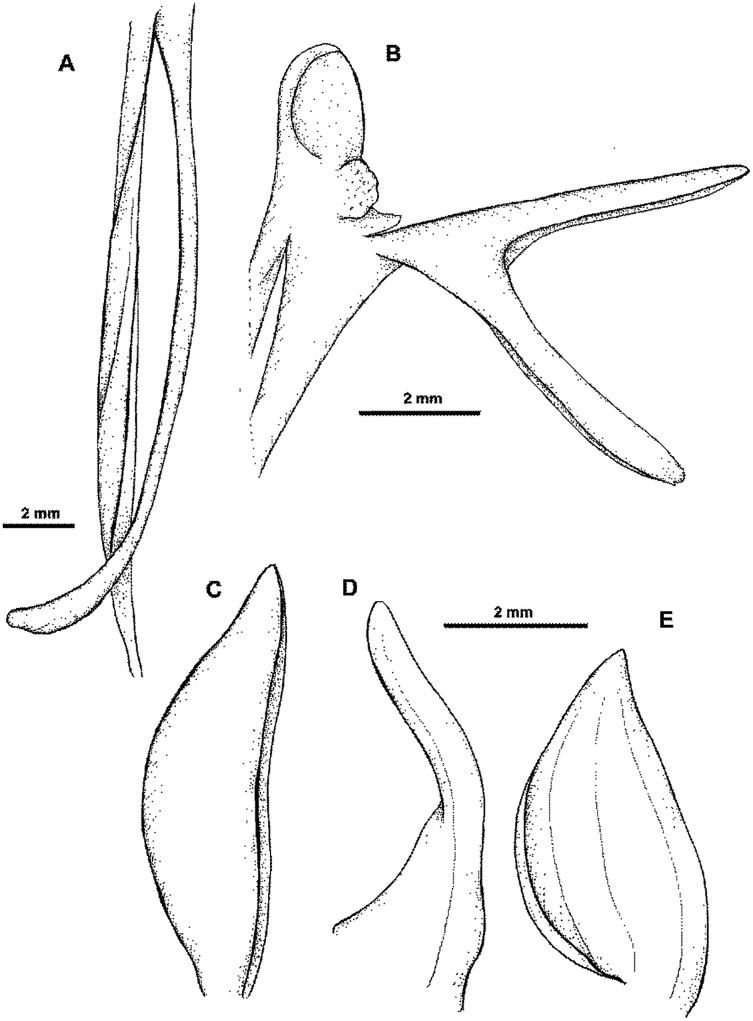
*Habenaria ensigera* Renz, type specimen at Naturalis Biodiversity Center, L 0061406. (CC0 1.0; https://data.biodiversitydata.nl/naturalis/specimen/L%20%200061406).

**Figure 35:**
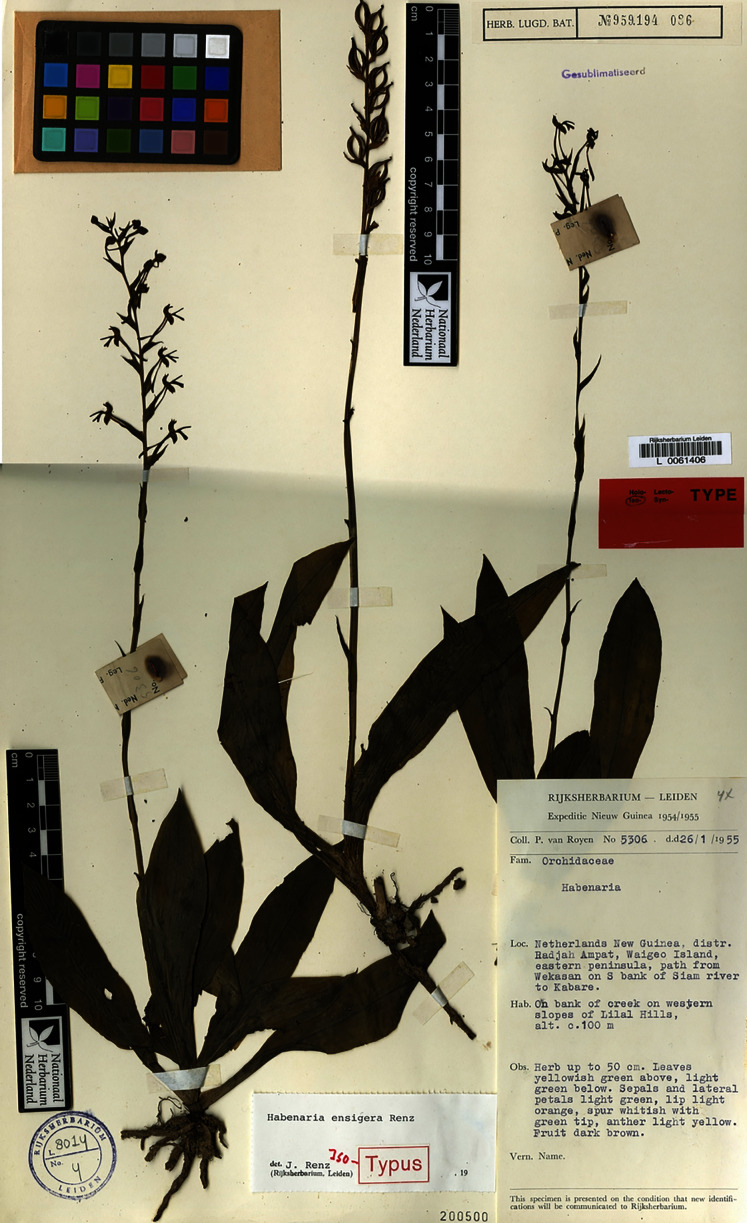
*Habenaria ensigera* Renz: (A) ovary and spur; (B) lip with gynostemium; (C) dorsal sepal; (D) petal; (E) lateral sepal (drawn from *van Royen 5306*; L).

Plant about 50.0 cm tall, leaves gathered in the basal rosette. Leaves 4–6, up to 15.0 cm long, 3.0 cm wide, narrowly obovate-oblanceolate to oblanceolate, apex acute to acuminate, base petiolate. Inflorescence racemose; peduncle 40.0 cm long, peduncle-scales lanceolate, acuminate; rachis 10.0 cm long, rather laxly many-flowered. Floral bract about 7.0–9.0 mm long, ovate, shortly acuminate. Pedicel with ovary 15.0–20.0 mm long, slightly 6-ribbed, ribs minutely ciliate. Flowers light green with light orange lip. Dorsal sepal 5.0–5.5 mm long, 4.0 mm wide, concave, ovate, apex uncinate, externally keeled along the mid-vein, the keel very shortly ciliate, 3-veined. Lateral sepals 5.0–5.5 mm long, 2.5–3.0 mm wide, deflexed, obliquely oblong ovate, subacute, 3-veined. Petals 5.0–5.5 mm long, 1.0–1.2 mm wide, linear, slightly widened along the anterior margin at the base. Lip 3-lobed above short claw; middle lobe 4.0–4.5 mm long, 0.5–0.7 mm wide, ligulate, obtuse, somewhat carnose, almost triangular in cross-section, slightly curved; lateral lobes 4.5 mm long, 1.0–1.2 mm wide, narrowly rectangular, obliquely truncate at the apex, shortly and irregularly bilobulate-emarginate. Spur 17.0 mm long, narrowly cylindrical, acute. Gynostemium 2.0 mm long, stigmaphores slightly shorter than anther channels which are 0.8 mm long. Figs. 34–35.

*Habitat:* On creek bank. Alt. 100 m.

*Distribution*: New Guinea.

*Representative specimen*: New Guinea (Indonesia. Prov. Papua). Irian Jaya. Distr. Radjah Ampat, Waigeo Island, eastern Peninsula, path from Wekasan on S bank of Siam river to Kabare. On bank of creek on western slopes of Lilal Hills. Alt. 100 m. 26 Jan 1955. *P. van Royen 5306* (A!, K!, L!, LAE!, RENZ!). Fig. 31.

*Notes: Habenaria ensigera* has unique shortly and irregularly bilobulate-emarginate lateral lobes of the lip, not found in any other species of this group.

The following description of the colour of the flowers is given by van Royen on the label of the Herbarium sheet: “sepals and lateral petals light green, lip light orange, spur whitish with green tip, anther light yellow” (Renz, 1987).

2.3.6. ***Habenaria rechingeri*** Renz, Pl. Syst. Evol. 155(14): 324. 1987. TYPE: Indonesia. *P. van Royen 5155* (Holotype: L!, Isotypes: AMES!, RENZ!).

**Figure 36:**
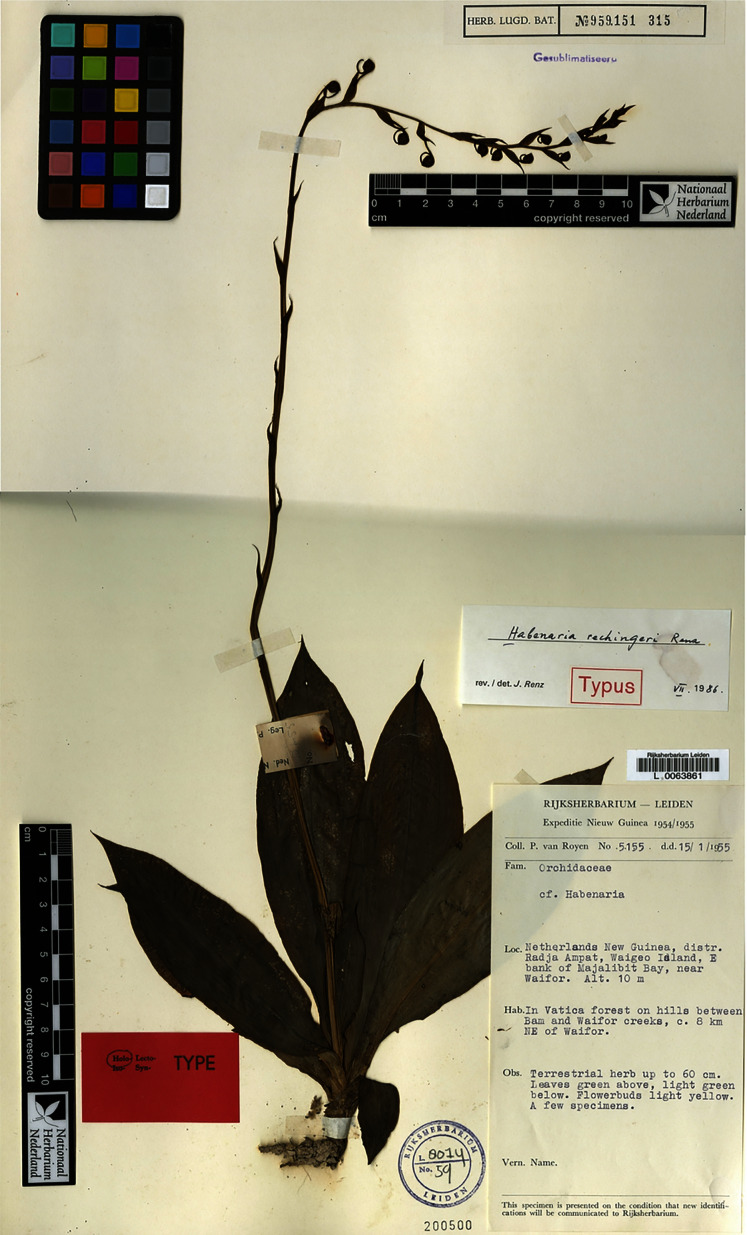
*Habenaria rechingeri* Renz, type specimen at Naturalis Biodiversity Center, L 0063861. (CC0 1.0; https://data.biodiversitydata.nl/naturalis/specimen/L%20%200063861).

**Figure 37:**
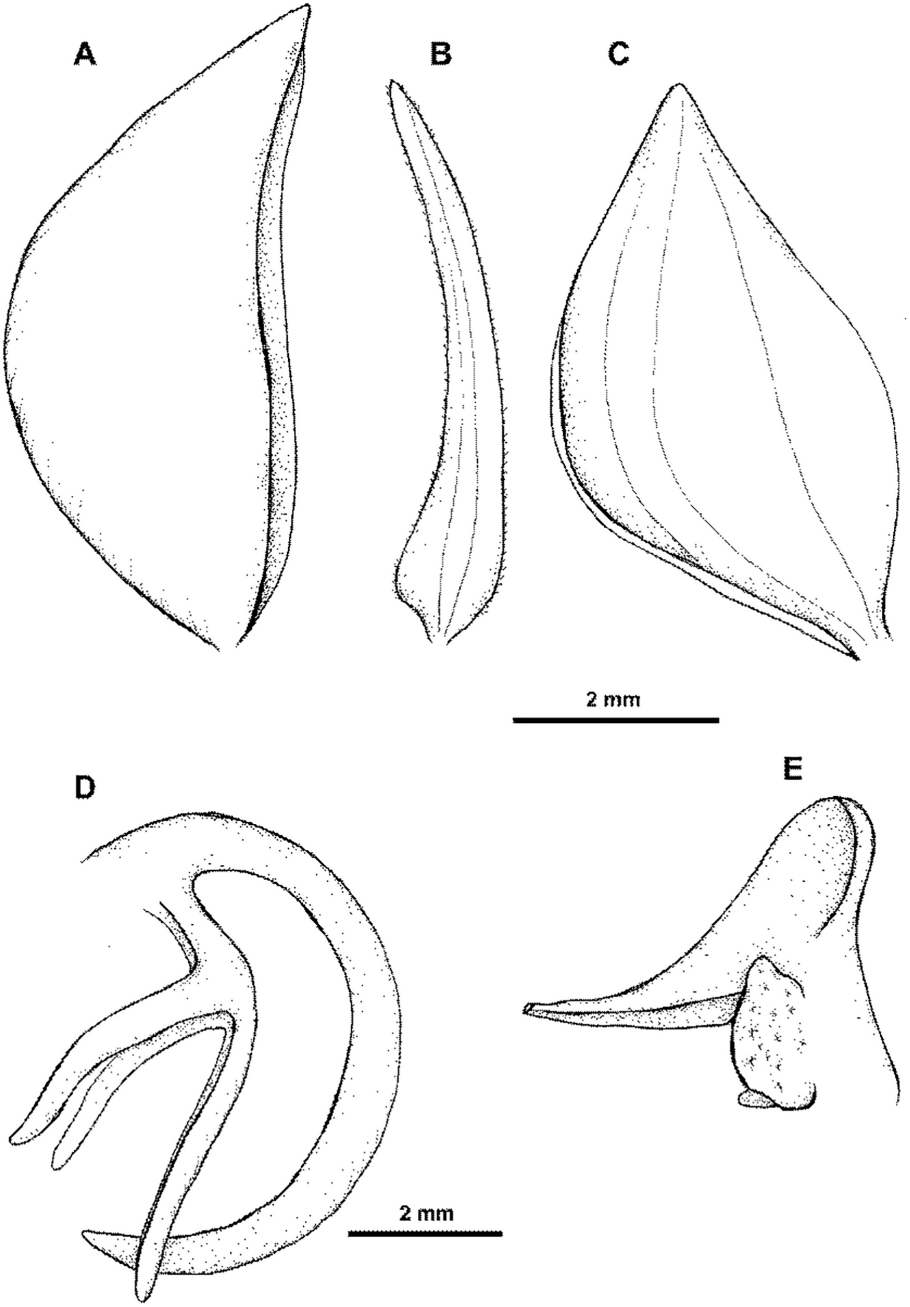
*Habenaria rechingeri* Renz: (A) dorsal sepal; (B) petal; (C) lateral sepal (D) lip with spur; (E) gynostemium (drawn from *van Royen 5155*; L).

Plant up to 110.0 cm tall, leafy at the base. Leaves 4–, erect-patent, up to 20.0 cm long, 5.0 cm wide, elliptic-oblanceolate, narrowed at the base, apex acute to shortly acuminate. Inflorescence 30.0 cm long, peduncle-scales distant, acuminate; rachis 15.0–20.0 cm long, laxly many-flowered. Floral bracts almost as long as the ovary, ovate-lanceolate, acuminate. Pedicel with ovary 17.0–20.0 mm long, slightly keeled, keels very minutely ciliate. Flowers light yellow or greenish-yellow. Dorsal sepal 6.0–6.5 mm long, up to 5.0 mm wide, cucullate, broadly ovate, acute, 3-veined, mid-vein very minutely ciliate on the outside. Lateral sepals 6.0 mm long, 3.5 mm wide, deflexed, obliquely ovate-elliptic, obtuse, 3-veined. Petals 5.5–6.0 mm long, 1.2–1.5 mm wide, oblong-lanceolate, somewhat falcate, obtuse, 2-veined, margins very shortly ciliate. Lip 3-lobed just above the base, fleshy, almost cruciform when spread; middle lobe 4.5–5.0 mm long, linear-lanceolate, obtuse, margins more or less involute; lateral lobes 4.0–4.7 mm long, linear-lanceolate, obtuse. Spur 15.0–17.0 mm long, strongly incurved, narrowly cylindrical, slightly dilated at the apex, acute. Gynostemium 3 mm long, stigmaphores very short, anther channels elongated, 3.0 mm long. Figs. 36–37.

*Habitat:* Growing in forest on hills and in forest dominated by Sapotaceae. Alt. 10–400 m.

*Distribution*: New Guinea.

**Figure 38:**
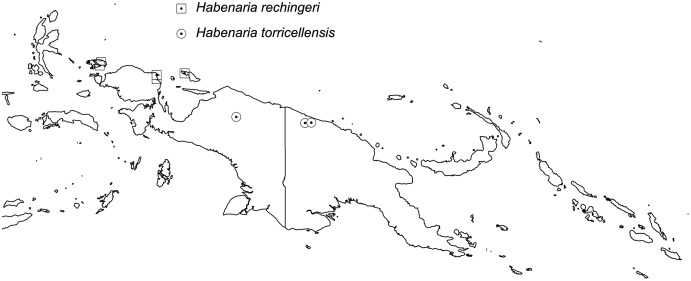
Distribution of *Habenaria rechingeri* and *H. torricellensis*.. Base map downloaded from www.naturalearthdata.com.

*Notes: Habenaria rechingeri* can be rather easily distinguished by having a minutely ciliate ovary combined with a pair of basal lip calli. It is noteworthy that it has one of the smallest flowers in New Guinean *Habenaria*.

*Representative specimens*: (Indonesia. Prov. Papua). Jaya. Distr. Radya Ampat. Waigeo Island. E bank of Majalibit Bay. Near Waifor in Vatica forest on hills between Bam and Waifor creeks. Alt. 10 m. 15 Jan 1955. *P. van Royen 5155* (AMES!, L!, RENZ!); Selogof on E bank of Majalibit Bay, in Sapotaceae dominated forest on limestone cliffs behind the village. Alt. 20 m. 21 Jan 1955. *P. van Royen 5241* (AMES!, K!, L!, RENZ!); North East Kepala Burung, Irian Jaya. Kapubaten Manokwari. Kecamatan Manokwari. Between Sungai Acemo and Gunung Borai, c. 1 km from the coast. Alt. 75 m. 11 Apr 1994. *M.J. Sands & al. 6158* (K!); North East Kepala Burung, Irian Jaya. Kecamatan Manikwari. Mupi Dessa. Arfak Mts., Mupi Valley system. On trail from Mupi to Humeibou, near base camp. 18 Apr 1995. *U.W. Mayar & al. 443* (K!); North East Kepala Burung, Irian Jaya. Kecamatan Sawe. Sawe subdistrikct. Andai Forest reserve, along the Jalan Umboi. Alt. c. 300 m. 22 Apr 1995. *U.W. Mayar & al. 450* (L!, K!). Fig. 38.

*H. rechingeri* resembles the parapatric *H. torricellensis* which, however, has much larger flowers (sepals 6–6.5 *vs* 9.5–12.5 mm). Tepals and lip lobes of *H. rechingeri* are shorter than 6 mm, whereas in *H. torricellensis* they are usually longer than 10 mm.

2.3.7. ***Habenaria retroflexa*** F. Muell. & Kraenzl. *ex* Kraenzl., Bot. Jahrb. Syst. 17: 488. 1893. TYPE: New Guinea. *Sine coll. ex* MEL (B†).

Plant up to 70.0 cm tall, leaves basal or scattered along basal half of the stem. Leaves 20.0–30.0 cm long, 4.5–7.0 cm wide, oblanceolate to obovate, acute to acuminate, narrowed at the base; petiole up to 9.0 cm long, sheathing at the base. Inflorescence up to 55.0 cm long, rather laxly many-flowered. Floral bracts 15.0–22.0 mm long, ovate-lanceolate, acute. Pedicel with ovary 18.0–30.0 mm long, 3-winged, narrowly cylindrical. Flowers green. Dorsal sepal 7.0–8.0 mm long, 3.5–4.5 mm wide, concave, broadly ovate, obtuse, 3-veined. Lateral sepals 7.5–8.0 mm long, 3.0–3.3 mm wide, obliquely broadly ovate, obtuse, 3-veined. Petals 7.0–8.0 mm long, 1.0–1.8 mm wide, linear, somewhat extended at the base, obtuse, 2-veined. Lip 3-lobed above 1 mm long claw; middle lobe 4.5–7 mm long, 0.8–1 mm wide, linear-lanceolate, acute; lateral lobes 6.0–9.0 mm long, 1.0–2.0 mm wide, linear, divergent or falcate, obtuse. Spur 14.0–23.0 mm long, cylindrical-filiform, acute. Gynostemium about 3.0 mm long.

*Habitat:* Terrestrial in deep leaf litter in rain forest. Alt. 150–300 m.

*Distribution*: New Guinea, Solomon Islands.

*Representative specimen*: New Guinea. In deep leaf litter in rain forest. Alt. 150-300 m. *Sine coll. ex* MEL (B†).

*Notes:* The species description presented above is based on work published by Lewis & Cribb (1991). This is the only species of New Guinean *Habenaria* with lip ornamented by a pair of basal, rather obscure calli. Unlike in the very similar *H. rechingeri* its ovary is glabrous.

2.3.8. ***Habenaria torricellensis*** Schltr., Fl. Schutzgeb. Südsee: 80. 1905. TYPE: Papua New Guinea (Deutsch Neu-Guinea). *R. Schlechter 14323* (B†); *T.M. Reeve 565* (Neotype, *designated here*: LAE!, Isoneotypes: K!, RENZ!).

**Figure 39:**
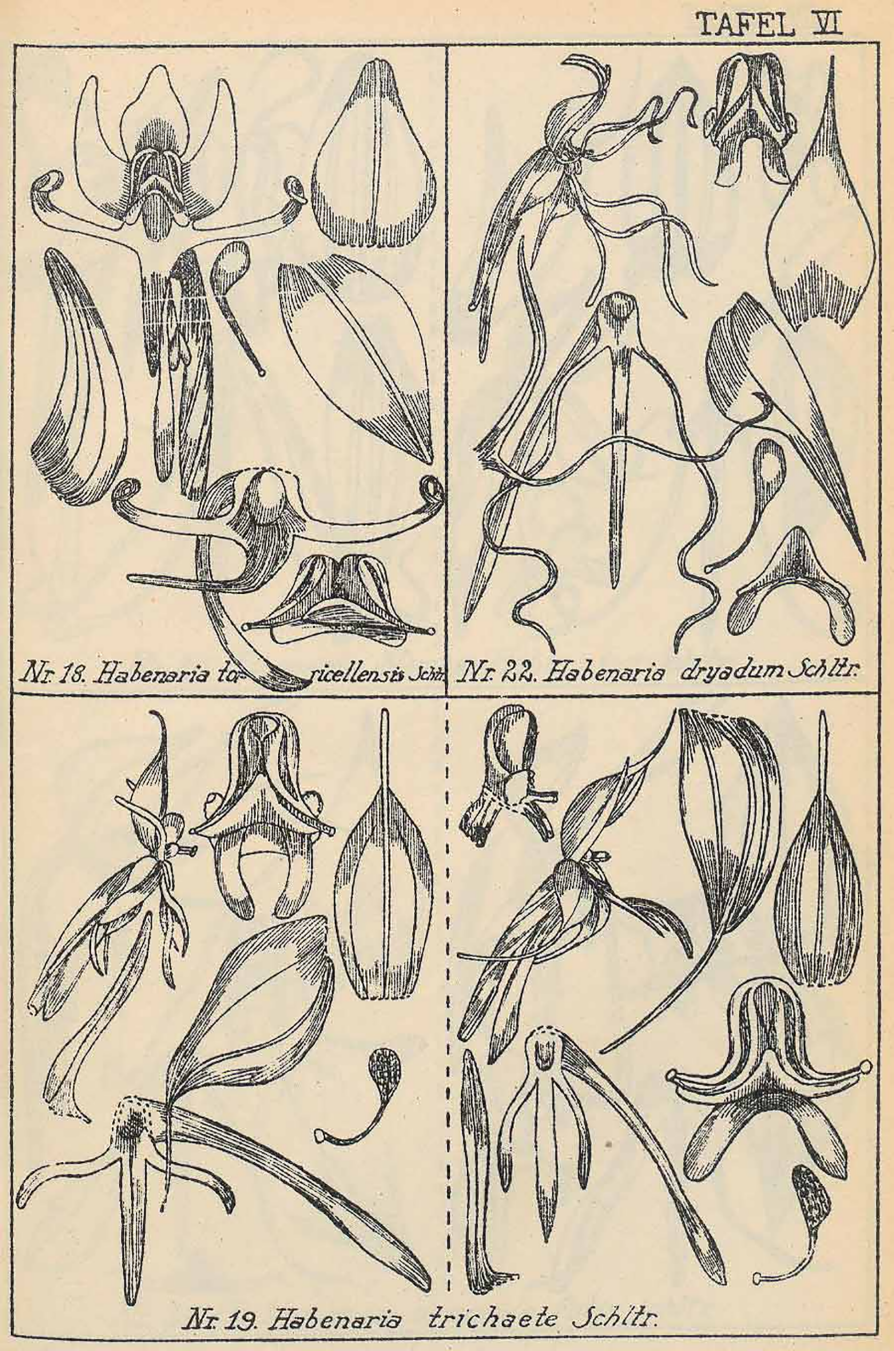
*Habenaria torricellensis* Schltr., *H*. *dryadum* Schltr.,*H. trichaete* Schltr.—drawings from Figurenatlas zu den Orchidaceen von Deutsch-Neu-Guinea, Feddes Repert. Spec. Nov. Regni Veg. Beih. 21(1).1923. (URL: https://www.biodiversitylibrary.org/page/57688231).

**Figure 40:**
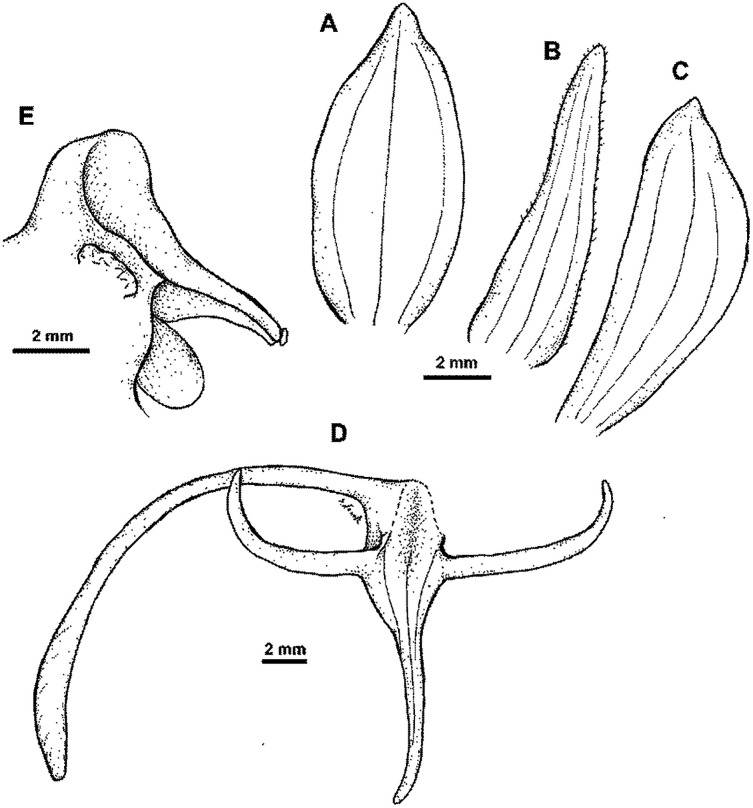
*Habenaria torricellensis* Schltr.: (A) dorsal sepal; (B) petal; (C) lateral sepal (D) lip with spur; (E) gynostemium (drawn from *Brass 13761*; AMES).

Plant 50–90.0 cm tall, leafy in the basal third. Leaves 2–5, erect-patent, 22.0–30.0 cm long, 3.0–5.0 cm wide, oblanceolate, acute to acuminate. Sterile bracts 2–3, up to 3.5 cm long, lanceolate, acuminate. Inflorescences 40.0–60.0 cm long, laxly up to 15-flowered. Floral bracts 13.0–18.0 mm long, ovate, acuminate. Pedicel with ovary 28.0–40.0 mm long, narrowly cylindrical. Flowers green. Dorsal sepal 9.5–11.0 mm long, 2.5–5.0 mm wide, concave, ovate to elliptic-ovate, apex obtuse, 3-veined. Lateral sepals 10.5–12.5 mm long, 3.6–5 mm wide, obliquely obovate to obovate-oblanceolate, apex obtuse, 3-veined. Petals entire, 10.0–12.0 mm long, 2.2–3.2 mm wide, ligulate-lanceolate, slightly oblique, apex obtuse, margins ciliate, 3-veined. Lip 3-lobed just above the base or entire; middle lobe (or whole lip if lateral lobes absent) 11.0–12.0 mm long, up to 3.0 mm wide, linear-ligulate, recurved, apex obtuse; lateral lobes (if present) 11.0–13.0 mm long, 1.3 mm wide, linear-filiform, divergent, apically upcurved. Spur 19.0–25.0 mm long, narrowly cylindrical, slightly swollen in apical third, blunt. Gynostemium up to 4.0 mm long; stigmaphores almost half as long as anther channels. Figs. 39–40.

*Habitat:* Terrestrial in rainforest, occasional on muddy banks of a stream. Alt. 700 m.

*Distribution*: New Guinea.

*Representative specimens*: Papua New Guinea. Torricelli Mts. Alt. 700 m. 18 Apr 1902. *R. Schlechter 14323* (B†); Lumi District, West Sepik Province, Sibilanga (Torricelli Mts.), East Palei. Alt. 700 m. Aug 1981. *T.M. Reeve 565* (K!, LAE!, RENZ!); Indonesia. Papua Prov. Bernhard Camp, Idenburg River. Apr 1980. *L.J. Brass 13761* (AMES!, BO!). Fig. 38.

*Notes: Habenaria torricellensis* has somewhat larger flowers than other species of this group, *i.e. H. bougainvilleae*, *H. retroflexa* and *H. rechingeri*. Its sepals are more than 9.5 mm long, whereas in the aforementioned species they are less than 9 mm long. Additionally, the lateral sepals of *H. torricellensis* and *H. bougainvilleae* are obliquely obovate to obovate-oblanceolate or obovate-triangular, widest above the middle, but in the latter the upper margin of lateral sepals is minutely ciliolate.

Original material for *H. torricellensis* does not exist today to our knowledge. The taxon belongs to a morphologically difficult group of few similar species. Therefore we decided to designate specimen of *Reeve 565* as a neotype (LAE), which agrees with the original description and has duplicates (K and RENZ) to increase its accessibility.

3. ***Habenaria dracaenifolia*-group**

Leaves gathered near the middle of the stem. Petals deeply divided into two segments, both segments entire, linear to filiform, lip lobes entire, filiform to linear. Gynostemium relatively short and massive, anther oblong ellipsoid, stigmaphores and rostellophores short, filiform, stigmaphores long, ligulate, auricles very large, prominent.

### Key to species of *Habenaria dracaenifolia*-group

1. Tepals and lip papillose-tomentose 3.8. ***H. trichoglossa***
1* Tepals and lip glabrous 2
2. One of petal lobe reduced to a tooth-like appendage 3
2* Petal lobes linear, filiform 4
3. Floral bracts acuminate, petals anterior lobe 13–20 mm long, filiform with a bristle-like apex, acuminate, posterior lobe 1–2 mm long, dentate, obtuse, stigmaphores distinctly longer than the anther channels 3.7. ***H. trichaete***
3* Floral bracts aristate, petals anterior lobe ca 2.5 mm, narrowly falcate, posterior lobe 10–13 mm long, 1linear, acuminate, stigmaphores about as long as anther channels 3.6. ***H. stenopetala***
4. Spur subequal or longer than pedicellate ovary 5
4* Spur shorter than pedicellate ovary 6
5. Lip middle lobe 13–17 mm long, narrowly linear, lateral lobes 19–20 mm long, spur 20–23 mm long, cylindrical, slightly swollen in the apical half 3.2. ***H. dryadum***
5* Lip middle lobe 20 mm long, filiform, longer than lateral lobes, spur 25 mm long, slightly sigmoid, in basal 2/3 filiform, in the apical 1/3 abruptly inflated, with a dorsal groove when dried 3.3. ***H. micholitziana***
6. Lip middle lobe shorter than lateral lobes 7
6* Lip middle lobe longer than lateral lobes 3.4. ***H. devogeliana***
7. Stigmaphores as long as the anther channels, ascending, lip middle lobe 6–7 mm long, lateral lobes about 12 mm long 3.5. ***H. novae-hiberniae***
7* Stigmaphores slightly longer than the anther channels, parallel to stigmaphores, middle lobe 7–22 mm long, lateral lobes 12–23 mm long 3.1. ***H. dracaenifolia***

3.1. ***Habenaria dracaenifolia*** Schltr., Fl. Schutzgeb. Südsee.: 77. 1905. TYPE: Papua New Guinea (Deutsch Neu-Guinea). *R. Schlechter 14336* (B†).

= *Habenaria dracaenifolia* var. *laxa* Schltr., Repert. Spec. Nov. Regni Veg. Beih. **1**: 15. 1911. TYPE: Papua New Guinea (Deutsch Neu-Guinea). *R. Schlechter 17946* (B†).

**Figure 41:**
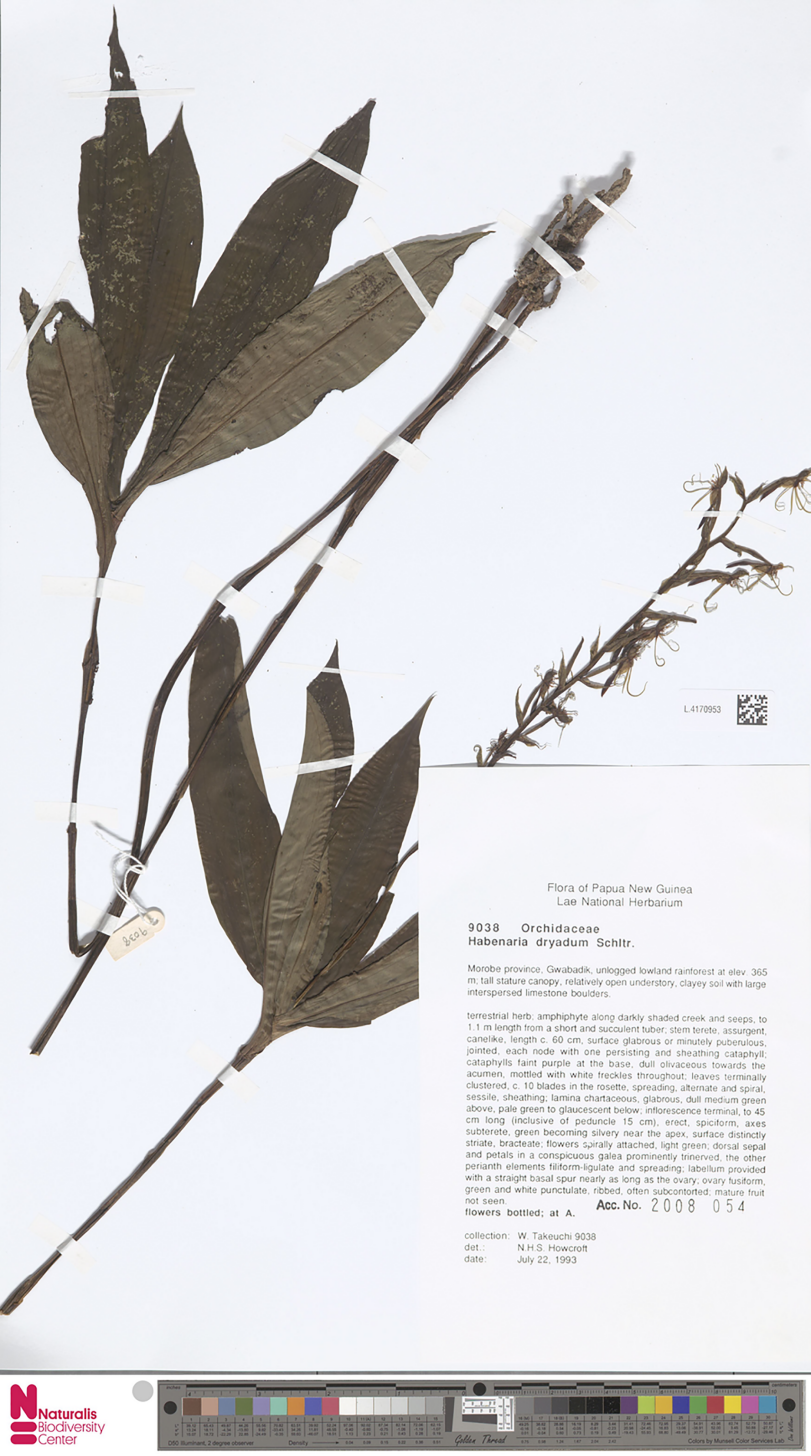
*Habenaria dracaenifolia* Schltr.: herbarium specimen at Naturalis Biodiversity Center, L.4170953. (CC0 1.0; https://data.biodiversitydata.nl/naturalis/specimen/L.4170953).

**Figure 42:**
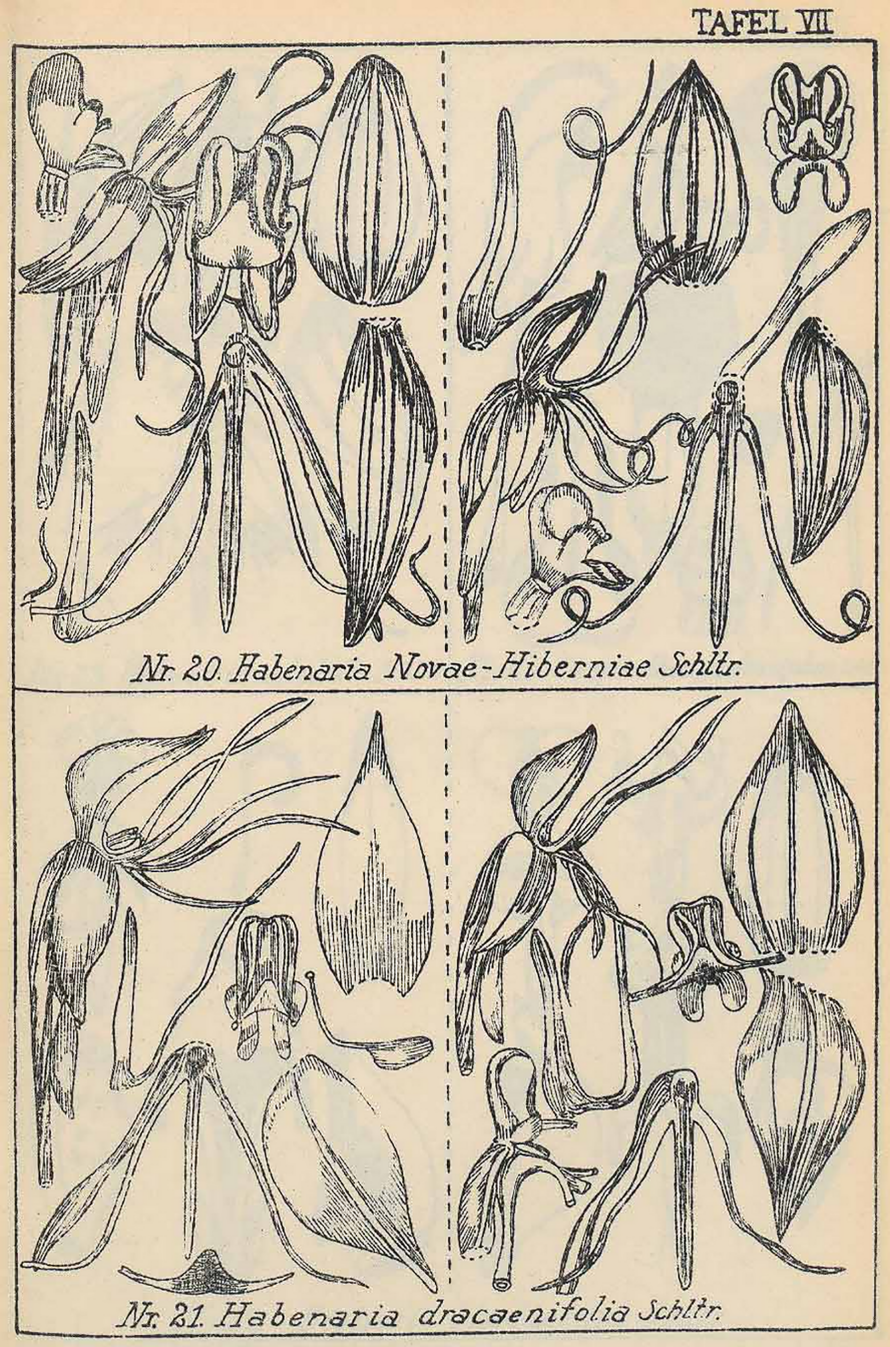
*Habenaria dracaenifolia* Schltr. and *H*. *novae-hiberniae* Schltr.—drawings from Figurenatlas zu den Orchidaceen von Deutsch-Neu-Guinea, Feddes Repert. Spec. Nov. Regni Veg. Beih. 21(1).1923 (Plate VII). URL: https://www.biodiversitylibrary.org/page/5768822.

**Figure 43:**
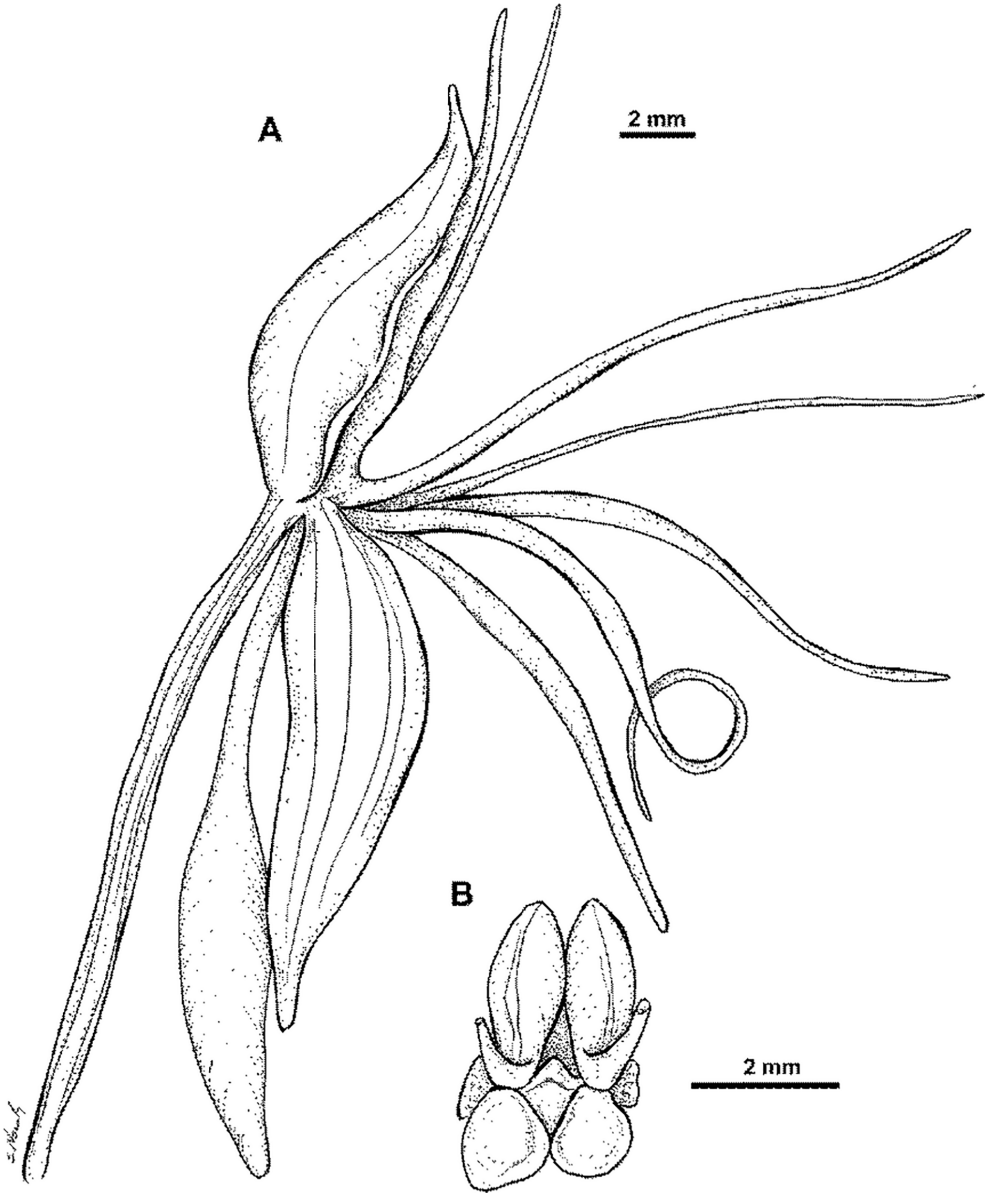
*Habenaria dracaenifolia* Schltr.: (A) flower; (B) gynostemium (drawn from *Takeuchi 9057*; AMES).

Plant up to 110.0 cm long, leafless in basal part. Leaves several, above the middle part of the stem, arranged in a rosette, erect-patent to patent, 15.0–20.0 cm long, 3.0–3.5 cm wide, lanceolate, acuminate. Inflorescence up to about 25.0 cm long; rachis 15.0–22.0 cm long, rather densely several to many-flowered. Floral bracts up to 30.0 mm long, lanceolate, acuminate. Pedicel with ovary 21.0–25.0 mm long, very slender, fusiform. Flowers green or creamy green. Dorsal sepal 11.0–12.0 mm long, 5.0 mm wide, concave, ovate, acuminate, subobtuse, 3-veined. Lateral sepals 12.0 mm long, 4.0–6.0 mm wide, deflexed, obliquely ovate, acuminate, subobtuse, 4-veined. Petals deeply bilobed; anterior lobe 22.0–23.0 mm long, filiform, subulate, 1-veined; posterior lobe 12.0–14.0 mm long, 0.9 mm wide, linear, long-acuminate, 1-veined. Lip 3-lobed just above the base; middle lobe 7.0–22.0 mm long, narrowly linear, acute; lateral lobes 12.0–23.0 mm long, linear in the lower part, filiform above. Spur 16.0–18.0 mm long, narrowly cylindrical, swollen in the apical part, attenuate towards subobtuse apex. Gynostemium 3.5–4.0 mm long, stigmaphores slightly longer than the anther channels, auricles prominent. Figs. 41–43.

*Habitat:* Terrestrial in leaf-litter in lowland rainforest. Alt. 50–800 m.

*Distribution*: New Guinea.

**Figure 44:**
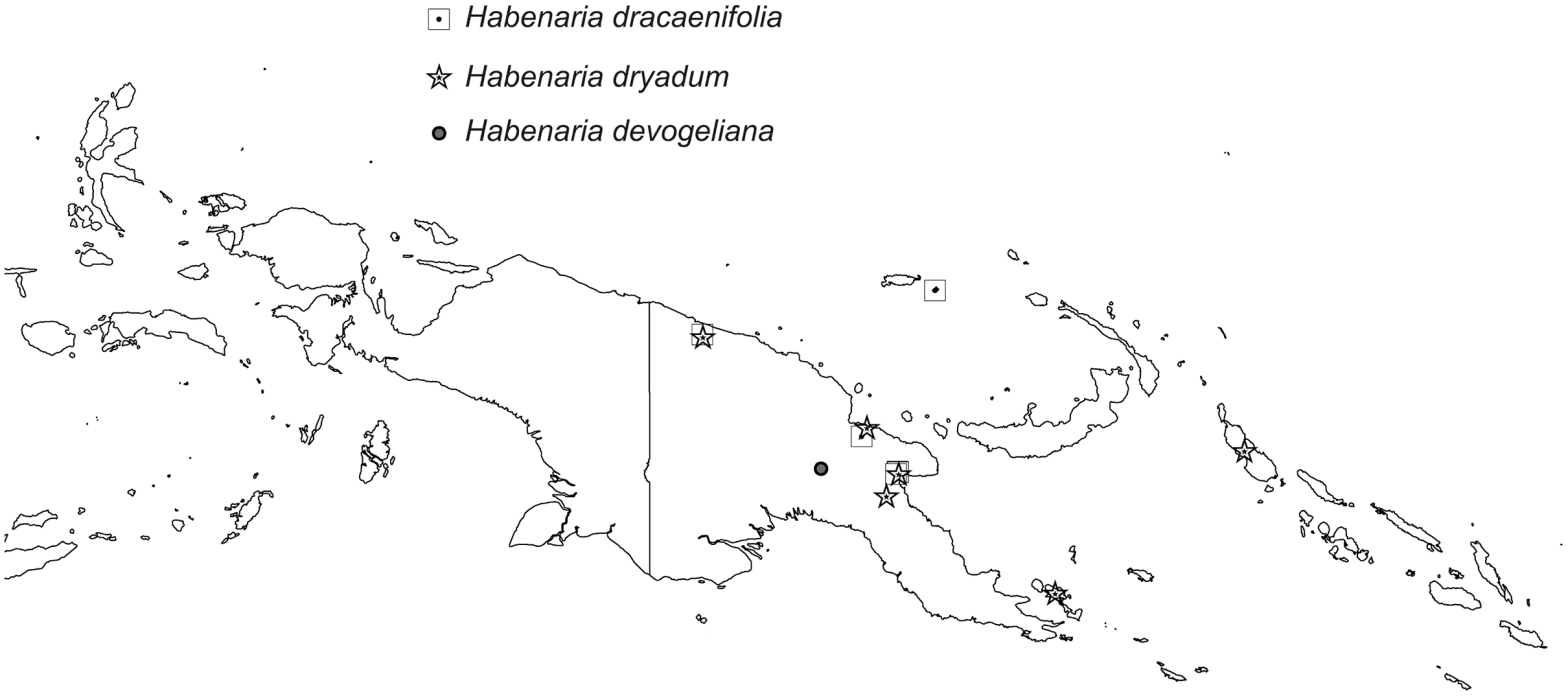
Distribution of *Habenaria dracaenifolia, H. dryadum* and *H*. *devogeliana*.. Base map downloaded from www.naturalearthdata.com.

*Notes: Habenaria dracaenifolia* can be distinguished from *H. novae-hiberniae* based on the length of stigmaphores which are slightly longer than the anther channels (*vs* equally long in *H. novae-hiberniae*). Moreover, the anther channels of *H. novae-hiberniae* are ascending (*vs*. parallel to stigmaphores). The flowers of this species are green or creamy green, whereas those of *H. novae-hiberniae* are greenish-yellow. When describing both, *H. dracaenifolia* and *H. novae-hiberniae*, Schlechter probably considered them different also in flower size (dorsal sepal 6 mm long in *H. novae-hiberniae* *vs* 12 mm long in *H. dracaenifolia* according to original descriptions), but in the course of our study we found this character to be variable and it cannot be used as a distinguishing trait. Further research, preferably genetic, should be made to evaluate the distinctness of these two species.

*Representative specimen*: Papua New Guinea. Torricelli Mts. In forest. Alt. 800 m. Apr 1902. *R. Schlechter 14336* (B†); Kaiser-Wilhelm-Land. Im Humus der Wälder des Finisterre-gebirges. Alt. ca. 700 m. Jul 1908. *R. Schlechter 17946* (B†); Morobe Prov., Busu valley, N of Lae. Alt. 250 m. 31 July 1979. *J. Renz 12336* (RENZ!); Morobe Province. Atzera Range, inland from the 7–8 mile settlement. Alt. 200–300 m. 2 Aug 1993. *W.N. Takeuchi 9057* (AMES!); Morobe Prov., Gwabadik. Alt. 365 m. 22 Jul 1993. *W.N. Takeuchi 9038* (A!, L!); Manus Prov., Peniselu Admin. Centre, Rambutyo Island. Alt. 50 m. *K. Kerenga & al. s.n*. (LAE! 77427); Morobe Distr. Lae subdistrict, Sunkwep logg. road. Alt. 300 ft. 4 Aug 1971. *P. Katik NGF 46792* (LAE!). Fig. 44.

*Habenaria dracaenifolia* and *H. dryadum* are very similar in many respects, and it cannot be excluded that they are conspecific. To the best of our knowledge *H. dracaenifolia* can be separated from *H. dryadum* by acuminate leaves without bristle-like mucro, longer anterior petal lobes and lip lateral lobes, and shorter spur. More extensive studies are necessary to confirm whether they are reliable characters.

3.2. ***Habenaria dryadum*** Schltr., Repert. Sp. Nov. Regni Veg. Beih. 3: 80. 1906. ≡ *Habenaria epiphylla* Schltr., Fl. Schutzgeb. Südsee: 78. 1905, *nom. illeg*. TYPE: Papua New Guinea [Deutsch Neu-Guinea]. *R. Schlechter 14175* (B†).

= *Habenaria dryadum* Schltr. var. *major* Schltr., Repert. Spec. Nov. Regni Veg. Beih. 1: 15. 1911. TYPE: Papua New Guinea (Deutsch Neu-Guinea). *R. Schlechter 15707* (B†).

**Figure 45:**
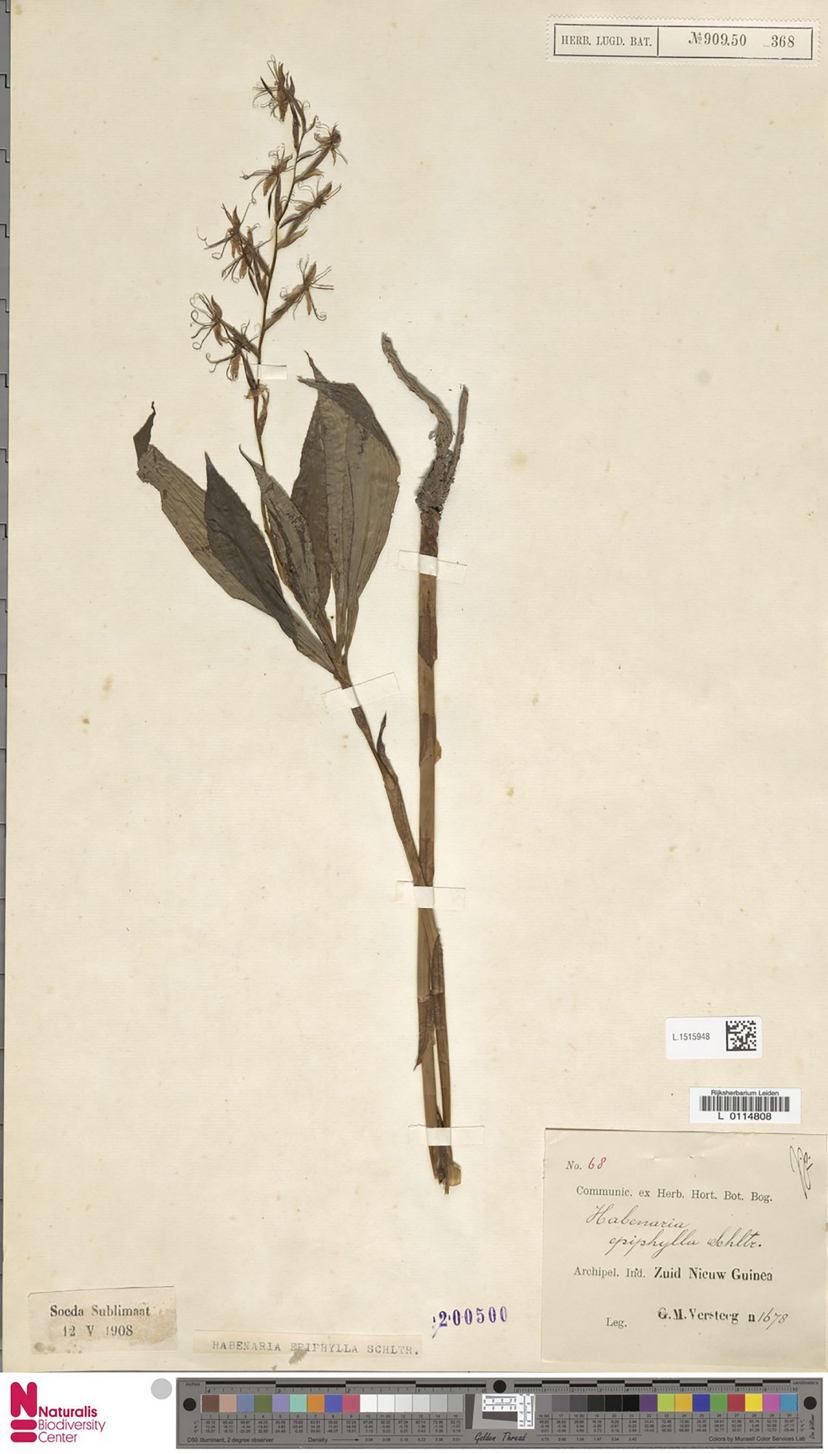
*Habenaria dryadum* Schltr., herbarium specimen at Naturalis Biodiversity Center, L.1515948. (CC0 1.0; https://data.biodiversitydata.nl/naturalis/specimen/L.1515948).

**Figure 46:**
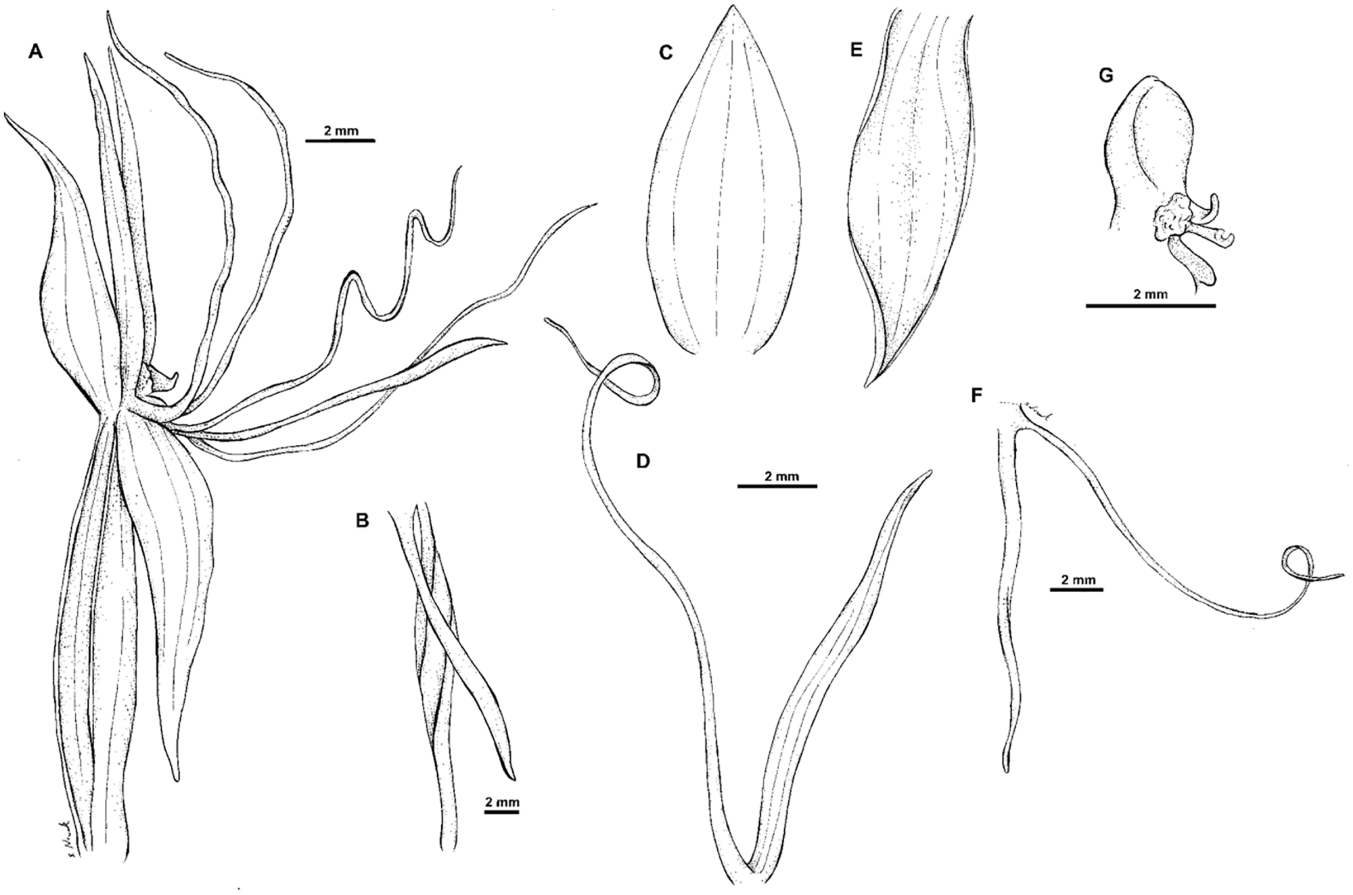
*Habenaria dryadum* Schltr.: (A) flower; (B) ovary and spur; (C) dorsal sepal; (D) petal; (E) lateral sepal; (F) lip; (G) gynostemium. Drawn from *Takeuchi & Ama 21767*; (A) & (G)—AMES; *Versteeg 1678*;(B)–(F)—L.

Plant up to almost 100.0 cm tall, with sheaths in the lower half, leafy above. Leaves 6–10, arranged in a rosette, 12.0–20.0 cm long, 3.0–4.0 cm wide, lanceolate, acuminate, with a bristle-like mucro; above the rosette short-lived, lanceolate, sheaths. Inflorescence 10.0–15.0 cm long, densely many-flowered. Floral bracts about 18.0–22.0 mm long, lanceolate, acuminate. Pedicel with ovary 14.0–20.0 mm long, fusiform. Flowers greenish or greenish-yellow. Dorsal sepal 10.0–11.0 mm long, 4.0–5.0 mm wide, concave, ovate-lanceolate, acuminate, 3-veined. Lateral sepals 12.0 mm long, 4.2 mm wide, deflexed, obliquely ovate-lanceolate, long-acuminate, 3-veined. Petals bilobed to the base; anterior lobe 15.0–18.0 mm long, filiform, 1-veined; posterior lobe 12.0 mm long, 0.8 mm wide, narrowly linear-falcate, acute, slightly falcate, 1-veined. Lip 3-lobed just above the base; middle lobe 13.0–17.0 mm long, 0.5 mm wide, narrowly linear, subacute; lateral lobes 19.0–20.0 mm long, 0.3 mm wide, filiform, acute. Spur 20.0–23.0 mm long, cylindrical, slightly swollen in the apical half. Gynostemium 2.5–3.0 mm long, stigmaphores slightly longer than anther channels, auricles prominent. Figs. 39, 45–46.

*Habitat:* Terrestrial in leaf-litter in coastal forest, remnant lowland forest and in *Pinus* plantation in lower montane forest. Alt. 50–760 m.

*Distribution*: New Guinea.

*Representative specimen*: Papua New Guinea. Upper Schumann River. Alt. 500 m. Feb 1902. *R. Schlechter 14175* (B†); Kaiser-Wilhelms-Land. Im Humus der Wälder des Torricellii-gebirges. Alt. ca. 400 m. Mar 1902. *R. Schlechter 15707* (B†); Zuid Nieuw Guinea. *G.M. Versteeg 1678* (L!), Milne Bay Prov., Radeki. Alt. 500 m. 11 Apr 1961. *N.E.G. Cruttwell 1161* (K!, RENZ!); Madang Province. Basamuk Bay SE of Madang. Alt. below 50 m. 16 Mar 2007. *W.N. Takeuchi & D. Ama 21767* (AMES!, LAE!); Morobe Prov. Lae, hills above 9-10 mile. Alt. 250 m. Oct 2003. *W.N. Takeuchi & D. Ama 16655* (LAE!); Bulolo Plantation. Alt. 760 m. 11 Feb 1980. *A. Kairo 272* (K!, L!, LAE!); Bougainville Distr. Pavairi. Alt. 2800 ft. 16 Jan 1967. *C. Ridsdale & P. Lavarack NGF 30584* (K!, LAE!, RENZ!). Fig. 44.

*Notes: Habenaria dryadum* can be distinguished from *H. dracaenifolia* by having acuminate leaves with bristle-like mucro (*vs* leaves acuminate), shorter anterior petal lobes (15–18 mm *vs* 22–23 mm), shorter lip lateral lobes (19–20 mm *vs* 22–23 mm) and longer spur (20–23 mm *vs* 16–18 mm). The taxonomic status of both species requires further study.

*H. dryadum* var. *major* differs from the typical variety in the more robust habit, larger flowers and the more incurved spur (Schlechter, 1911).

3.3. ***Habenaria micholitziana*** Kraenzl. & Schltr. *in* Kraenzl., Orch. Gen. & Sp. 1: 233. 1901. TYPE: New Guinea. *W. Micholitz s.n*. (B†).

Plant up to 95.0 cm tall, with 3–4 sheaths below the middle, leafy near the middle. Leaves 7–8, approximate, ovate-oblong to oblong-lanceolate, apex acuminate. Inflorescence racemose, scales on peduncle numerous, acuminate; rachis 20.0 cm long, densely many-flowered. Floral bracts as long as the ovary lanceolate, acuminate. Pedicel with ovary 23.0–25.0 mm long. Dorsal sepal 15.0 mm long, concave, ovate, acuminate. Lateral sepals 15.0 mm long, deflexed, obliquely ovate, apex acuminate. Petals 15.0–18.0 mm long in total, bilobed; anterior lobe longer than the posterior lobe; posterior lobe linear, slightly longer than dorsal sepal. Lip 3-lobed at the base; middle lobe 20.0 mm long, filiform, longer than lateral lobes; lateral lobes filiform. Spur 25.0 mm long, slightly sigmoid, in basal 2/3 filiform, in the apical 1/3 abruptly inflated, with a dorsal groove when dried. Gynostemium with stigmaphores and anther channels equally long.

*Habitat:* No data.

*Distribution*: New Guinea.

*Representative specimen*: Papua New Guinea. SW part of country. Feb 1895. *W. Micholitz s.n*. (B†).

*Notes:* This is a poorly known species known from the type only. Unfortunately, most probably the only collection was destroyed in the fire of herbarium in Berlin. The above presented characteristic is based on the original description of the species and some data (*e.g*. leaves size, gynostemium length) are missing. *Habenaria micholitziana* resembles *H. dracaenifolia*, but has somewhat larger flowers (sepals 15 mm *vs* 11–12 mm long), with longer spur with a dorsal groove seen on dried flowers.

3.4. ***Habenaria devogeliana*** Kolan., Szlach., Kras & S. Nowak, ***sp. nov*.** TYPE: Papua New Guinea. *G.D. Weiblen 807* (Holotype: AMES!).

*Species similar to H. micholitziana Kraenzl. & Schltr. in Kraenzl., distinguished by twice smaller lip (middle lobe 10 *vs* 20 mm long), spur shorter than pedicellate ovary (*vs* equal or slightly longer), stigmaphores longer than anther channels (*vs* equally long). The lip middle lobe which is longer than lateral lobes distinguishes the new species from H. dracaenifolia Schltr. and H. novae-hiberniae Schltr*.

**Figure 47:**
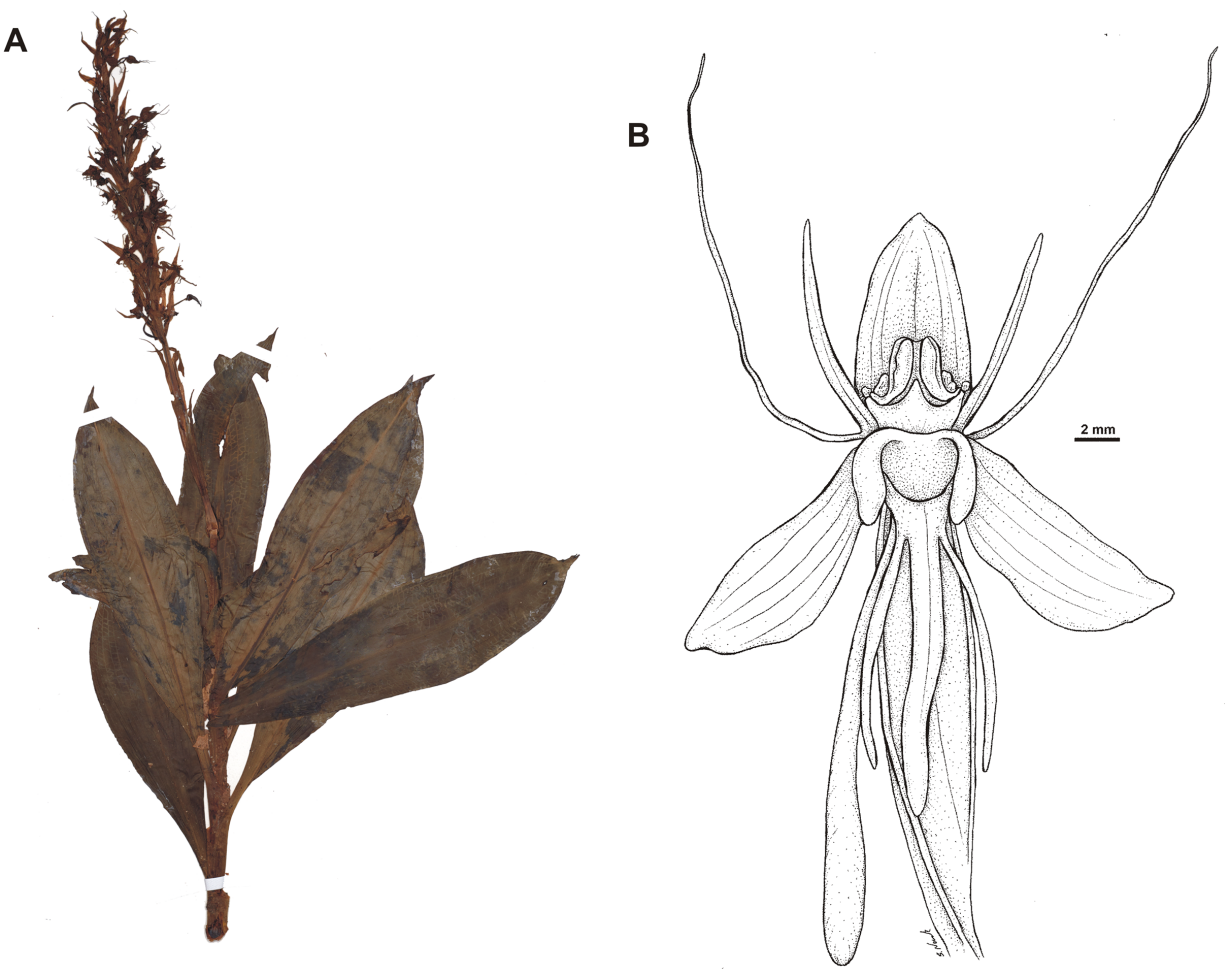
*Habenaria devogeliana* Kolan., Szlach., Kras & S.Nowak. (A) habit; (B) flower (drawn from *Weiblen 807*; AMES).

Plant ca 35.0 cm tall, lower part of the stem not observed. Leaves 8, approximate, shortly petiolate; petiole 3.0 cm long; blade up to 17.0 cm long and 5.0 cm wide, elliptic-obovate, shortly acuminate. Inflorescence racemose, scales on peduncle numerous, acuminate; rachis 17.0 cm long, densely many-flowered. Floral bracts 15.0 mm long, lanceolate, acuminate. Pedicel with ovary 17.0 mm long. Dorsal sepal 8.0 mm long, 4.0 mm wide, concave, ovate, obtuse. Lateral sepals 8.0 mm long, 2.5–3.0 mm wide, deflexed, obliquely obovate, apex obtuse. Petals bilobed; anterior lobe ca 20 mm long, filiform, longer than the posterior lobe; posterior lobe 10.0 mm long, linear. Lip 3-lobed above short claw; middle lobe 10.0 mm long, 1.0 mm wide, linear, obtuse; lateral lobes 8.5 mm wide, filiform, obtuse. Spur 14.0 mm long, cylindrical, inflated in the apical 1/3. Gynostemium 3.5–4.0 mm long; stigmaphores longer than anther channels, ligulate, pendent, basally surrounding spur orifice; connective narrow, apically emarginated, anther chambers basally parallel, apically attenuate, upcurved and spreading; rostellum middle lobe triangular, acute, reaching half the chambers’ length, lateral lobes relatively short, upcurved, viscidia small, elliptic; auricles large, subglobose. Fig. 47.

*Etymology*: Dedicated to Eduard de Vogel, an eminent botanist working on orchids of New Guinea.

*Habitat:* Primary hill forest with 40–50 m canopy. Alt. 600 m.

*Distribution*: New Guinea (Papua New Guinea).

*Representative specimen*: Papua New Guinea. Prov. Eastern Highlands. Near “Wara Oh” camp. Crater Mountains, Wildlife Management Area. Alt. 600 m. 6 Jul 1996. *G.D. Weiblen 807* (AMES!). Fig. 44.

*Notes:* The new species is easily distinguished from *Habenaria novae-hiberniae* and *H. dracaenifolia* based on the lip middle lobe which is longer than lateral lobes in *H. devogeliana* Unlike *H. micholitziana* the lip is small (middle lobe 10 *vs* 20 mm long), the spur is shorter than pedicellate ovary (*vs* equal or slightly longer), and the stigmaphores are longer than anther channels (*vs* equally long).

3.5. ***Habenaria novae-hiberniae*** Schltr., Fl. Schutzgeb. Südsee: 79. 1905. TYPE: Papua New Guinea. *R. Schlechter 14698* (B†, Lectotype, *designated here*: AMES!).

**Figure 48:**
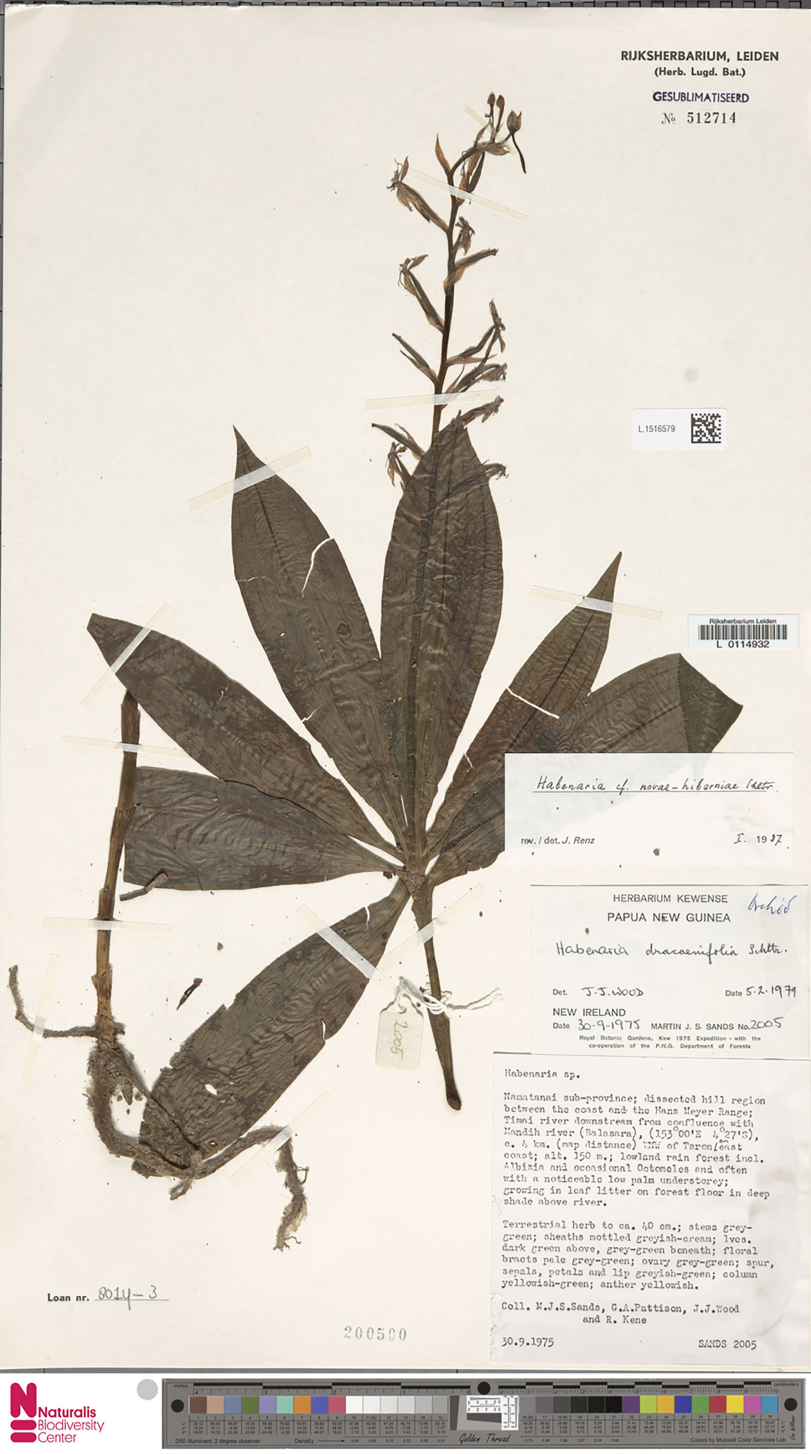
*Habenaria novae-hiberniae* Schltr., herbarium specimen at Naturalis Biodiversity Center, L.1516579. (CC0 1.0; https://data.biodiversitydata.nl/naturalis/specimen/L.1516579).

**Figure 49:**
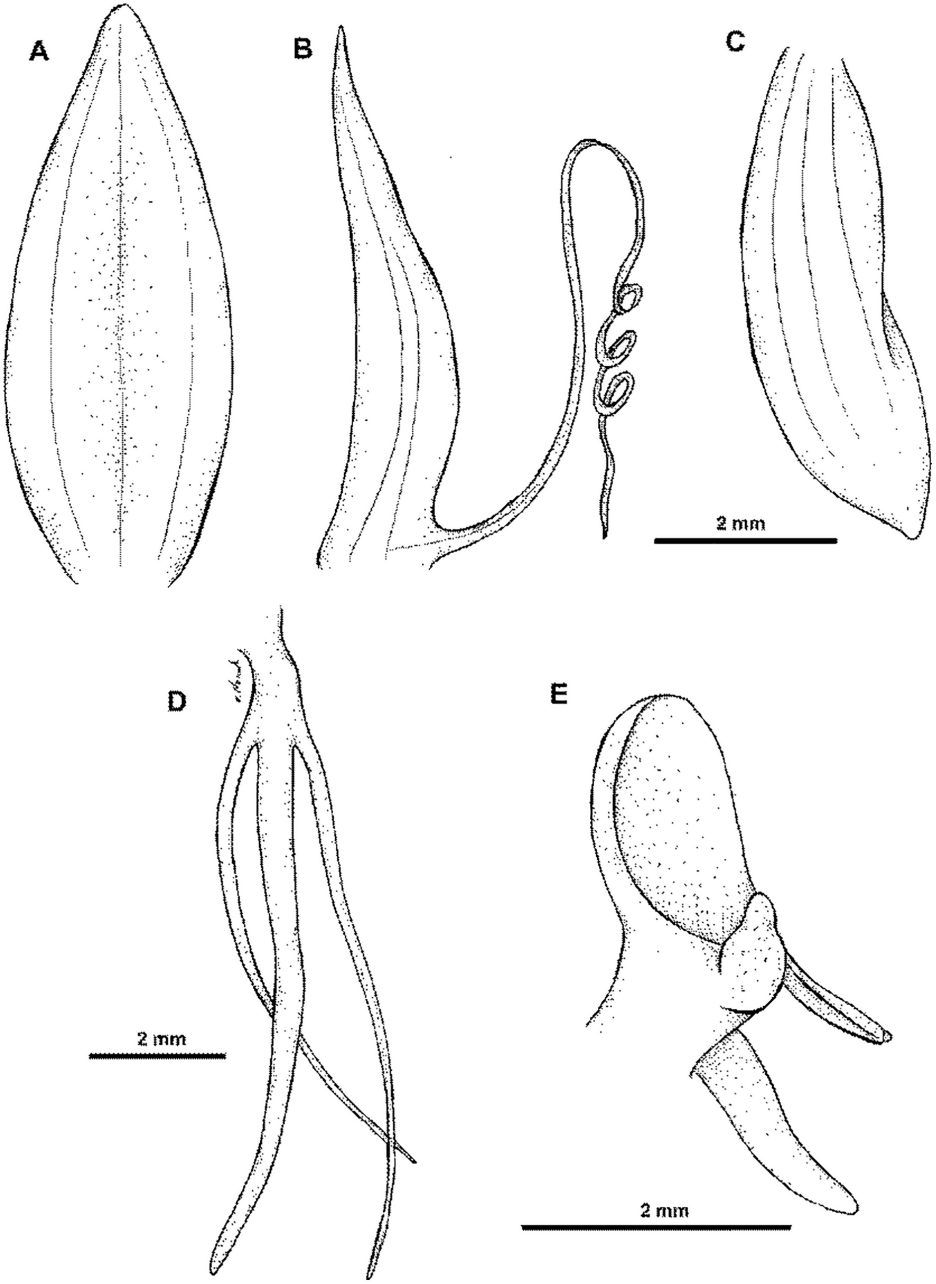
*Habenaria novae-hiberniae* Schltr.: (A) dorsal sepal; (B) petal; (C) lateral sepal; (D) lip; (E) gynostemium (drawn from *Schlechter 14698*; AMES).

Plant up to 75.0 cm tall, with several sheaths in the lower half, leafy in the middle part. Leaves about 6, arranged in a rosette, erect-patent to patent, 10.0–15.0 cm long, 2.0–2.5 cm wide, narrowly elliptic, acute to acuminate. Inflorescence up to 22.0 cm long, laxly 10–20-flowered. Floral bracts ca 16.0 mm long, lanceolate, acuminate. Pedicel with ovary ca 30.0 mm long, narrowly cylindrical. Flowers greenish yellow. Dorsal sepal 6.0–9.0 mm long, 5.0 mm wide, strongly concave, elliptic-obovate, obtuse or acute, 3-veined. Lateral sepals 6.0–10.0 mm long, 4.5 mm wide, obliquely ovate to ovate-lanceolate, obtuse to subacute, 3-veined. Petals bilobed just above the base; anterior lobe filiform, almost twice as long as the posterior lobe, 1-veined; posterior lobe ca 5.0–10.0 mm long, linear, subobtuse to subacute, 1-veined. Lip 3-lobed above base; middle lobe 6.0–7.0 mm long, linear, attenuate towards subobtuse or subacute apex; lateral lobes about 12.0 mm long, filiform, long-acuminate. Spur 9.0–18.0 mm long, narrowly cylindrical, somewhat swollen in the apical half, subacute to subobtuse. Gynostemium ca. 3.0 mm long; stigmaphores as long as the anther channels; auricles prominently stalked, large, prominent. Figs. 42, 48–49.

*Habitat:* Terrestrial in leaf-litter and on muddy stream banks, in rainforest and in lower montane forest. Alt. 70–1,200 m.

*Distribution*: New Guinea, Solomon Islands, Vanatu.

**Figure 50:**
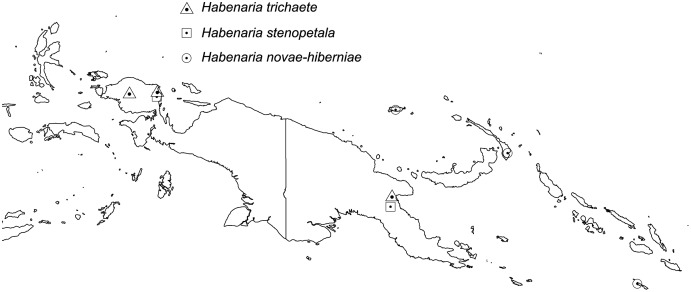
Distribution of *Habenaria novae-hiberniae*, *H*. *stenopetala* and *H*. *trichaete*.. Base map downloaded from www.naturalearthdata.com.

*Notes: Habenaria novae-hiberniae* is similar to *H. trichaete*. Unlike the latter, however, it has narrow leaves (up to 2.5 cm), pedicellate ovary ca twice longer than floral bracts and lip lateral lobes ca twice longer than the middle one.

*Representative specimen*: Papua New Guinea. New Ireland, near Punam. Alt. 600 m. Jul 1902. *R. Schlechter 14698* (AMES!); New Ireland, Namatanai subprovince, Timai river downstream from confluence with Mandish River (Balasara), 153°00′E 4°27′S, c. 4 km WNW of Taron on east coast. Alt. 150 m. 30 Sep 1975. *M.J. Sands, G.A. Pattison, J.J. Wood & R. Kene 2005* (AMES!, K!, L!, RENZ!); South Kangua village, Rennell. Alt. 210 ft. 5 May 1969. *I. Gaful & al. 14754* (LAE!); Manus Island, in Mundrau limestone depression, south or Kari village. ca. 6.5 km inland from N coast in eastern Manus. Alt. 125 m. *M. J. Sands & al. 2618* (K!, L!); Alt. 70 m. Apr 1989. *L. J. Brass 13760* (AMES!, BO!). Fig. 50.

Type specimen at Harvard University Herbaria has no collection number on the label, however all data about gathering and morphology of the specimen are consistent with the protologue, therefore we have decided to designate its as a lectotype.

3.6. ***Habenaria stenopetala*** Lindl., Gen. Sp. Orchid. Pl.: 319. 1835. TYPE: India. *J.F. Royle s.n*. (K).

= *Habenaria delessertiana* Kraenzl., Annuaire Conserv. Jard. Bot. Genève 1: 108. 1897. TYPE: Philippines. *H. Cuming 2086* (HBG).

= *Habenaria linearipetala* Hayata, Icon. Pl. Formosan. 4: 126. 1914. TYPE: China. *Sasaki s.n*. (TI, T - photo).

= *Habenaria sutepensis* Rolfe *ex* Downie, Bull. Misc. Inform. Kew 1925: 420. 1925. TYPE: Thailand. *Kerr 277* (K).

= *Habenaria amanoana* Ohwi, J. Jap. Bot. 31: 136. 1956. TYPE: Japan. *T. Amano 7535* (TNS).

**Figure 51:**
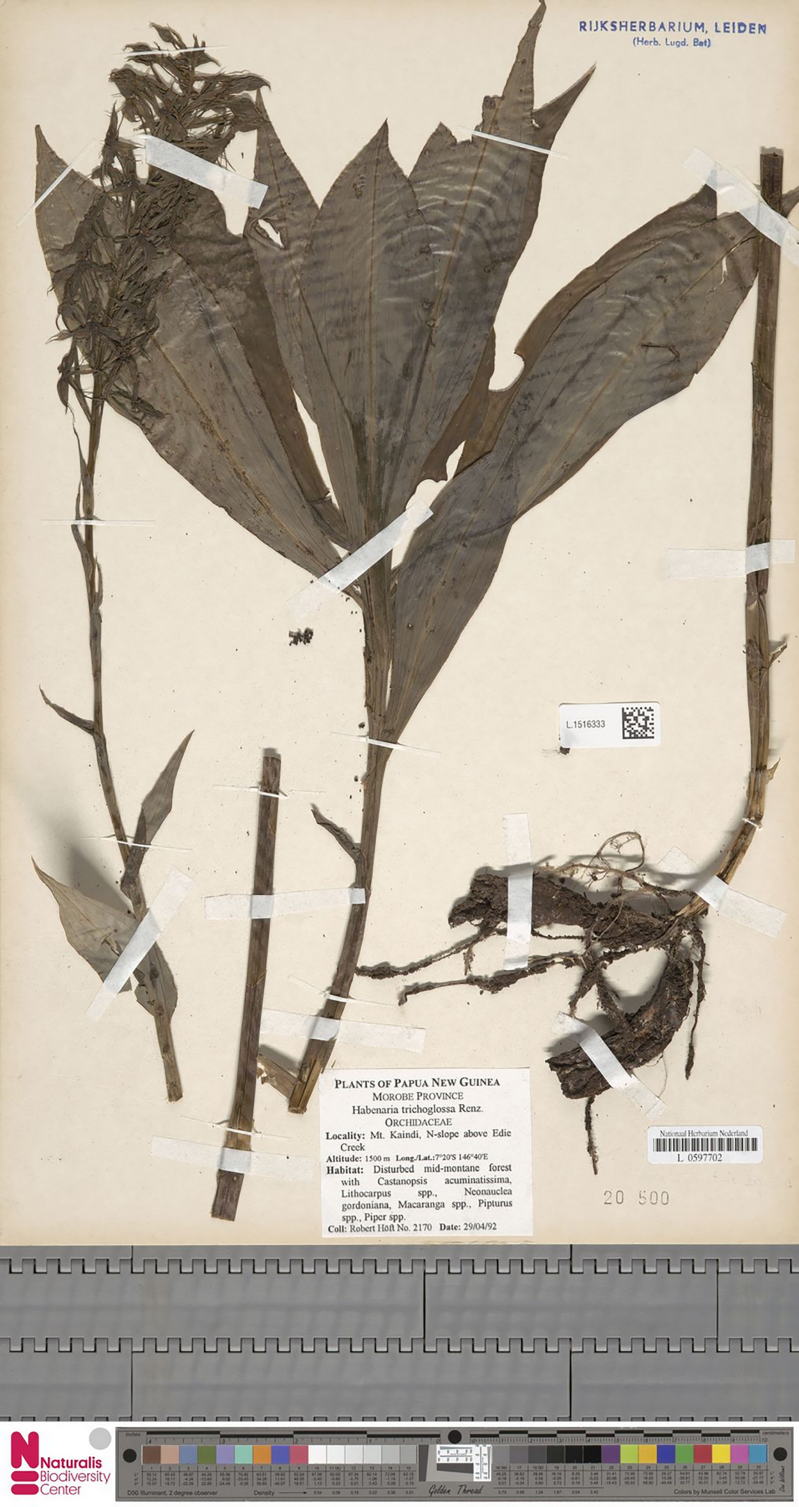
*Habenaria stenopetala* Lindl., herbarium specimen at Naturalis Biodiversity Center, L.1516333. (CC0 1.0; https://data.biodiversitydata.nl/naturalis/specimen/L.1516333).

**Figure 52:**
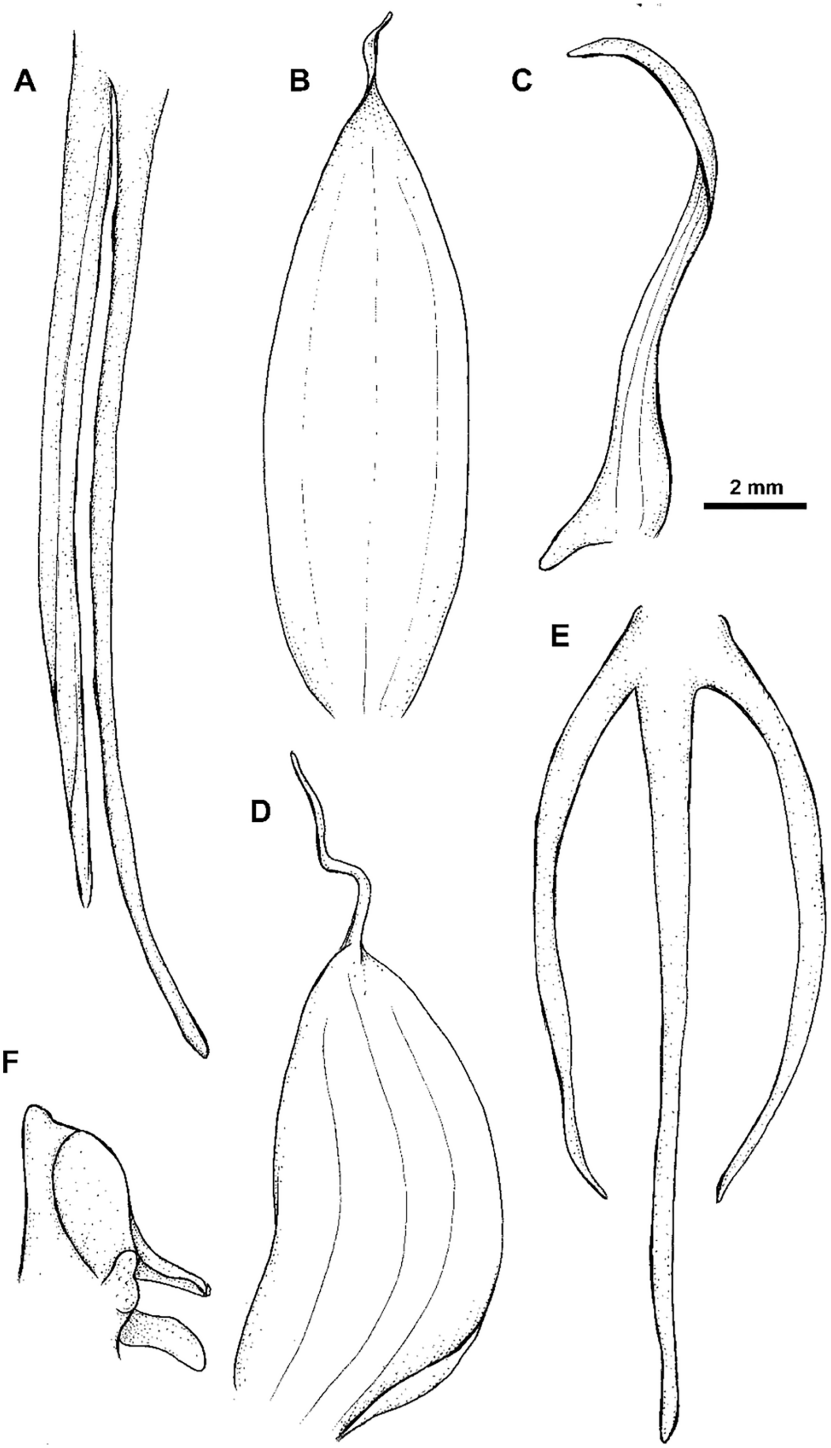
*Habenaria stenopetala* Lindl.: (A) ovary and spur; (B) dorsal sepal; (C) petal; (D) lateral sepal; (E) lip; (F) gynostemium (drawn from *Höft 2170*; L).

Plant up to 90.0 cm tall, leafy near the middle, with numerous bract-like leaves above. Leaves 5–8, 8.0–16.0 cm long, 3.0–5.5 cm wide, basally contracted into amplexicaul sheath, blade elliptic to oblong or oblong-lanceolate, acute or acuminate. Inflorescence 10.0–20.0 cm long, densely many-flowered. Floral bracts 15.0–28.0 mm long, lanceolate to ovate-lanceolate, often longer than flowers, apex aristate. Pedicel with ovary 15.0–22.0 mm long, cylindrical-fusiform, glabrous. Flowers green or greenish white. Dorsal sepal 14.0–16.0 mm long, 4.0–5.0 mm wide, concave, erect, ovate-elliptic, long acuminate or caudate-aristate at the apex, 3-veined. Lateral sepals 16.0–18.0 mm long, 5.0–6.0 mm wide, reflexed, obliquely ovate, long acuminate or caudate-aristate at the apex, 3-veined. Petals bilobed; anterior lobe small, ca 2.5 mm, narrowly falcate; posterior lobe 10.0–13.0 mm long, 1.0–1.8 mm wide, linear, acuminate, 1-veined. Lip 3-lobed, 10.0–15.0 mm long in total; middle lobe up to 17.0 mm long, linear or ligulate, subacute; lateral lobes up to 14.0 mm long, linear or subulate, acute. Spur 15.0–26.0 mm long, pendulous, cylindrical, obtuse. Gynostemium about 5.3 mm long; stigmaphores about as long as anther channels, oblong-clavate. Figs. 51–52.

*Habitat:* Terrestrial in shade of riverine montane forest. Alt. 1,800 m.

*Distribution*: India, Nepal, Bhutan, Myanmar, China, Thailand, Vietnam, Taiwan, Philippines, New Guinea (Indonesia).

*Representative specimen*: Indonesia. Prov. West Papua. Ransiki–Anggi Lakes road. Alt. 1300 m. 29 Jul 2014. *A. Schuiteman & al. 2014-68* (BO, K—Schuiteman & Wanma, 2017). Papua New Guinea. Mt. Kaindi, N-slope above Edie Creek. 29 Apr 1992. *R. Höft 2170* (L!). Fig. 50.

*Notes:* Usually *Habenaria stenopetala* is distinguished from other species of this group by having very short anterior petals lobe reaching ca 2.5 mm and having sepals with caudate-aristate apices. However, in our opinion the only constant character which can be used to separate *H. stenopetala* and *H. trichaete* is the length of stigmaphores which distinctly longer than the anther channels in *H. trichaete* and about as long as anther channels in *H. stenopetala*.

3.7. ***Habenaria trichaete*** Schltr., Repert. Sp. Nov. Regni Veg. Beih. 1: 14. 1911. TYPE: Papua New Guina (Deutsch Neu-Guinea). *R. Schlechter 16383* (B†); *A. Millar 22945* (Neotype, *designated here*: LAE!, Isoneotypes: L!, RENZ!).

**Figure 53:**
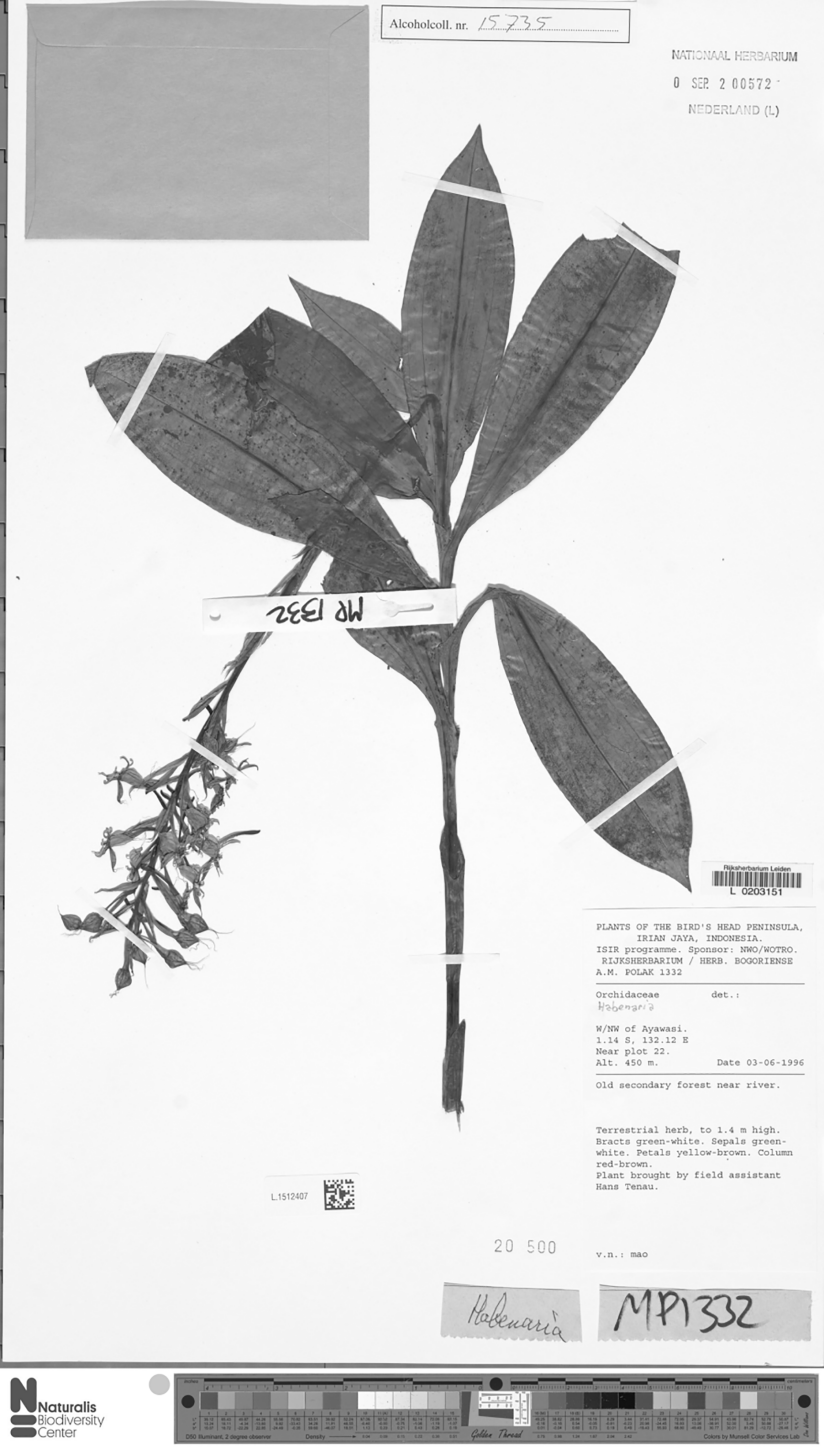
*Habenaria trichaete* Schltr., herbarium specimen at Naturalis Biodiversity Center, L.1512407. (CC0 1.0; https://data.biodiversitydata.nl/naturalis/specimen/L.1512407).

**Figure 54:**
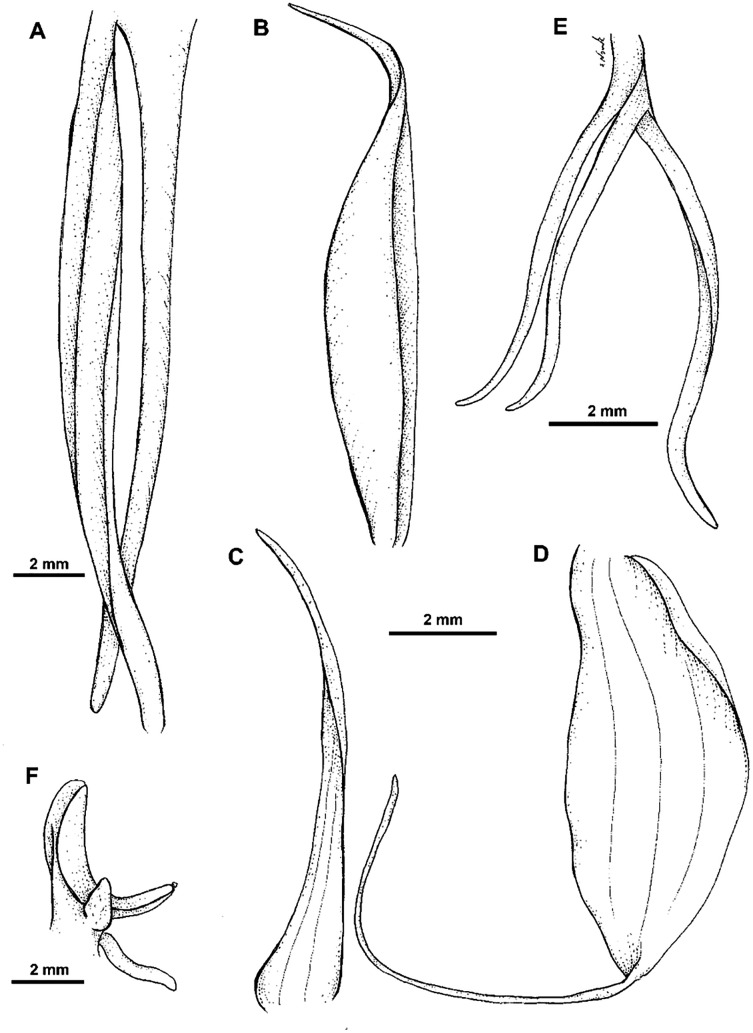
*Habenaria trichaete* Schltr.: (A) ovary and spur; (B) dorsal sepal; (C) petal; (D) lateral sepal; (E) lip; (F) gynostemium (drawn from *Millar 22945*; L).

Plant up to 150.0 cm tall, in basal half with 5–7 scale-like leaves, 7–8-leaved near the middle. Leaves erect-patent, approximate, up to about 18.0 cm long, 4.5–5.0 cm wide, elliptic, elliptic-lanceolate, acuminate. Inflorescence racemose, peduncle-scales bract-like; rachis 13.0 cm long, densely 15–25-flowered. Floral bracts as long as or slightly longer than the ovary, lanceolate to elliptic-lanceolate, acuminate. Pedicel with ovary 17.0–20.0 mm long, cylindrical-subclavate. Flowers greenish. Dorsal sepal 8.0–10.0 mm long, 4.0–5.0 mm wide, oblong-ovate to elliptic-ovate, apex with a bristle-like prolongation 4.0–6.0 mm long, 3- or 5-veined. Lateral sepals about 7.5–10.0 mm long, obliquely oblong ovate, apex with a long bristle-like prolongation 4.0–6.0 mm long, 4-veined. Petals bilobed; anterior lobe 13.0–20.0 mm long, filiform with a bristle-like apex, acuminate, 1–2-veined; posterior lobe 1.0–2.0 mm long, dentate, obtuse, 1-veined. Lip 3-lobed above short claw; middle lobe 8.0–14.0 mm long, 1.0 mm wide, linear, narrowly obtuse; lateral lobes 7.0–12.0 mm long, 0.4–0.6 mm wide, linear-filiform, somewhat falcate, narrowly obtuse. Spur 14.0-21.0 mm long, cylindrical, slightly swollen towards the apex, narrowly obtuse. Gynostemium ca. 5.5–6.0 mm long; stigmaphores distinctly longer than the anther channels; auricles prominent. Figs. 39, 53–54.

*Habitat:* Terrestrial in leaf-litter in hill and lower montane forest (Schuiteman, Vermeulen & De Vogel, 2001–2010) at the altitude of 400–1,350 m.

*Distribution*: New Guinea.

*Representative specimen*: Indonesia. Irian Jaya, the Bird’s Head Peninsula, W/NW of Ayawasi, 1.14 S, 132.12 E. Alt. 450 m. 3 Jun 1996. *A.M. Polak 1332* (L!); North East Kepala Burung, Irian Jaya. Kecamatan Manokwari. Mupi Dessa. Arfak Mts., Mupi Valley system. On trail from Mupi to Humeibou, near base camp. 18 Apr 1995. *U.W. Mayar & al. 445* (K!). Papua New Guinea. Kaiser-Wilhelm Land: im humus der Urwälder am Djamu, im Gebiete vun Umbili. Alt. 400 m. Aug. 1907. *R. Schlechter 16383* (B†); Bupu village, Wampit. Alt. 1,300 m. 13 Jul 1967. *A. Millar 22945* (L!, LAE!, RENZ!). Fig. 50.

*Notes: Habenaria trichaete* is similar to *H. novae-hiberniae*, as both share similar habit and relatively small flowers with lip lobes not exceeding 12 mm in length. In the former species, however, the leaves are wider (4.5–5 cm *vs* 2–2.5 cm), the floral bract is as long as or longer than pedicellate ovary (*vs* prominently shorter), and the lip middle lobe is longer than both laterals (*vs* lip middle lobe prominently shorter than lateral ones).

At least three species can be confused with *Habenaria trichaete*, and the original material unfortunately does not exist. For this reason we decided to designate the neotype and chose a collection of *Millar 22945* that corresponds well with the original description and the drawing made by the author of the name (Fig. 54).

3.8. ***Habenaria trichoglossa*** Renz, Pl. Syst. Evol. 155 (14): 322. 1987. TYPE: Papua New Guinea. *J. Renz 12344* (Holotype: RENZ!).

**Figure 55:**
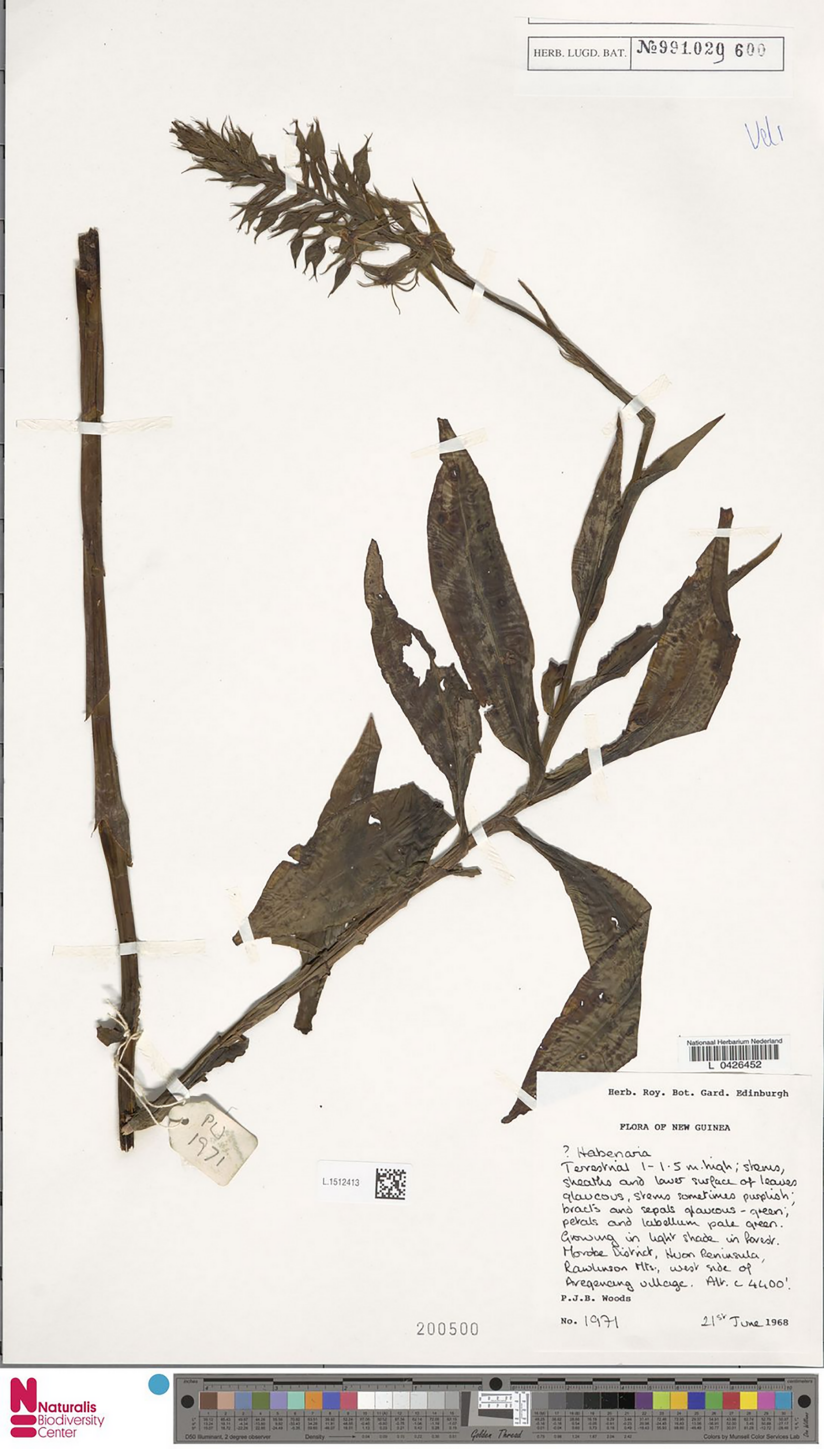
*Habenaria trichoglossa* Renz, herbarium specimen at Naturalis Biodiversity Center, L.1512413. (CC0 1.0; https://data.biodiversitydata.nl/naturalis/specimen/L.1512413).

**Figure 56:**
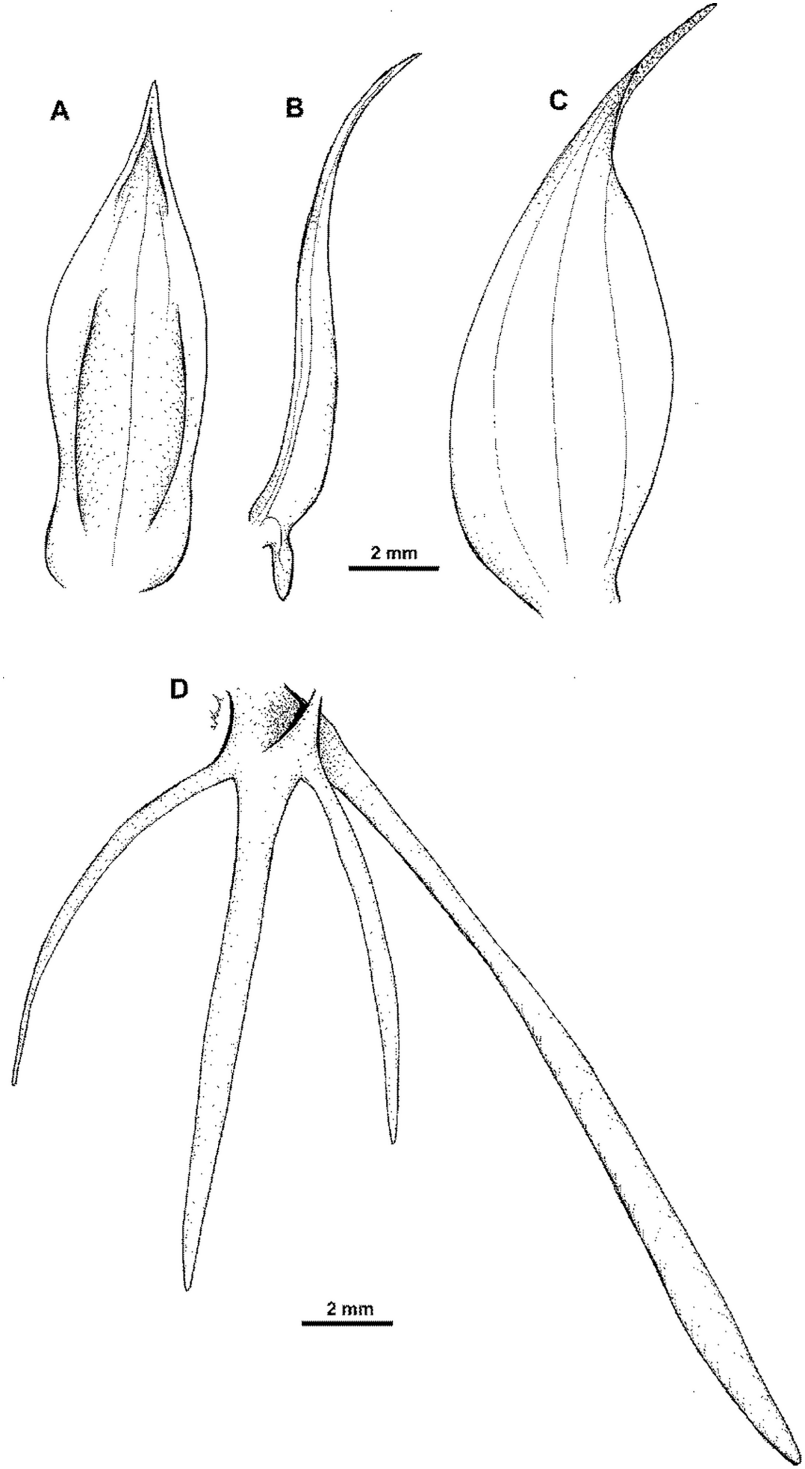
*Habenaria trichoglossa* Renz: (A) dorsal sepal; (B) petal; (C) lateral sepal; (D) lip (drawn from *Woods & al. 1971*; L).

**Figure 57:**
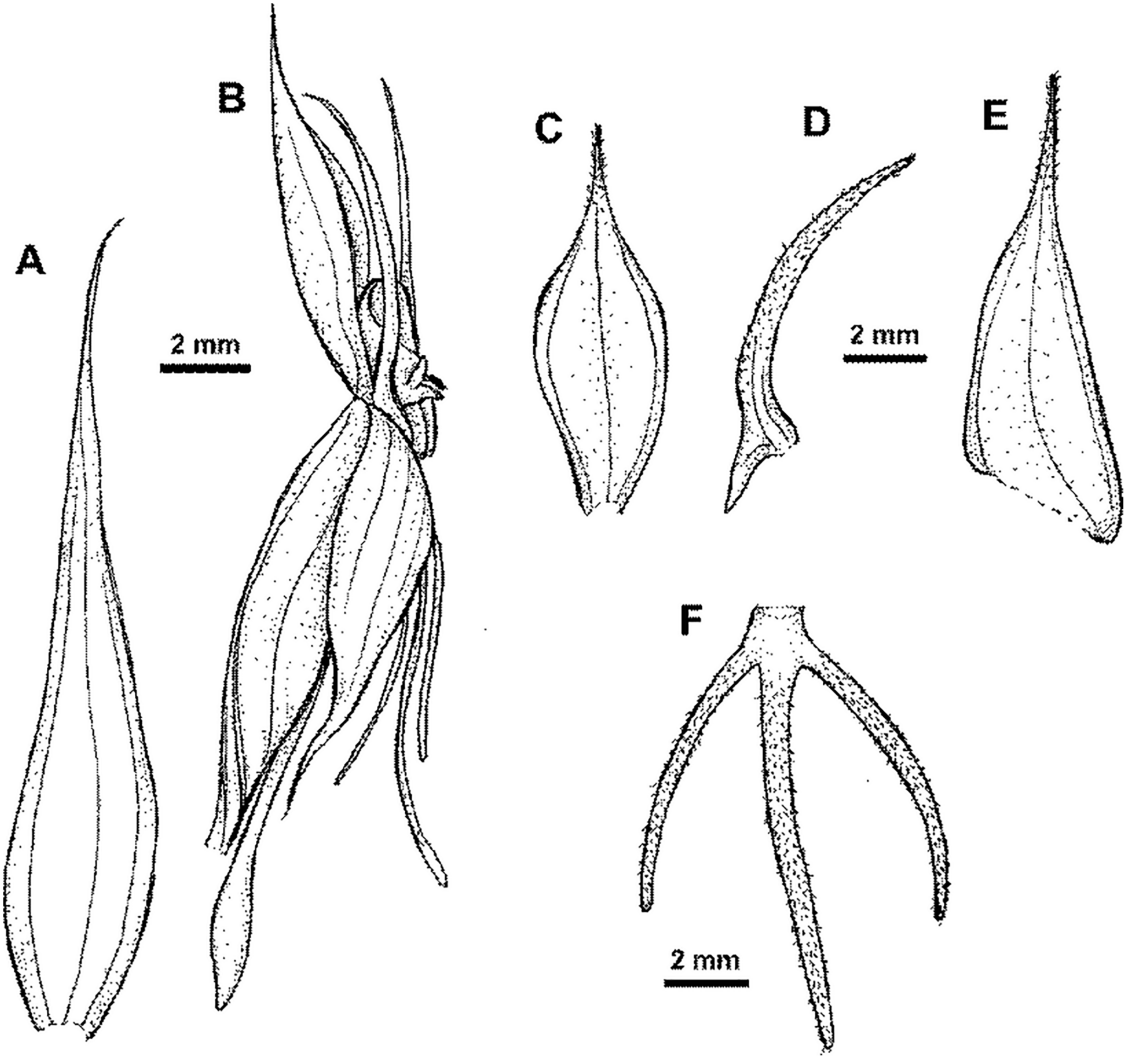
*Habenaria trichoglossa* Renz: (A) bract; (B) flower; (C) dorsal sepal; (D) petal; (E) lateral sepal; (F) lip (drawing of *Renz 12344*; RENZ).

Plant up to 120.0 cm tall, in upper half 6–10-leaved. Leaves 15.0–30.0 cm long, 3.0–5.0 cm wide, obovate (lower ones) to oblong-lanceolate (upper ones), apex acuminate, base narrowed. Inflorescence racemose, peduncle-scales bract-like; rachis 12.0–15.0 cm long, densely many-flowered. Floral bracts 22.0 mm long, lanceolate, slightly longer than the ovary, apex acuminate. Pedicel with ovary 18.0–23.0 mm long, shortly pedicellate, glabrous. Flowers greenish. Dorsal sepal 13.0–15.0 mm long, 3.5–6.0 mm wide, ovate-lanceolate to oblong ovate, apex long apiculate, apiculum 3–4 mm long, papillose-tomentose, 5-veined. Lateral sepals 13.0–15.0 mm long, 4.5–6.0 mm wide, patent to more or less deflexed, oblong ovate, long apiculate, papillose-tomentose, 5-veined. Petals 9.0–12.0 mm long, 1.5–2.0 mm wide, falcate, linear-lanceolate, papillose-tomentose especially towards the apex, with a short tooth or only dilated at the base, apex acuminate, 1-veined. Lip 3-lobed ca1 mm above the base, papillose-tomentose; middle lobe 10.0–13.0 mm long, linear, rather thick, acuminate; lateral lobes 9.0–10.0 mm long, narrowly linear-filiform, acuminate. Spur 22.0–25.0 mm long, narrowly cylindrical, about as long as the ovary or a little longer, dilated at the mouth only, apex rather obtuse. Gynostemium 4.0–5.0 mm long; stigmaphores 2.0–3.0 mm long, anther channels 2.0–2.5 mm long. Figs. 55–57.

*Habitat:* Humid rainforest and lower montane forest. Alt. 750–1,100 m.

*Distribution*: New Guinea.

**Figure 58:**
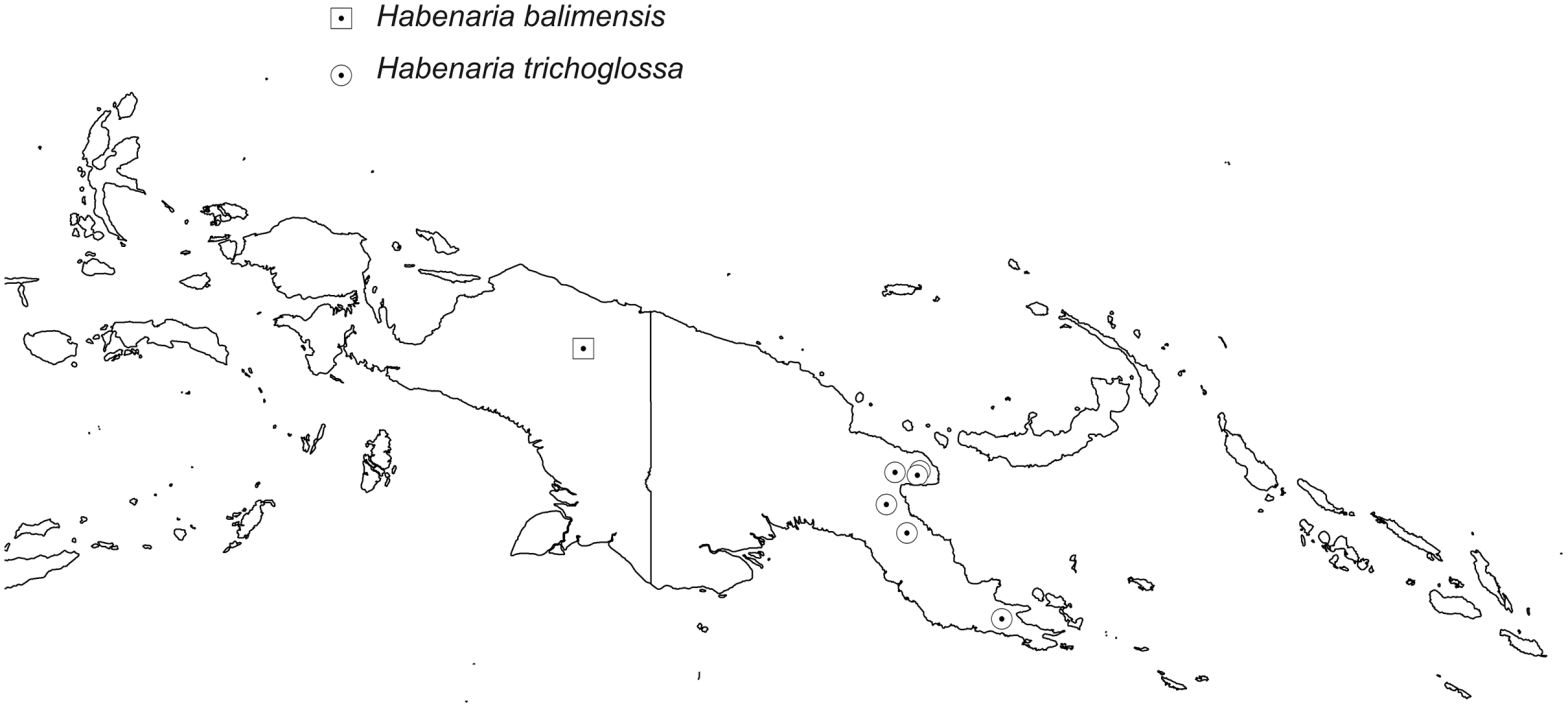
Distribution of *Habenaria trichoglossa* and *H. balimensis*. Base map downloaded from www.naturalearthdata.com.

*Notes: Habenaria trichoglossa* resembles *H. stenopetala* and *H. trichaete*. From both *H. trichoglossa* differs by having papillose-tomentose petals and lip. Additionally, unlike in *H. trichaete* the apices of lateral sepals of *H. trichoglossa* are not extended into long, stiff setae.

*Representative specimens*: Papua New Guinea. Morobe. Distr. Boana—road (Lae to Boana). In silva pluvialis ad primum saltum. Alt. 1,000 m. 3 Aug. 1979. *J. Renz 12344* (RENZ!); Mountain slope above village Sopa, rain-forest. Alt. 1,100 m. 19 Jun 1962. *T.G. Hartley TGH 10362* (LAE!, RENZ!); Sawmill Creek, W of Bulolo. Alt. 750 m. 23 Feb 1970. *H. Streimann & A. Kairo, NGF 47510* (LAE, RENZ!); Milne Bay Distr., Rabaraba Subdistr., Agaun. 28 May 1954. *N.E.G. Cruttwell 441* (K!, RENZ!); Aug 1973. *N.E.G. Cruttwell 1641* (LAE!), Morobe District. Tobou. June 1937. *M.S. Clemens 6526* (AMES!); Morobe District, Huon Peninsula, Rawlinson Mts., west side of Aregenang village. Alt 4400 ft. 21 Jun 1968. *P.J. Woods & al. 1971* (AMES!, BO!, E!, K!, L!, LAE!). Fig. 58

4. ***Habenaria balimensis*-group**

Plants with leaves distributed along stem. Petals divided almost to the base, both lobes entire. Lip lobes similar, narrow.

4.1. ***Habenaria balimensis*** Ormerod, Orchadian 16(11): 517 (-518; fig.). 2011. TYPE: Indonesia. *L.J. Brass 11746* (Holotype: AMES!).

**Figure 59:**
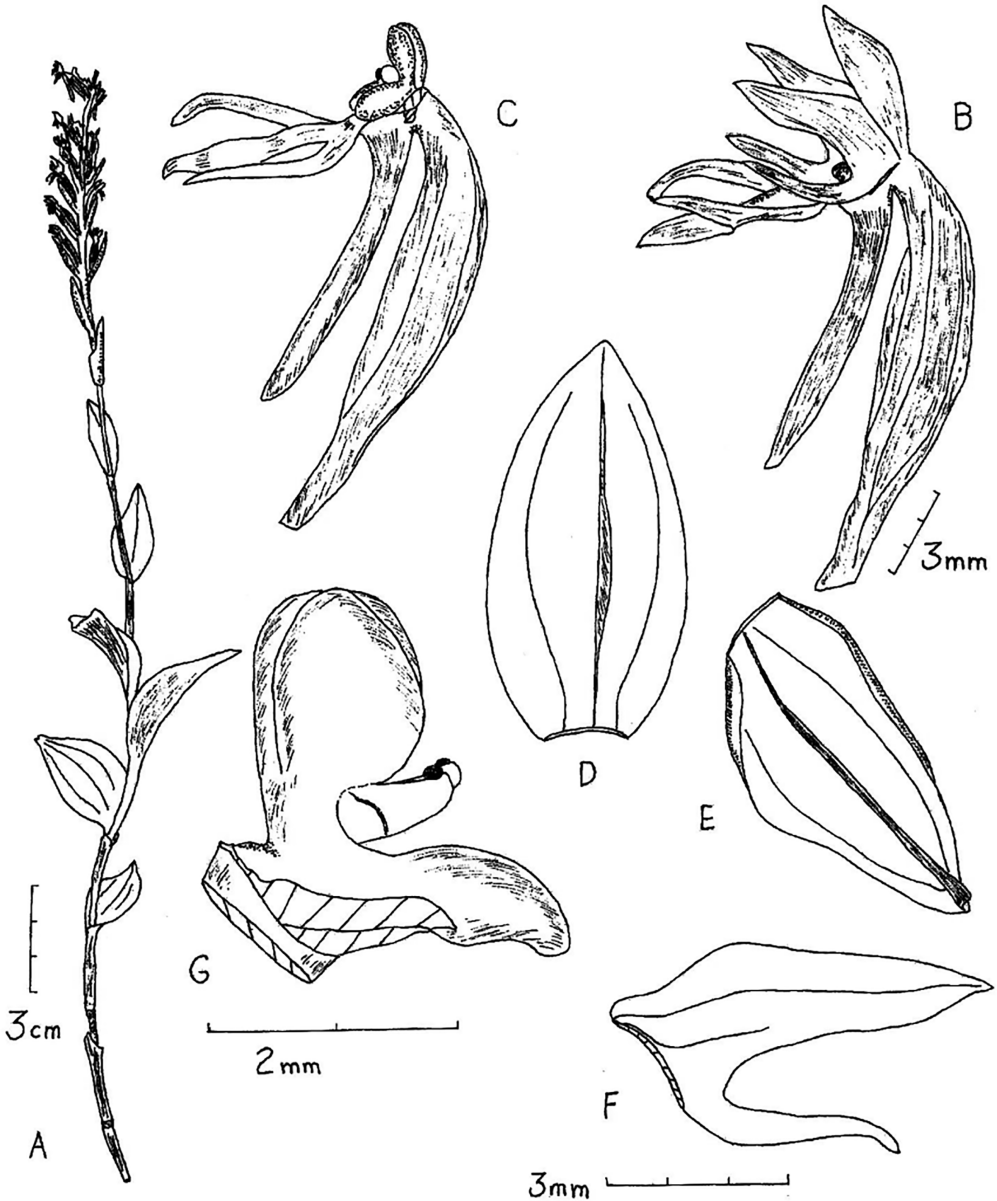
*Habenaria balimensis* Ormerod: (A) plant; (B) flower; (C) flower without tepals; (D) dorsal sepal; (E) petal; (F) lateral sepal; (G) gynostemium (Ormerod’s original drawing of *Brass 11746*; AMES).

**Figure 60:**
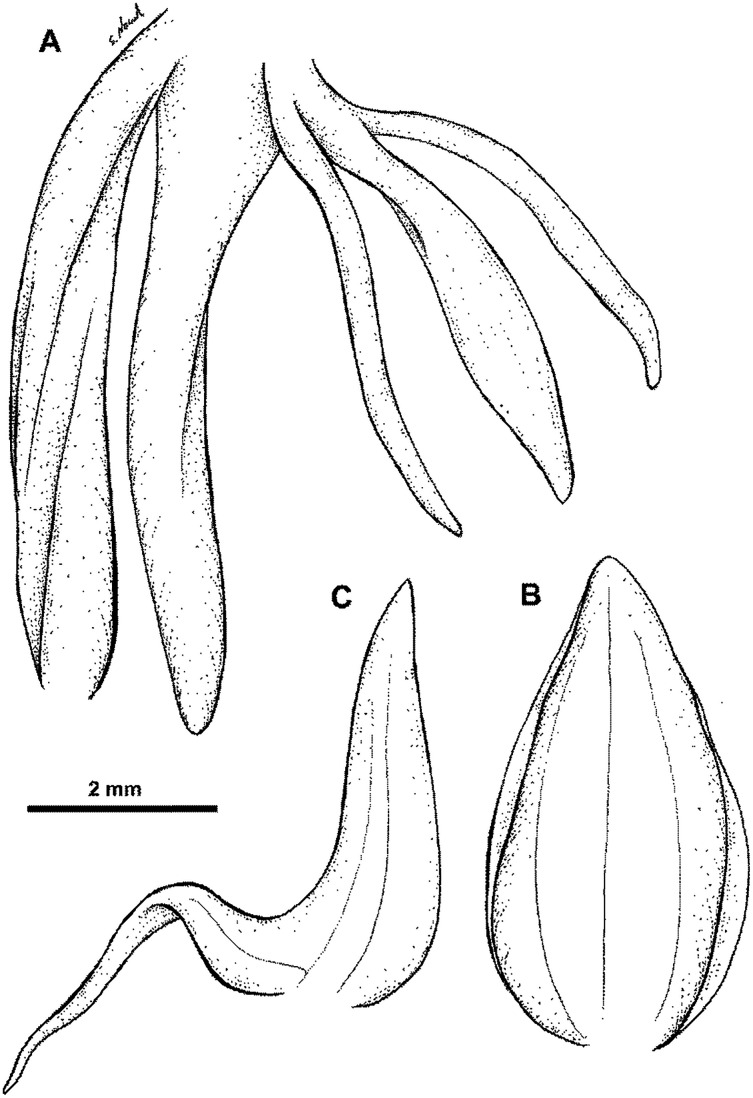
*Habenaria balimensis* Ormerod: (A) ovary with lip and spur; (B) dorsal sepal; (C) petal (drawn from *Brass 11746*; AMES).

Plant almost 40.0 cm tall, in lower half enveloped with tubular sheaths, leafy in the upper third or so. Leaves about 5, distant, 2.3–4.1 cm long, 1.3–2.2 cm wide, ovate, apex subobtuse, sheathing at the base. Inflorescence 15.2 cm long, peduncle with 2 sheathing scales, rachis subdensely 12–15-flowered. Floral bracts up to 17.0 mm long, lanceolate, acute. Pedicel with ovary 13.0 mm long, fusiform, ribbed. Flowers green. Dorsal sepal about 5.5 mm long, 3.0–3.5 mm wide, concave, ovate-elliptic, acute, 3-veined. Lateral sepals 5.5 mm long, 2.9 mm wide, obliquely ovate-elliptic, acute, mid-vein carinate outside, 3-veined. Petals 5.0 mm long, 2.9 mm wide, bilobed above the base; anterior lobe ca. 4.0 mm long, 1 mm wide, linear-lanceolate, acute; posterior lobe 5.0 mm long, 1.9 mm wide, obliquely linear-lanceolate, with acute apex, obscurely 2-veined. Lip 3-lobed above the base; middle lobe 5.0 mm long, 1 mm wide, linear-ligulate, acute; lateral lobes 4.7 mm long, linear-ligulate. Spur 9.0 mm long, cylindrical, acute. Gynostemium 1.8–2.0 mm long; stigmaphores more than twice as long as anther channels. Figs. 59–60.

*Habitat:* Terrestrial in grassy edge of forest. Alt. 1,800 m.

*Distribution*: New Guinea.

*Representative specimen*: Indonesia. Prov. Papua. Balim River. Alt. 1,800 m. Dec 1938. *L.J. Brass 11746* (AMES). Fig. 58.

*Notes: Habenaria balimensis* seems to lack close relatives in New Guinea. It may be recognized by its slender *Peristylus-*like habit, small leaves and rather short inflorescence and bifurcate petals (Ormerod, 2011).

***Incertæ sedis***

Material incomplete or too damaged for identification

*C.E. Carr 10141* (AMES 103512!, BM!, L!, LAE 71465!), *J. Croft s.n*. (LAE 71342!), *R. Schodde 3085* (A!, K!, L!, US!; cf. *H. cruciata*)

***Excluded species***

- *Habenaria dolichocaulon* Schltr., Repert. Sp. Nov. Regni Veg. Beih. 1: 6. 1911; Type: Kaiser-Wilhelms Land: in humus der Wälder des Mimiberges (im Wariatale), alt. 700 m. March 1908, *Schlechter 17414* (B†). ≡ ***Peristylus dolichocaulon*** (Schltr.) P. F. Hunt, Kew Bull. 26 (1): 177. 1971.
- *Habenaria klossii* Ridl., Trans. Linn. Soc. 9: 209. 1916; Type: Dutch New Guinea. Camp Vic. Alt. 5500 ft., Camp III. Alt. 2500 ft. *Kloss s.n*. (BM). ≡ ***Platanthera klossii*** (Ridl.) Efimov *in* Ormerod, Checklist of Papuasian Orchids: 388. 2017.
- *Habenaria lauterbachii* Kraenzl. *ex* K. Schum. & Lauterb., *nom. nud*., Fl. Deutsch. Sudsee 239. 1901; Type: New Guinea. Constantinhafen, in Alangfeldern. 13 Dec 1890. *Lauterbach 1310* (B†, WRSL). ≡ ***Peristylus goodyeroides*** (Don.) Lindl., Gen. Sp. Orchid. Pl.: 299. 1835.
- *Habenaria listeroides* Schltr., Repert. Sp. Nov. Regni Veg. Beih. 1: 9. 1911; Type: Kaiser-Wilhelms Land: in humus der Wälder am Govidjoa (Wariagebiet). Alt. 1,200 m, Jun 1909. *Schlechter 19804* (B†). ≡ ***Peristylus listeroides*** (Schltr.) P. F. Hunt, Kew Bull. 26 (1): 177.
- *Habenaria macra* Schltr., Repert. Sp. Nov. Regni Veg. Beih. 1: 9. 1911; Type: Kaiser Whilems Land: An offenen Abhängen des Finisterregebirges, suf moosigen Kalksteinen. Alt. 1,200 m. Sep 1908. *Schlechter 18203* (B†). ≡ ***Peristylus macer*** (Schltr.) P. F. Hunt, Kew Bull. 26 (1): 177.
- *Habenaria nitida* Schltr., Repert. Sp. Nov. Regni Veg. Beih. 1: 10. 1911; Type: Kaiser-Wilhelms Land: An offeneren Abhängen in den Wäldern des Torricelligebirges. Alt. 700 m. Sep 1909. *Schlechter 20291* (B†). ≡ ***Peristylus nitida*** (Schltr.) P. F. Hunt., Kew Bull. 26 (1): 177. 1971.
- *Habenaria pachyneura* Schltr., Fedde. Repert. Beih. 1: 12. 1911; Type: Kaiser-Wilhelms Land: in humus der Wälder des Bismarckgebirges. Alt. 2,300 m. Nov 1908. *Schlechter 18824* (B†). ≡ ***Peristylus pachyneura*** (Schltr.) P. F. Hunt, Kew Bull. 26 (1): 177. 1971.
- *Habenaria papuana* Kraenzl., Bot. Jahrb. Syst. 18: 188. 1893; Type: New Guinea. Near Finschafen. Essimbu. 10 Apr 1889. *Hellwig 585* (B†). ≡ ***Peristylus papuanus*** (Kraenzl.) J. J. Sm., Nova Guinea, Bot. 12, Bot. 3. 1913.
- *Habenaria philopsychra* Ridl., Trans. Linn. Soc. 9: 208. 1916; Type: New Guinea. Camp XIII. Alt. 10500 ft. *Kloss s.n*. (BM). = ***Peristylus ciliolatus*** J.J.Sm., Bull. Jard. Bot. Buitenzorg, sér. 2(13) 53. 1914; Type (Van Royen, 1979): New Guinea. Treub Range. Alt. 2,400 m. Feb 1913. *Pulle 1096* (BO).
- *Habenaria silvicola* Schltr., Repert. Sp. Nov. Regni Veg. Beih. 1: 6. 1911; Type: Kaiser-Wilhelms Land: Im humus der Wälder des Kanigebirges. Alt. 1,000 m, Aug 1908. *Schlechter 18088* (B†), im humus der Wälder am Wakeak. Alt. 500 m, Aug 1908. *Schlechter 18118* (B†). ≡ ***Peristylus silvicola*** (Schltr.) P. F. Hunt. *in* Kew Bull. 26 (1): 177. 1971.
- *Habenaria triaena* Schltr., Repert. Sp. Nov. Regni Veg. Beih. 1: 7. 1911. Type: Kaiser-Wilhelms Land: Im humus der Wälder des Finisteregebirges, alt. 1,100 m., Sept. 1908, *Schlechter 18169* ≡ ***Peristylus trianae*** (Schltr.) P. F. Hunt *in* Kew Bull. 26 (1): 177. 1971; TYPE: New Guinea. Lower mintane forest. Alt. 850 m. *Schlechter 18169* (B†).
- *Habenaria umbonata* Schltr., Fedde Repert Beih. 1: 8. 1911; Type: Kaiser-Wilhelm Land: im humus der Wälder auf dem Dischore (Wariagebiet). Alt. 1,300 m, Jun 1909. *Schlechter 19660* (B†). *≡* ***Peristylus umbonatus*** (Schltr.) P.F. Hunt, Kew Bull. 26(1): 178. 1971.

## Discussion

The genus *Habenaria* is one of the largest in Orchidaceae but the infrageneric classification of this taxon remains unresolved. As indicated by Bateman et al. (2003) the genus is polyphyletic, however, so far no well-sampled genetic research results has been published and the comprehensive phylogenetic studies on Habenariinae were limited to African genus *Bonatea* (Ponsie et al., 2007), and Neotropical representatives of *Habenaria* s.l. (Batista et al., 2013). The studies by Jin et al. (2014) revealed that the taxonomy of *Habenaria* is even more complicated than previously thought, but due to the limited sampling the authors did not propose any new classification of the genus. The most recent research on phylogeny of African genera of subtribe Orchidinae s.l. (Ngugi et al., 2020) also were inconclusive in context of infrageneric division of *Habenaria*.

Moreover, *Habenaria* representatives can be easily confused with species of two other genera reported from New Guinea—Australasian *Peristylus* Blume and *Platanthera* Rich. which is widely spread in Northern hemisphere. The general flower architecture are very similar in all these genera. Their petals are more or less agglutinate to dorsal sepal forming a prominent galea over the gynostemium, the lateral sepals are usually reflexed, and the pendent lip is basally elongated into spur of various form and length. Admittedly, the lip of *Platanthera*, unlike *Peristylus* and most *Habenaria* species, is simple, ligulate to elliptic, but the substantial distinguishing character amongst them is the gynostemium structure. The examination of this tenuous organ requires detailed microscopical observation, but provides the only, and certain discriminative evidence that permits separation of *Habenaria*, *Platanthera* and *Peristylus*. The first significant character separating *Habenaria* from both other genera is the form of lateral stigma lobes which are elongate and in major or minor parts free from the lip margins. In *Platanthera* and *Peristylus* lateral stigma lobes are completely fused with basal part of the lip. Rostellum of *Habenaria* is deeply 3-lobed with the middle one pushed between loculae, and distal parts of lateral lobes being elongated and free from the lip margins. In *Platanthera* the rostellum is similarly 3-lobed, but with lateral lobes being completely incorporated in the basal part of gynostemium. The rostellum of *Peristylus* is essentially different, *i.e*. it forms a shelf-like barrier between receptive surface and anther. Distal parts of anther loculae of *Habenaria* are usually greatly elongated and free from the lip, what is correlated with usually longer caudiculae. Concededly, in *Platanthera* apical parts of loculae are elongated, but they are fully joined with the gynostemium basal part. There are no elongate apical parts of anther loculae in *Peristylus*. In the representatives of this genus loculae are ellipsoid or ovoid, and caudiculae are very short, often hardly noticeable. Of course we can list some more differences between *Habenara*, *Platanthera* and *Peristylus*, as relation in size of the viscidium and caudiculae, width of connective, etc, but they have limited significance in distinguishing the three genera.

*Habenaria* s.l. has been intensively studied in the aspect of morphological variation. The research resulted in proposal of separation from *Habenaria* s.l. several new, smaller genera, *e.g. Arachnaria* Szlach., *Ceratopetalorchis* Szlach., Górniak & Tukałło, *Macrura* Szlach. & Sawicka, *Mirandorchis* Szlach. & Kras, *Monadeniorchis* Szlach. & Kras, *Podandriella* Szlach., *Pseudocoeloglossum* Szlach., *Rhinorchis* Szlach., *Trachypetalum* Szlach. & Sawicka (*e.g*. Szlachetko, 2003; Szlachetko & Sawicka, 2003; Szlachetko & Kras, 2006), which however, gain no acceptance by the wider taxonomic audience due to the lack of molecular evidence for the separateness of these taxa. In New Guinea and adjacent areas wide morphological variation could be observed as well. We distinguished four main morphological groups with regard to vegetative parts, such as general habit and leaf arrangement, and structure of flower focusing on petals and lip. Some of the species concerned were classified recently under different genera like *Medusorchis* Szlach. (*H. notabilis*), *Pecteilis* Raf. (*H. baeuerlenii, H. elongata, H. ochroleuca, H. rumphii*), and *Plantaginorchis* Szlach. (*H. baeuerlenii*) however since we are far from understanding the complexity of the genus *Habenaria*, we treat them in the broad sense of the genus. The phylogenetic relationship between New Guinean *Habenaria* remains unknown due to lack of molecular data.

Among more than 200 species from Asia and Oceania, over 160 Neotropical, and about 300 African representatives of *Habenaria* s.l. we did not find any significant variation of the lip or petals lobation as well as in relative spur length within the species. The latter character was recognized as consistent when the spur length is compared to the lip size, however the ratio between spur and ovary length may be different in the flowers of the lowermost and the uppermost part of the inflorescence. We also did not find significant variation of vegetative characters and tepals shape within the species.

However, during our studies we found that some species are characterized by great variation of the floral characters. Dissimilarities of lip and spur shape were observed even among the flowers of the same specimen and they do not seem to be related with the flower position in the inflorescence. Amongst New Guinea species *H. baeuerlenii* shows broad variation in shape of lip lateral lobes and spur length which is usually 12–13 mm long, but some specimens (*e.g. Sands 1158*) represent a short-spurred form, only 4.9 mm long. Because this species is broadly distributed in New Guinea, this variation may be a result of the overall phenotypic plasticity and adaptation to different habitats. However, because some authors already described monstrous development of flowers in some *Habenaria* and related taxa (Henslow, 1858; Bryan, 1917; Kurzweil, 2009) and we cannot exclude the possibility of ubnormal deverlopment of these short-spurred flowers in *H. baeuerlenii*.

While the lip and petals shape were traditionally used as the main characters in the taxonomic studies on *Habenaria* (*e.g*. Szlachetko, 2003, 2005, 2012) and its relatives, our research revealed that not all species of Habenariinae can be classified in any infrageneric taxon based on the former traits. Moreover, the relative spur length cannot be always used as diagnostic at the species level. The most conservative characters seems to be the leaf arrangement and shape, tepals shape and gynostemium morphology and those traits should have priority in the infrageneric classification of *Habenaria*. The form and size of antherophores, stigmatophores, rostellophores are conservative at species level.

The inaccurate examination of the herbarium specimens of Habenariiinae representatives may lead to redundant descriptions of new taxa. This scenario was observed when Dockrill (1965) described *Habenaria anomala* – the spurless orchid resembling *H. xanthantha*. We examined the type specimens of the latter species and we found flowers with prominent spurs arranged in the same inflorescence along with flowers characterized by spur reduction. In 2009 a new species of *Habenaria*, *H. anomaliflora*, from Asia was described (Kurzweil, Chantanaorrapint & Buakhla, 2009). This taxon is known so far from four localities in Thailand and Laos. It is characterized by the subactinomorphic perianth lacking a lip spur and gynostemium morphology not observed in any other representative of *Habenaria* s.l. In the four species characterized above we did not find any abnormalities in the gynostemium structure in any of the examined flowers. Both the tepals and the lip of *H. anomaliflora* resemble those observed in *H. reniformis* are we believe that the specimens listed by Kurzweil, Chantanaorrapint & Buakhla (2009) are not more than peloric individuals of the latter orchid.

## Conclusions

Here we evaluated the diversity of *Habenaria* s.l. in New Guinea and adjacent areas. The species characteristics and identification keys provided will be useful for local scientists working on flora inventories and for introducing more sophisticated nature management programs.

We confirmed the occurrence of 27 *Habenaria* species in the study area including *H. devogeliana* described in this paper. Sixteen genus representatives are endemic to New Guinea.

## Supplemental Information

10.7717/peerj.12011/supp-1 Supplemental Information 1 List of reference herbarium specimens.

## Additional Information and Declarations

### Data Availability

The following information was supplied regarding data availability: All relevant information are included in the text. Herbarium specimens are identified by collector number and a list of reference herbarium specimens is available as a Supplemental File.

### New Species Registration

The following information was supplied regarding the registration of a newly described species: *Habenaria devogeliana* Kolan., Szlach., Kras & S. Nowak, sp. nov. LSID: 77219673-1.
